# Molecular Eigensolution Symmetry Analysis and Fine Structure

**DOI:** 10.3390/ijms14010714

**Published:** 2013-01-04

**Authors:** William G. Harter, Justin C. Mitchell

**Affiliations:** 1 Department of Physics, University of Arkansas, Fayetteville, AR 72701, USA; 2 Intel Corporation, Santa Clara, CA 95054, USA; E-Mail: nanojustin@gmail.com

**Keywords:** symmetry, molecular dynamics, tunneling, level clusters

## Abstract

Spectra of high-symmetry molecules contain fine and superfine level cluster structure related to *J*-tunneling between hills and valleys on rovibronic energy surfaces (RES). Such graphic visualizations help disentangle multi-level dynamics, selection rules, and state mixing effects including widespread violation of nuclear spin symmetry species. A review of RES analysis compares it to that of potential energy surfaces (PES) used in Born–Oppenheimer approximations. Both take advantage of adiabatic coupling in order to visualize Hamiltonian eigensolutions. RES of symmetric and *D*_2_ asymmetric top rank-2-tensor Hamiltonians are compared with *O_h_* spherical top rank-4-tensor fine-structure clusters of 6-fold and 8-fold tunneling multiplets. Then extreme 12-fold and 24-fold multiplets are analyzed by RES plots of higher rank tensor Hamiltonians. Such extreme clustering is rare in fundamental bands but prevalent in hot bands, and analysis of its superfine structure requires more efficient labeling and a more powerful group theory. This is introduced using elementary examples involving two groups of order-6 (*C*_6_ and *D*_3_~*C*_3_*_v_*), then applied to families of *O_h_* clusters in *SF*_6_ spectra and to extreme clusters.

## 1. Overview of Eigensolution Techniques for Symmetric Molecules

A key mathematical technique for atomic or molecular physics and quantum chemistry is matrix diagonalization for quantum eigensolution. As computers become faster and more available, more problems of chemical physics are framed in terms of choosing bases for eigensolution of time evolution operators or Hamiltonian generator matrices. The resulting eigenvectors and eigenvalues are Fourier amplitudes and frequencies that combine to give all possible dynamics in a given basis choice.

Despite the increasing utility and power of computer diagonalization, it remains a “black box” of processes quite unlike the complex natural selection by wave interference that we imagine nature uses to arrive at its quantum states. Diagonalization uses numerical tricks to reduce each *N*-by-*N* matrix to *N* values and *N* stationary eigenstates, but the artificial processes may seem as opaque as nature itself with little or no physical insight provided by *N*_2_ − *N* eigenvector components. We are thus motivated to seek ways to visualize more of the physics of molecular eigensolutions and their spectra. This leads one to explore digital graphical visualization techniques that provide insight as well as increased computational power and thereby complement numerically intensive approaches [1].

Before describing tensor eigensolution techniques and rovibronic energy surfaces (RES), a brief review is given of potential energy surface (PES) to put the tensor RES in a historical and methodological context. This includes some background on semiclassical approximations of tensor algebra that help explain rotational level clustering and are used to develop the RES graphical tools. Section 2 reviews how RES apply to symmetric and asymmetric top molecules. This serves to motivate the application of RES to more complicated molecules of higher symmetry. Section 3 contains a graphical analysis of octahedral RES and an introductory review of level clusters (fine structure) having 6-fold and 8-fold quasi-degeneracy (superfine structure) due to rank-4 tensor Hamiltonians. Following this is a discussion of mixed-rank tensors that exhibit 12-fold and 24-fold monster-clusters. The latter have only recently been seen in highly excited rovibrational spectra [2] and present challenging problems of symmetry analysis to sort out a plethora of tunneling resonances and parameters for so many resonant states.

Following introductory Section 4, these problems are addressed in Sections 6–8 by redeveloping group algebraic symmetry analysis into a more physically direct and elegantly powerful approach. It uses underlying duality between internal and external symmetry states and their operations. Duality is introduced using the simplest order-6 symmetry groups *C*_6_ and *D*_3_~*C*_3_*_v_* before applying it to *O_h_* symmetric monster-clusters in Section 8. Monsters in REES-polyad bands are shown in final Section 9.

The direct approach to symmetry starts by viewing a group product table as a Hamiltonian matrix *H* representing an **H** operator that is a linear combination of group operators **g***_k_* with a set of ortho-complete tunneling coefficients *g_k_* labeling each tunneling path. A main idea is that symmetry operators “know” the eigensolutions of their algebra and thus of all Hamiltonian and evolution operators made of **g***_k_*’s.

### 1.1. Computer Graphical Techniques

Several graphical techniques and procedures exist for gaining spectral insight. One of the oldest is the Born–Oppenheimer approximate (BOA) potential energy surface (PES) that is a well-established tool for disentangling vibrational-electronic (vibronic) dynamics. While BOA-PES predate the digital age by decades, their calculation and display is made practical by computer. More recent are studies of phase portraits and wavepacket propagation techniques to follow high-*ν* vibrational dynamics and chemical pathways for dissociation or re-association [3,4]. This includes BOA-breakdown states in which a system evolves on multiply interfering PES paths. Dynamic Jahn–Teller–Renner effects involve multi-BOA-PES states in molecules and solids. Examples in recent works [5,6] include coherent photo-synthesis [7].

Visualizing eigensolutions and spectra in crystalline solids is helped by bands of dispersion functions in reciprocal frequency-versus-wavevector space. Fermi-sea contours are used to analyze de Haas–van Alphen effects and more recently in understanding quantum Hall effects. Analogy between band theory of solids and molecular rovibronic clusters is made in Section 4 and 7.

Visualization of molecular rotational, rovibrational, and rovibronic eigensolutions and spectra is the subject of this work and involves the rotational energy surface (RES). As described below, an RES is a multipole expansion plot of an effective Hamiltonian in rotational momentum space. Ultra sensitivity of vibronic states to rotation lets the RES expose subtle and unexpected physics. Multi-RES or rovibronic energy eigenvalue surfaces (REES) have conical intersections analogous to Jahn–Teller PES (See Section 9).

The RES was introduced about thirty years ago [8] to analyze spectral fine structure of high resolution spectral bands in molecules of high symmetry including *PH*_3_ [9]*XDH*_3_ and *XD*_2_*H* molecules [10], tetrahedral (*P*_4_) [11], tetrafluorides (*CF*_4_ and *SiF*_4_) [12], hexafluorides (*SF*_6_, *Mo*(*CO*)_6_ and *UF*_6_) [13–16], cubane (*C*_8_*H*_8_), and buckyball (*C*_60_) [15,16] and predicted major mixing of Herzberg rovibronic species. Recently RES have been extended to help understand the dynamics and spectra of fluxional rotors [17] or “floppy” molecules such as methyl-complexes [18] and vibrational overtones [2].

Each of the techniques and particularly the RES-based ones described below depend upon the key wave functional properties of stationary phase, adiabatic invariance, and the spacetime symmetry underlying quantum theory. Additional symmetry (point group, space group, exchange, gauge, *etc*.) of a molecular system introduces additional resonance. Symmetry tends to make graphical techniques even more useful since they help clarify resonant phenomenal dynamics and symmetry labeling [19].

#### 1.1.1. Vibronic Born–Openheimer Approximate Potential Energy Surfaces (BOA-PES)

A BOA-PES depends on an adiabatic invariance of each electronic wavefunction to nuclear vibration. It is often said that the electrons are so much faster than nuclei that the system “sticks” to a particular PES that electrons provide. Perhaps a better criterion would be that the Fourier spectrum associated with nuclear motion does not overlap that of an electronic transition to another energy level. Nuclei often provide stable configurations that quantize electronic energy into levels separated by gaps much wider than that of low lying vibrational “phonon” states.

A BOA wavefunction is a peculiarly entangled outer product Ψ = *ηψ* of a nuclear factor wavefunction *η_ν_*_(_*_ε_*_)_ (*X . . .* ) whose quantum labels *ν*(*ε*) depend on electronic quantum numbers *ε* = *nlm*, *etc*. while the electronic factor wave *ψ* (*x*_(_*_X..._*_)_ . . . ) is a function whose electron coordinates *x*_(_*_X..._*_)_ . . . depend adiabatically on nuclear vibrational coordinates (*X . . .* ) of PES *V_ε_*(*X . . .* ) for electron bond state *ε*.

(1a) $$\Psi_{\nu (ɛ)}(x^{electron}\ldots X^{nuclei}\ldots )=\psi_{ɛ}(x_{\left(X\ldots \right)}\ldots )\cdot \eta_{\nu (ɛ)}(X\ldots )\;\;\;\text{BOA}-\text{Entangled Product}$$

(1b) $$\Psi_{\nu ,ɛ}(x^{electron}\ldots X^{nuclei}\ldots )=\psi_{ɛ}(x\ldots )\cdot \eta_{\nu }(X\ldots )\;\;\;\text{Unentangled Product}=\langle x\ldots |\psi_{ɛ}\rangle \langle X\ldots |\eta_{\nu }\rangle =\langle x\ldots ;X\ldots |\psi_{ɛ};\eta_{\nu }\rangle$$

The adiabatic convenience of a single product Equation (1a) with a vibration eigenfunction *η_ν_*_(_*_ε_*_)_(*X . . .* ) on a single PES function *V_ε_*(*X . . .* ) is welcome but comes at a price; a BOA-entangled coordinate-state is not a simple bra-ket wavefunction product Equation (1b) of bra-bra 〈*x . . .* |〈*X . . .* | position and ket-ket | *ψ_ε_*〉|*η_ν_*〉 state. Symmetry operator product analysis of Equation (1b) is well known. Symmetry of Equation (1a) depends on rotational BOA-relativity of its parts. Vibronic BOA-PES generalize to rovibronic RES by accounting for rotational relations.

#### 1.1.2. Rovibronic BOA Rotational Energy Surfaces (BOA-RES)

The rotational energy surface (RES) can be seen as a generalization of adiabatic BOA wave Equation (1a) to Equation (2) below that includes rotational motion. Here one treats vibronic motion as having the “fast” degrees of freedom while rotational coordinates Θ (e.g., Euler angle (*αβγ*) for semi-rigid molecules) play the “slow” semi-classical role vis-a-vis the “faster” adiabatic vibration or vibronic states.

(2) $$\Phi_{J[\nu (ɛ)]}(x^{elec}\ldots Q^{vib}\ldots \Theta^{rot})=\psi_{ɛ}(x_{\left(Q\ldots \Theta \ldots \right)}\ldots )\cdot \eta_{\nu (ɛ)}(Q\ldots [\Theta \ldots ])\cdot \rho_{J[\nu (ɛ)]}(\Theta^{rot}\cdots )$$

In Equation (2), the wave factors of each motion are ordered fast-to-slow going left-to-right. As in Equation (1a) each wave-factor quantum number depends on quanta in “faster” wave-factors written to its left, but each coordinate has adiabatic dependence on coordinates in “slower” factors written to its right.

The *Q* in Equation (2) denotes vibrational normal coordinates (*q*_1_*, q*_2_*, . . . q_m_*) and *ν* denotes their quanta (*ν*_1_*, ν*_2_*, . . . ν_m_*). The number *m* = 3*N* − 6 of modes of an *N*-atom semi-rigid molecule has subtracted 3 translational and 3 rotational coordinates. Each mode *q_k_* assumes an adiabatic BOA dependency on overall translation and rotation Θ known as the Eckart conditions. (Here we will ignore translation.)

RES are multipole expansion plots of effective BOA energy tensors for each quantum value of vibronic *ν*(*ε*) and conserved total angular momentum *J*. Choices of effective energy tensors depend on the level of adiabatic approximation. So do the choices of spaces in which RES are plotted. Elementary examples of model BOA waves, tensors, and RES for rigid or semi-rigid molecules are discussed below.

### 1.2. Lab-Frame Coupling vs. Body Frame Constriction

Wave *ρ_J_*(Θ*^rotation^*) for a bare rigid symmetric-top (*ψ* = 1 = *η*) molecule is a Wigner *D^J^* -function.

(3) $$\rho_{J}(\Theta )=\rho_{J,M,K}(\alpha \beta \gamma )=D_{M,K}^{J*}(\alpha \beta \gamma )\sqrt{\text{norm}}\;\;\;\text{norm}=[\text{J}]=2\text{J}+1$$

Total angular momentum *J* is *J* = *R* for a bare rotor. Bare lab-frame z-component is labeled*M* = *m*. Its body-frame *z̄*-component is labeled *K* = *M̄* = *n. m* and *n* range from +*R* to −*R* in integral steps.

Entangled BOA product Equation (2) mates vibronic factor Equation (1a) with a rotor factor *ρ_J_* = *ρ_J,MmK_* in Equation (3). Now *J* and *K* = *M̄* depend on total vibronic momentum *l* and its body *z̄-*component *μ̄* in Ψ*_ν_*_(_*_ε_*_)_ = Ψ*_μ̄_^l^*.

(4) $$\Phi_{J[\nu (ɛ)]}=\Psi_{\nu (ɛ)}^{l}\cdot \rho_{J[\nu (ɛ)]}=\Psi_{\bar{\mu }}^{l}\cdot \rho_{J,M,K}=\Psi_{\bar{\mu }}^{l}\cdot D_{M,K}^{J*}\sqrt{\left[J\right]}$$

Disentangled product Ψ*_ρ_* in Equation (1b) of lab-based vibronic wave Ψ*_μ̄_^l^* and bare rotor *ρ_R,m,n_* of Equation (3) is coupled by Clebsch–Gordan Coefficients *C_μmM_^lRJ^* into a wave Φ*_M_^J^* of total *J* = *R* + *l*, *R* + *l* − 1*, . . .* or |*R* − *l*| and *M* = *μ* + *m* by sum Equation (5) over lab *z*-angular bare rotor momenta *m* and lab vibronic *μ* bases.

(5) $$\Phi_{M}^{J}=\sum_{\mu ,m}C_{\mu mM}^{lRJ}\psi_{\mu }^{l}\cdot \rho_{m}^{R}=\sum_{\mu ,m}C_{\mu mM}^{lRJ}\psi_{\mu }^{l}\cdot D_{m,n}^{R*}\sqrt{\left[R\right]}\;\;\;(\text{M}=\mu +\text{m}=\text{const}.)$$

A BOA-entangled wave in Equation (1a) or Equation (4) requires more serious surgery in order to survive as a viable theoretical entity. BOA vibronic waves are not merely coupled as in Equation (5) to a rotor, they are adiabatically “glued” or constricted to the intrinsic molecular rotor frame. (A rotor is “BOA-constricted” by its vibronic wave much as a boa-constrictor rides its writhing prey as the two rotate together.)

A remarkable property of quantum rotor operator algebra is that Wigner *D^l^*-waves in Equation (3) are also transformation matrices that relate rotating body-fixed BOA Ψ*_μ̄_^l^* (*body*) into the lab-fixed Ψ*_μ_^l^* (*lab*).

(6a) $$\Psi_{\bar{\mu }}^{l}(body)=\sum_{\mu }\Psi_{\mu }^{l}(lab)D_{\bar{\mu }\mu }^{l}(\alpha \beta \gamma )$$

(6b) $$\Psi_{\mu }^{l}(lab)=\sum_{\bar{\mu }}\Psi_{\bar{\mu }}^{l}(body)D_{\mu \bar{\mu }}^{l*}(\alpha \beta \gamma )$$

This rotational wave relativity is a subset of Lorentz–Einstein–Minkowski space-time-frame relativity that uses symmetry algebra to keep track of the invariant sub-spaces (eigensolutions). D-Matrices underlie all tensor operators, their eigenfunctions and their eigenvalues and are a non-Abelian (non-commutative) generalization of plane waves d^k*^ (*r*) = 〈*r*|*k*〉 = *e^ikr^* underlying Fourier operator analysis. Details of this connection comprise the later Section 5.

Of particular importance to RES theory is the Wigner–Eckart factorization lemma that relates Clebsch–Gordan *C_μmM_^lRJ^* to Wigner-*D*’s and transforms coupled wave Equation (5) to BOA-constricted wave Equation (4).

(7a) $$\int d(\alpha \beta \gamma )D_{\mu \bar{\mu }}^{l*}(\alpha \beta \gamma )D_{mn}^{R*}(\alpha \beta \gamma )D_{MK}^{J}(\alpha \beta \gamma )=\frac{1}{\left[J\right]}C_{\mu mM}^{lRJ}C_{\bar{\mu }nK}^{lRJ}$$

(7b) $$\sum_{\mu }\sum_{\bar{\mu }}C_{\mu mM}^{lRJ^{\prime }}D_{\mu \bar{\mu }}^{l*}(\alpha \beta \gamma )D_{mn}^{R*}(\alpha \beta \gamma )C_{\bar{\mu }nK}^{lRJ}=\delta^{JJ^{\prime }}D_{MK}^{J*}(\alpha \beta \gamma )$$

(7c) $$\sum_{\mu }C_{\mu mM}^{lRJ^{\prime }}D_{\mu \bar{\mu }}^{l*}(\alpha \beta \gamma )D_{mn}^{R*}(\alpha \beta \gamma )=\sum_{\bar{\mu }}C_{\bar{\mu }nK}^{lRJ}D_{MK}^{J*}(\alpha \beta \gamma )$$

A more familiar form of this is the Kronecker relation of product reduction $D^{l}⊗D^{R}\approx D^{J}⊕D^{J^{\prime }}⊕\cdots$. Another form is a body-to-lab coupling relation with *M* = *μ* + *m* and *n* = *K* − *μ̄* fixed in the *μ* or *μ̄* sums. The latter yields a sum over *μ̄* = *K* − *n* of body-fixed BOA waves Equation (5) giving lab-based Φ*_M_^J^* wave Equation (4).

(8) $$\Phi_{M}^{J}=\sum_{\mu }C_{\mu mM}^{lRJ}\Psi_{\mu }^{l}(lab)D_{m,n}^{R*}\sqrt{\left[R\right]}$$

(9) $$\begin{array}{r} \Phi_{M}^{J}=\sum_{\mu }C_{\mu mM}^{lRJ}\sum_{\bar{\mu }}\Psi_{\mu }^{l}(body)D_{\mu \bar{\mu }}^{l*}D_{m,n}^{R*}(\alpha \beta \gamma )\sqrt{\left[R\right]}\\ =\sum_{\mu }C_{\bar{\mu }nK}^{lRJ}\Psi_{\bar{\mu }}^{l}(body)D_{MK}^{J*}(\alpha \beta \gamma )\sqrt{\left[R\right]}\\ =\sum \bar{\mu }C_{-K\bar{\mu }n}^{JlR}\Psi_{\bar{\mu }}^{l}(body)D_{MK}^{J*}(\alpha \beta \gamma )\sqrt{\left[J\right]} \end{array}$$

(10a) $$\Phi_{M}^{J}=\sum_{\bar{\mu }}C_{-K\bar{\mu }n}^{JlR}\Psi_{\bar{\mu }}^{l}\rho_{J,M,K}=\sum_{\bar{\mu }}C_{-K\bar{\mu }n}^{JlR}\Phi_{J[K\nu (ɛ)]}$$

(10b) $$\Phi_{J[K\nu (ɛ)]}==\sum_{R}C_{-K_{\mu n}}^{JlR}\Phi_{M}^{J}$$

Body-(un)coupling in Equation (10a) is an undoing of BOA-constriction by subtracting vibronic (*l, μ̄*) from (*J,K*) of BOA-wave Φ*_J_*_[_*_ν_*_(_*_ε_*_)]_ in Equation (10b) to make lab-fixed Φ*_M_^J^* in Equation (10a) with sharp rotor quanta *R* = *J* − *l, J* − *l* + 1 . . . or *J* + *l*. In a lab-fixed wave Φ*_M_^J^* of Equation (5) or (10a) rotor R is conserved but *K* and *μ̄* are not. A BOA wave Φ*_J_*_[_*_ν_*_(_*_ε_*_)]_ of Equation (4) or (10b) has body-fixed vibronic *K* and *μ̄* that are conserved but rotor *R* is not.

Note the following for Equations (8)–(10b). For Equation (8) we have constant (*M* = *μ* +*m*). Result Equation (9) is derived from Equations (6b) and (7c) with constant (*n* = *K* − *μ̄*). In Equation (10a)*K* = *μ̄* + *n*. In Equation (10b)*M* = *μ* + *m*.

However, in both Equation (10a) and (10b) the internal bare-rotor body component *n* = *K* − *μ̄* is conserved due to a symmetric rotor’s azimuthal isotropy. This *n* is a basic rovibronic-species quantum number invariant to all lab based perturbation or transition operators. Like a gyro in a suitcase, no amount of external kicking of the case will slow its spin. Only internal body operations can “brake” its *n*.

The duality of lab *vs*. body quantum state labels and external *vs*. internal operators is an important feature of molecular and nuclear physics, and it is to be respected if we hope to take full advantage of symmetry group algebra of eigensolutions. The duality is fundamental bra-&-ket relativity. For every group of symmetry operations such as a 3D rotation group *R*(3)*_lab_* = {. . .**R**(*αβγ*) . . . } there is a dual body group *R*(3)*_body_* = {. . . **R̄**( *αβγ*) . . . } having identical group structure but commuting with the lab group. Tensor multipole operators, discussed next, come in dual and inter-commuting sets as well. Generalized Duality is key to efficient symmetry analysis as shown beginning in Section 6.1.

### 1.3. Mathematical Background for Tensor Algebra

#### 1.3.1. Unitary Multipole Functions and Operators

Spherical harmonic functions *Y_m_^l^* (*φθ*) are well know orbital angular factors in atomic and molecular physics. They are special (*n* = 0)-cases of Wigner-*D^l^* functions Equation (3) as follows.

(11) $$Y_{m}^{l}(\varphi \theta )=D_{m,0}^{l*}(\varphi \theta 0)\sqrt{\frac{\left[l\right]}{4\pi }}\;\;\;\text{where};[l]=2l+1$$

A diatomic or linear rotor must have zero body quanta (*n* = 0) and has a *Y_m_^l^*(*φθ*) rotor wave. *Y_m_^l^* -matrix elements or expectation values of a multipole potential *Y_q_^k^* are proportional to Clebsch forms of Equation (7a).

(12) $$\int d(\varphi \theta 0)D_{m^{\prime }0}^{J^{\prime }}(\varphi \theta 0)D_{q0}^{k*}(\varphi \theta 0)D_{m0}^{J*}(\varphi \theta 0)=\sqrt{\frac{{\left(4\pi \right)}^{3}}{\left[J^{\prime }][k][J\right]}}\int d(\varphi \theta )Y_{m^{\prime }}^{J*}Y_{q}^{k}Y_{m}^{J}=\frac{1}{\left[J\right]}C_{qmm^{\prime }}^{kJJ^{\prime }}C_{000}^{kJJ^{\prime }}$$

A multipole **v***_q_^k^* matrix is Equation (12) with factor 〈*J*′||*k*||*J*〉 depending on {*J*′*, k, J*} but not {*m*′*, q,m*}.

(13a) $$\langle \begin{array}{c} J^{\prime }\\ m^{\prime } \end{array}|v_{q}^{k}|\begin{array}{c} J\\ m \end{array}\rangle =C_{qmm^{\prime }}^{kJJ^{\prime }}\langle J||k||J\rangle$$

Factor 〈*J*′||**v***_q_^k^* ||*J*〉 is the reduced matrix element of **v**_q_^k^ and chosen by a somewhat arbitrary convention.

(13b) $$\langle J^{\prime }||v^{k}||J\rangle ={\left(-1\right)}^{k+J^{\prime }-J}\sqrt{\frac{\left[J^{\prime }\right]}{\left[k\right]}}$$

This particular choice simplifies bra-ket coupling and creation-destruction operator expressions for **v***_q_^k^*.

(13c) $$\begin{array}{r} v_{q}^{k}={\left(-1\right)}^{2J^{\prime }}\sum_{\begin{array}{l} m,m^{\prime }\\ =q-m \end{array}}C_{m^{\prime }mq}^{J^{\prime }Jk}|\begin{array}{c} J^{\prime }\\ m^{\prime } \end{array}\rangle {|\begin{array}{c} J*\\ m \end{array}\rangle }^{†}\\ ={\left(-1\right)}^{2J^{\prime }}\sum_{\begin{array}{l} m,m^{\prime }\\ =q-m \end{array}}C_{m^{\prime }mq}^{J^{\prime }Jk}|\begin{array}{c} J^{\prime }\\ m^{\prime } \end{array}\rangle \langle \begin{array}{c} J\\ -m \end{array}|{\left(-1\right)}^{J-m}\\ =\sum_{\begin{array}{l} m,m^{\prime }\\ =q+m \end{array}}{\left(-1\right)}^{J^{\prime }-m^{\prime }}\sqrt{\left[k\right]}\left(\begin{array}{ccc} k & J & J^{\prime }\\ q & m & -m^{\prime } \end{array}\right)\;{\bar{a}}_{m}^{J^{\prime }},{\bar{a}}_{m}^{J} \end{array}$$

Other choices rescale **v***_q_^k^* eigenvalues but do not affect eigenvectors of a tensor **v***_q_^k^* or its transformation behavior Equation (14). (By Equations (7c) and (13c), **v***_q_^k^* transforms like Equation (6a) for a wave function *Y_q_^k^* (*φθ*).)

(14) $${\bar{v}}_{q}^{k}=R(\alpha \beta \gamma )v_{q}^{k}R^{†}(\alpha \beta \gamma )=\sum_{q=-k}^{k}v_{\bar{q}}^{k}D_{\bar{q}q}^{k}(\alpha \beta \gamma )$$

Examples of **v***_q_^k^* tensor matrices for *J*′ = *J* = 1 to 3 are given in Table 1. The *J* = 2 case is given in expanded form by Table 1. (Higher-J tables are *q*-folded to save space. Scalar ${\langle v_{0}^{0}\rangle }^{J}=1/\sqrt{\left[J\right]}$ is left off each *J*-table in Table 2)

Historically, spinor *J* = 1*/*2 tensors shown in Table 3(a) are related to four Pauli spinor matrices *σ_μ_* and Hamilton quaternions {**1***,***i***,***j***,***k**} in Table 3(b) or Table 3(c). The latter appear in 1843 and are used for Stokes’ polarization theory in 1867. The *σ_μ_* are *U*(2) algebraic basis of quantum theory for physics ranging from sub-kHz NMR to TeV hadrons and also applies to relativity. General *U*(*k*) algebra has *k*^2^ generators **v**_0_^0^*,***v***_q_*^1^*, . . . ,***v***_q_^k^* with a subset of *k* mutually commuting diagonal (*q* = 0) labeling operators **v**_0_*^k^* of the *U*(*k*) tensor algebras. The **v***_q_^k^* are related to elementary creation-destruction *e_jk_* = *a_j_*^†^*a_k_*-operators and to their RES in the following sections.

#### 1.3.2. Tensor and Elementary Matrix Operators

Coefficient $\langle \begin{array}{c} J\\ m^{\prime } \end{array}|v_{q}^{k}|\begin{array}{c} J\\ m \end{array}\rangle$ of elementary operator $e_{m^{\prime },m}=|\begin{array}{c} J\\ m^{\prime } \end{array}\rangle \langle \begin{array}{c} J\\ m \end{array}|$ is the following CG orWigner 3-j.

(15a) $${\langle v_{q}^{k}\rangle }^{J}=\sum_{m^{\prime },m}\langle \begin{array}{c} J\\ m^{\prime } \end{array}|v_{q}^{k}|\begin{array}{c} J\\ m \end{array}\rangle |\begin{array}{c} J\\ m^{\prime } \end{array}\rangle \langle \begin{array}{c} J\\ m \end{array}|=\sum_{m^{\prime },m}\langle \begin{array}{c} J\\ m^{\prime } \end{array}|v_{q}^{k}|\begin{array}{c} J\\ m \end{array}\rangle {\langle e_{m^{\prime }m}\rangle }^{J}\;\text{where}:q=m^{\prime }-m$$

(15b) $$\langle \begin{array}{c} J^{\prime }\\ m^{\prime } \end{array}|v_{q}^{k}|\begin{array}{c} J\\ m \end{array}\rangle ={\left(-1\right)}^{J^{\prime }+m^{\prime }}\sqrt{\left[k\right]}\left(\begin{array}{ccc} k & J & J^{\prime }\\ q & m & -m^{\prime } \end{array}\right)={\left(-1\right)}^{J^{\prime }+J-k}\sqrt{\frac{\left[k\right]}{\left[J^{\prime }\right]}}C_{qmm^{\prime }}^{kJJ^{\prime }}$$

Each matrix 〈**v***_q_^k^*〉*^J^* for *J*′ = *J* = 1 *to* 5 is displayed in compressed form by the following tensor representation Table 2.

CG-3j relation Equation (13c) implies 〈**v***_q_^k^*〉*^J^* and 〈**e***_m_*_′_*_,m_*〉*^J^* matrices have ortho-complete unit vectors of dimension *d*(*J, q*) = [*J*] − *q* = 2*J* − *q* + 1 along *q^th^*-diagonal of each [*J*]-by-[*J*] matrix. For example, quadrupole **v**_2_^2^, octopole **v**_2_^3^, and 2^4^-pole **v**_2_^4^ share the *q* = 2 diagonal of *J* = 2 Table 2.

(16a) $$\begin{array}{cc} \begin{array}{c} {\langle v_{q=\pm 2}^{2}\rangle }^{J=2}=\sqrt{\frac{2}{7}}{\langle e_{-2,0}\rangle }^{J=2}\\ \left|\begin{array}{ccccc} \cdot & \cdot & \sqrt{\frac{2}{7}} & \cdot & \cdot \\ \cdot & \cdot & \cdot & \sqrt{\frac{3}{7}} & \cdot \\ \cdot & \cdot & \cdot & \cdot & \sqrt{\frac{2}{7}}\\ \cdot & \cdot & \cdot & \cdot & \cdot \\ \cdot & \cdot & \cdot & \cdot & \cdot \end{array}\right|=\sqrt{\frac{2}{7}}\left|\begin{array}{ccccc} \cdot & \cdot & 1 & \cdot & \cdot \\ \cdot & \cdot & \cdot & \cdot & \cdot \\ \cdot & \cdot & \cdot & \cdot & \cdot \\ \cdot & \cdot & \cdot & \cdot & \cdot \\ \cdot & \cdot & \cdot & \cdot & \cdot \end{array}\right| \end{array} & \begin{array}{c} +\sqrt{\frac{2}{7}}{\langle e_{-1,1}\rangle }^{J=2}+\sqrt{\frac{2}{7}}{\langle e_{0,2}\rangle }^{J=2}\\ +\sqrt{\frac{3}{7}}\left|\begin{array}{ccccc} \cdot & \cdot & \cdot & \cdot & \cdot \\ \cdot & \cdot & \cdot & 1 & \cdot \\ \cdot & \cdot & \cdot & \cdot & \cdot \\ \cdot & \cdot & \cdot & \cdot & \cdot \\ \cdot & \cdot & \cdot & \cdot & \cdot \end{array}\right|+\sqrt{\frac{2}{7}}\left|\begin{array}{ccccc} \cdot & \cdot & \cdot & \cdot & \cdot \\ \cdot & \cdot & \cdot & \cdot & \cdot \\ \cdot & \cdot & \cdot & \cdot & 1\\ \cdot & \cdot & \cdot & \cdot & \cdot \\ \cdot & \cdot & \cdot & \cdot & \cdot \end{array}\right| \end{array}\\ \begin{array}{c} {\langle v_{q=\pm 2}^{3}\rangle }^{J=2}=\sqrt{\frac{1}{2}}{\langle e_{-2,0}\rangle }^{J=2}\\ \left|\begin{array}{ccccc} \cdot & \cdot & \sqrt{\frac{1}{2}} & \cdot & \cdot \\ \cdot & \cdot & \cdot & 0 & \cdot \\ \cdot & \cdot & \cdot & \cdot & -\sqrt{\frac{1}{2}}\\ \cdot & \cdot & \cdot & \cdot & \cdot \\ \cdot & \cdot & \cdot & \cdot & \cdot \end{array}\right|=\sqrt{\frac{1}{2}}\left|\begin{array}{ccccc} \cdot & \cdot & 1 & \cdot & \cdot \\ \cdot & \cdot & \cdot & \cdot & \cdot \\ \cdot & \cdot & \cdot & \cdot & \cdot \\ \cdot & \cdot & \cdot & \cdot & \cdot \\ \cdot & \cdot & \cdot & \cdot & \cdot \end{array}\right| \end{array} & \begin{array}{c} +0{\langle e_{-1,1}\rangle }^{J=2}-\sqrt{\frac{1}{2}}{\langle e_{0,2}\rangle }^{J=2}\\ +0\left|\begin{array}{ccccc} \cdot & \cdot & \cdot & \cdot & \cdot \\ \cdot & \cdot & \cdot & 1 & \cdot \\ \cdot & \cdot & \cdot & \cdot & \cdot \\ \cdot & \cdot & \cdot & \cdot & \cdot \\ \cdot & \cdot & \cdot & \cdot & \cdot \end{array}\right|+\sqrt{\frac{1}{2}}\left|\begin{array}{ccccc} \cdot & \cdot & \cdot & \cdot & \cdot \\ \cdot & \cdot & \cdot & \cdot & \cdot \\ \cdot & \cdot & \cdot & \cdot & 1\\ \cdot & \cdot & \cdot & \cdot & \cdot \\ \cdot & \cdot & \cdot & \cdot & \cdot \end{array}\right| \end{array}\\ \begin{array}{c} {\langle v_{q=\pm 2}^{4}\rangle }^{J=2}=\sqrt{\frac{3}{14}}{\langle e_{-2,0}\rangle }^{J=2}\\ \left|\begin{array}{ccccc} \cdot & \cdot & \sqrt{\frac{3}{14}} & \cdot & \cdot \\ \cdot & \cdot & \cdot & -\sqrt{\frac{8}{14}} & \cdot \\ \cdot & \cdot & \cdot & \cdot & \sqrt{\frac{3}{14}}\\ \cdot & \cdot & \cdot & \cdot & \cdot \\ \cdot & \cdot & \cdot & \cdot & \cdot \end{array}\right|=\sqrt{\frac{3}{14}}\left|\begin{array}{ccccc} \cdot & \cdot & 1 & \cdot & \cdot \\ \cdot & \cdot & \cdot & \cdot & \cdot \\ \cdot & \cdot & \cdot & \cdot & \cdot \\ \cdot & \cdot & \cdot & \cdot & \cdot \\ \cdot & \cdot & \cdot & \cdot & \cdot \end{array}\right| \end{array} & \begin{array}{c} -\sqrt{\frac{8}{14}}{\langle e_{-1,1}\rangle }^{J=2}-\sqrt{\frac{3}{14}}{\langle e_{0,2}\rangle }^{J=2}\\ -\sqrt{\frac{8}{14}}\left|\begin{array}{ccccc} \cdot & \cdot & \cdot & \cdot & \cdot \\ \cdot & \cdot & \cdot & 1 & \cdot \\ \cdot & \cdot & \cdot & \cdot & \cdot \\ \cdot & \cdot & \cdot & \cdot & \cdot \\ \cdot & \cdot & \cdot & \cdot & \cdot \end{array}\right|+\sqrt{\frac{3}{14}}\left|\begin{array}{ccccc} \cdot & \cdot & \cdot & \cdot & \cdot \\ \cdot & \cdot & \cdot & \cdot & \cdot \\ \cdot & \cdot & \cdot & \cdot & 1\\ \cdot & \cdot & \cdot & \cdot & \cdot \\ \cdot & \cdot & \cdot & \cdot & \cdot \end{array}\right| \end{array} \end{array}$$

Tensor 〈**v***_q_^k^*〉*^J^* relations easily invert to 〈**e***_m_*_′_*_,m_*〉*^J^* by inspection due to their being orthonormal sets.

(16b) $$\begin{array}{cc} {\langle e_{-2,0}\rangle }^{J=2}=\sqrt{\frac{2}{7}}{\langle v_{q=\pm 2}^{2}\rangle }^{J=2} & +\sqrt{\frac{1}{2}}{\langle v_{q=\pm 2}^{3}\rangle }^{J=2}+\sqrt{\frac{3}{14}}{\langle v_{q=\pm 2}^{4}\rangle }^{J=2}\\ {\langle e_{-1,1}\rangle }^{J=2}=\sqrt{\frac{3}{7}}{\langle v_{q=\pm 2}^{2}\rangle }^{J=2} & +0{\langle v_{q=\pm 2}^{3}\rangle }^{J=2}-\sqrt{\frac{8}{14}}{\langle v_{q=\pm 2}^{4}\rangle }^{J=2}\\ {\langle e_{0,2}\rangle }^{J=2}=\sqrt{\frac{2}{7}}{\langle v_{q=\pm 2}^{2}\rangle }^{J=2} & -\sqrt{\frac{1}{2}}{\langle v_{q=\pm 2}^{3}\rangle }^{J=2}+\sqrt{\frac{3}{14}}{\langle v_{q=\pm 2}^{4}\rangle }^{J=2} \end{array}$$

Any [*J*]-by-[*J*] matrix is a combination of elementary 〈**e***_m_*_′_*_,m_*〉*^J^* and thus also of 〈**v***_q_^k^*〉*^J^*. This leads to RES maps that approximate [*J*]-by-[*J*] matrix 〈**v***_q_^k^* 〈*^J^* eigensolutions by plotting related combinations of *Y_q_^k^* (*θ, φ*) for select *θ_M_^J^*.

#### 1.3.3. Fano–Racah Tensor Algebra

Diagonal dipole-vector (rank *k* = 1) matrix 〈**v**_0_^1^〉*^J^* is seen in top row of Table 2 to be proportional to the angular momentum *z*-component matrix 〈**J***_z_*〉*^J^*. Diagonal 2*^k^*-pole (rank-*k*) tensors 〈**v**_0_*^k^*〉*^J^* are linearly related to **J***_z_* powers **J***_z_*^2^ = **J***_z_***J***_z_,***J***_z_*^3^ = **J***_z_***J***_z_***J***_z_, . . .* up to the *k^th^*-power **J***_z_^k^*. This relates 〈**v**_0_*^k^*〉*^J^* -eigenvalues to powers*m^p^* of 〈**J***_z_*〉-eigenvalues*m*and, in turn, leads to an RES scheme to analyze 〈**v***_q_^k^* 〉*^J^* eigensolutions.

For example, matrix diagonals in Table 2 give elementary representations for *J* = 2.

(17a) $$\begin{array}{ll} \sqrt{5}{\langle v_{0}^{0}\rangle }^{\left(J=2\right)}={\langle 1\rangle }^{\left(2\right)} & =\left(\begin{array}{ccccc} 1 & 1 & 1 & 1 & 1 \end{array}\right)\\ \sqrt{10}{\langle v_{0}^{1}\rangle }^{\left(J=2\right)}={\langle J_{z}\rangle }^{\left(2\right)} & =\left(\begin{array}{ccccc} 2 & 1 & 0 & -1 & 2 \end{array}\right) \end{array}$$

(17b) $$\begin{array}{ll} \sqrt{14}{\langle v_{0}^{2}\rangle }^{\left(2\right)} & =\left(\begin{array}{ccccc} 2 & -1 & -2 & -1 & 2 \end{array}\right)\\ \sqrt{10}{\langle v_{0}^{3}\rangle }^{\left(2\right)} & =\left(\begin{array}{ccccc} 1 & -2 & 0 & 2 & -1 \end{array}\right)\\ \sqrt{70}{\langle v_{0}^{4}\rangle }^{\left(2\right)} & =\left(\begin{array}{ccccc} 1 & -4 & 6 & -4 & 1 \end{array}\right) \end{array}$$

Powers of 〈**J***_z_*〉^2^ in Equation (18) are combinations of 〈**v***_q_^k^* 〉^2^ found by dot products with vectors in Equations (17a) and (17b).

(18) $$\begin{array}{llllll} {\langle J_{z}^{0}\rangle }^{\left(2\right)}=\left(\begin{array}{ccccc} 1 & 1 & 1 & 1 & 1 \end{array}\right)= & \frac{5}{\sqrt{5}}{\langle v_{0}^{0}\rangle }^{\left(2\right)} & & & & \\ {\langle J_{z}^{1}\rangle }^{\left(2\right)}=\left(\begin{array}{ccccc} 2 & 1 & 0 & -1 & -2 \end{array}\right)= & & \frac{10}{\sqrt{10}}{\langle v_{0}^{1}\rangle }^{\left(2\right)} & & & \\ {\langle J_{z}^{2}\rangle }^{\left(2\right)}=\left(\begin{array}{ccccc} 4 & 1 & 0 & 1 & 4 \end{array}\right)= & \frac{10}{\sqrt{5}}{\langle v_{0}^{0}\rangle }^{\left(2\right)} & & +\frac{14}{\sqrt{14}}{\langle v_{0}^{2}\rangle }^{\left(2\right)} & & \\ {\langle J_{z}^{3}\rangle }^{\left(2\right)}=\left(\begin{array}{ccccc} 8 & 1 & 0 & -1 & -8 \end{array}\right)= & & \frac{34}{10}{\langle v_{0}^{1}\rangle }^{\left(2\right)} & & +\frac{12}{\sqrt{10}}{\langle v_{0}^{3}\rangle }^{\left(2\right)} & \\ {\langle J_{z}^{4}\rangle }^{\left(2\right)}=\left(\begin{array}{ccccc} 16 & 1 & 0 & 1 & 16 \end{array}\right)= & \frac{34}{\sqrt{5}}{\langle v_{0}^{0}\rangle }^{\left(2\right)} & & +\frac{62}{\sqrt{14}}{\langle v_{0}^{2}\rangle }^{\left(2\right)} & & +\frac{24}{\sqrt{70}}{\langle v_{0}^{4}\rangle }^{\left(2\right)} \end{array}$$

(19) $$\begin{array}{lllll} {\langle v_{0}^{0}\rangle }^{\left(2\right)}=\frac{1}{\sqrt{5}}{\langle J_{z}^{0}\rangle }^{\left(2\right)} & & & & \\ {\langle v_{0}^{1}\rangle }^{\left(2\right)}= & \frac{1}{\sqrt{10}}{\langle J_{z}^{1}\rangle }^{\left(2\right)} & & & \\ {\langle v_{0}^{2}\rangle }^{\left(2\right)}=-\frac{2}{\sqrt{14}}{\langle J_{z}^{0}\rangle }^{\left(2\right)} & & +\frac{1}{\sqrt{14}}{\langle J_{z}^{2}\rangle }^{\left(2\right)} & & \\ {\langle v_{0}^{3}\rangle }^{\left(2\right)}= & -\frac{34\sqrt{10}}{120}{\langle J_{z}^{1}\rangle }^{\left(2\right)} & & +\frac{\sqrt{10}}{12}{\langle J_{z}^{3}\rangle }^{\left(2\right)} & \\ {\langle v_{0}^{4}\rangle }^{\left(2\right)}=\frac{3\sqrt{70}}{\left(5)(7\right)}{\langle J_{z}^{0}\rangle }^{\left(2\right)} & & -\frac{31\sqrt{70}}{\left(3)(7)(8\right)}{\langle J_{z}^{2}\rangle }^{\left(2\right)} & & +\frac{\sqrt{70}}{24}{\langle J_{z}^{4}\rangle }^{\left(2\right)} \end{array}$$

Triangle inversion of Equation (18) gives each 〈**v**_0_*^k^*〉^2^ in terms of **J***_z_* powers 〈**J***_z_^p^* 〉^2^ = *m^p^* in Equation (19). RES plots depend on relating 〈**v**_0_*^k^*〉*^J^* expansions Equation (19) in **J***_z_* to Wigner (*J,m*) polynomials ${\left(-1\right)}^{J-m}\sqrt{\left[k\right]}\left(\begin{array}{ccc} k & J & J\\ 0 & m & -m \end{array}\right)$ in Equation (14) and Legendre polynomials $D_{00}^{k}(\cdot \theta \cdot )=P_{k}(\text{cos }\theta )$ in Equation (11) that are also polynomials of **J***_z_* = |*J*| cos *θ*. By plotting the latter we hope to shed light on the eigensolutions of the former.

## 2. Tensor Eigensolution and Legendre Function RE Surfaces

Legendre polynomials occupy the central (00)-component of a Wigner-*D^J^* matrix.

(20) $$D_{00}^{k}(\cdot \theta \cdot )=P_{k}(\text{cos }\theta )$$

Examples of Legendre polynomials of cos *θ* = *J_z_/*|*J*| and *J_z_* = |*J*| cos *θ* are given below.

(21a) P0=1    P1(cos θ)=cos θ   P2(cos θ)=-12 32cos2 θ  P3(cos θ)=-32cos θ +52cos3 θ P4(cos θ)=38 -308cos2 θ +358cos4 θ

(21b) P0=1    ∣J∣1P1(cos θ)=Jz   ∣J∣2P2(cos θ)=-12∣J∣2 32Jz2  ∣J∣3P3(cos θ)=-32∣J∣2Jz +52Jz3 ∣J∣4P4(cosθ)=38∣J∣4 -308∣J∣2Jz2 +358Jz4

Classical *P_k_* functions are compared with corresponding quantized 〈**v**_0_*^k^*〉*^J^* unit-tensor *e*-values in Table 4 that generalize examples of tensor matrix (*J*=1 to 3)-eigenvalues in Table 2 and Equation (19) to any *J* and *m* = *J, . . . ,*− *J*. The powers of *m* and *J* in 〈**v**_0_*^k^*〉*^J^*, shown in Table 4 are taken to higher order in Table 5.

Norm $2^{k}\sqrt{\left[k\right]}/\sqrt{2J+k:-k+1}$ makes each 〈**v**_0_*^k^*〉*^J^* a unit vector. (Note: *A* + *a : b* = (*A* + *a*)(*A* + *a* − 1) . . . (*A* + *b*).) In contrast, normalized *P_k_* have *P_k_*(cos 0) = 1. Coefficients *c_p_* of cos*^p^θ* sum to 1 = ∑*c_p_*. Square |*c_p_*|^2^ usually do not sum to 1.

Tensor values 〈**v**_0_^0^〉*^J^*, 〈**v**_0_^1^〉*^J^*, and 〈**v**_0_^2^〉*^J^* in [. . . ]-braces of Table 4 equal Legendre functions *P*_0_, *P*_1_, and *P*_2_ in Equation (21b) exactly using *J*-expectation values Equations (22a) and (22b). However, for rank higher than *k* = 2, *P_k_* is only approximately equal to 〈**v**_0_*^k^*〉*_m_^J^* though the approximation improves with higher J.

(22a) $${\langle J_{z}\rangle }_{m}^{L}=m={\langle |J|\rangle }_{m}^{J}\text{cos }\theta_{m}^{J}$$

(22b) $$\langle |J_{z}|\rangle =\sqrt{J(J+1)}≅J+\frac{1}{2}$$

For large-*J* values, the 〈**v**_0_*^k^*〉*_m_^J^* in Table 4 approach the *P*_3_*, P*_4_*, . . .* of Equation (21b) according to the relation: ${\langle |J{|}^{k}\rangle }_{m}^{J}\vec{J\gg k}\;{\left[J(J+1)\right]}^{k/2}$. However, 〈**v**_0_*^k^*〉*_m_^J^* differ significantly from *P_k_* for low *J*. The classical *P_k_* in Equation (21b) lack the small terms (−2*/*3, −5*/*6, *etc*.) that kill the 〈**v**_0_*^k^*〉 in Table 4 whenever *J* falls below strict quantum limits such as whenever *J <* |*m*| or *J < k/*2. However, the quantum “killer” terms become negligible for larger J-values (*J > k*) and this makes tensor eigenvalues converge to *P_k_* and thus to their RES plots.

### 2.1. Angular Momentum Cones and RES Paths

Quantum *J*-magnitude Equation (22b) introduces a quantum angular momentum cone geometry with quantized angles *θ_m_^J^* given by Equation (22a) as summarized here in Equation (23a) and (23b) for lab *m* = *M* and molecular body *n* = *K*.

(23a) $$\text{cos }\theta_{M}^{J}=\frac{M}{\sqrt{J(J+1)}}$$

(23b) $$\text{cos }\theta_{K}^{J}=\frac{K}{\sqrt{J(J+1)}}$$

**Figure 1:**
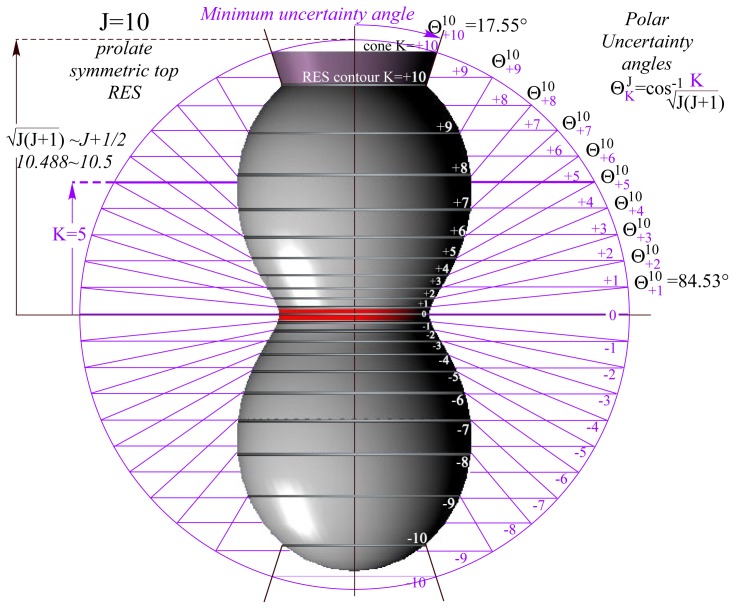
J = 10 Symmetric top RES. Angular momentum cone of minimum uncertainty angle *θ*_10_^10^=17.55° intersects the highest *K*=*J*=10 of the quantized **J**-path contour circles.

An angular momentum eigenstate $|\begin{array}{l} J\\ m,n \end{array}\rangle$ has sharp (zero-uncertainty) eigenvalue *m* or *n* on the lab or body frame *z* or *z̄* axis, respectively. This sharp altitude and magnitude in Equation (22b) constrains vector **J** to base circles of cones making half-angle *θ_m_^J^* or *θ_n_^J^* with *z* or *z̄* axes, respectively. Expected **J**-values appear in Figure 1 at intersections of quantized **J**-cones with the RES as explained below.

RES energy level analysis begins by writing a multipole *T_q_^k^* tensor expansion Equation (24a) of a general rigid rotor or asymmetric top Hamiltonian and then plotting the resulting surface using Equation (24a)

(24a) $$\begin{array}{l} H=A{\left(J_{\bar{x}}\right)}^{2}+B{\left(J_{\bar{y}}\right)}^{2}+C{\left(J_{\bar{z}}\right)}^{2}\\ =\frac{1}{3}(A+B+C)\;T_{0}^{0}+\frac{1}{3}(2C-A-B)\;T_{0}^{2}+\frac{1}{\sqrt{6}}(A-B)\;(T_{2}^{2}+T_{-2}^{2}) \end{array}$$

(24b) $$\begin{array}{l} T_{0}^{0}-{\left(J_{\bar{x}}\right)}^{2}+{\left(J_{\bar{y}}\right)}^{2}+{\left(J_{\bar{z}}\right)}^{2}=|J{|}^{2}\\ T_{0}^{2}=\frac{-1}{2}{\left(J_{\bar{x}}\right)}^{2}-\frac{1}{2}{\left(J_{\bar{y}}\right)}^{2}+{\left(J_{\bar{z}}\right)}^{2}=|J{|}^{2}\left(\frac{3}{2}{\text{cos}}^{2}\;\theta -\frac{1}{2}\right)\\ =|J{|}^{2}P_{2}(\text{cos }\theta )\\ T_{2}^{2}+T_{-2}^{2}=-\sqrt{\frac{3}{2}}{\left(J_{\bar{x}}\right)}^{2}+\sqrt{\frac{3}{2}}{\left(J_{\bar{y}}\right)}^{2}\\ =|J{|}^{2}\sqrt{\frac{3}{2}}{\text{sin}}^{2}\;\theta \;\text{cos }2\varphi \end{array}$$

Inertial constants (*A* = 1*/I_χ̄_, B* = 1*/I_ȳ_, C* = 1*/I_z̄_*) combine *J*-tensor operators *T_q_^k^*. Exact relation of 〈**v**_0_^0^〉*^J^* and 〈**v**_0_^2^〉*^J^* in Table 4 to classical *P*_0_ and *P*_2_ in Equation (21b) is used in Equation (24b) for *T*_0_^0^ and *T*_0_^2^.

A rigid spherical top (*A* = *B* = *C*) has only the *T*_0_^0^ term Equation (24a). Rigid prolate (*A* = *B > C*) or oblate (*A* = *B < C*) symmetric tops have only *T*_0_^0^ and *T*_0_^2^ terms with energy eigenvalues below.

(25) $$\begin{array}{r} \langle \begin{array}{l} J\\ m,n \end{array}|H^{\text{SymTop}}|\begin{array}{l} J\\ m,n \end{array}\rangle =\\ \langle \begin{array}{l} J\\ m,n \end{array}|B{\left(J_{\bar{x}}\right)}^{2}+B{\left(J_{\bar{y}}\right)}^{2}+C{\left(J_{\bar{z}}\right)}^{2}|\begin{array}{l} J\\ m,n \end{array}\rangle \\ =\frac{1}{3}\langle \begin{array}{l} J\\ m,n \end{array}|(2B+C)T_{0}^{0}+2(C-B)T_{0}^{2}|\begin{array}{l} J\\ m,n \end{array}\rangle \\ =\langle \begin{array}{l} J\\ m,n \end{array}|B|J{|}^{2}+(C-B){\left(J_{\bar{z}}\right)}^{2}|\begin{array}{l} J\\ m,n \end{array}\rangle \\ =BJ(J+1)+(C-B)n^{2} \end{array}$$

Since a rigid symmetric-top involves only *T*_0_^0^ and *T*_0_^2^, the *θ_n_^J^* -cones define its eigenvalues exactly by **J**-vector trajectories at angle-*θ_n_^J^* where *θ_n_^J^* -cones intersect the following *T*_0_^2^ -RES shown in Figure 1.

(26a) $$RE^{\text{SymTop}}(\theta )=\frac{1}{3}(2B+C)T_{0}^{0}(\theta )+\frac{2}{3}(C-B)T_{0}^{2}(\theta )$$

Inserting quantized-body cone relation Equation (23b) yields desired eigenvalues Equation (25) exactly.

(26b) $$\begin{array}{r} RE^{\text{SymTop}}(\theta_{L}^{J})=\frac{1}{3}(2B+C)J(J+1)\\ +\frac{2}{3}(C-B)J(J+1)\;\left(\frac{3}{2}{\text{cos}}^{2}\;\theta_{K}^{J}-\frac{1}{2}\right)\\ =J(J+1)\frac{1}{3}[(2B+C)+(C-B)(K^{2}-1)]\\ =J(J+1)B+(C-B)K^{2} \end{array}$$

Cone paths in Figure 1 are constant energy contours on symmetric top RES Equations (26a) and (26b) of constant *J*. They may be viewed as *J*-phase paths on which the **J**-vector may delocalize or “precess” on a circular *θ_n_^J^* -cone around body *z̄*-axis. Or else one might view paths on Figure 1 as coordinate space tracks of the lab *z*-axis around the *z̄*-axis by assuming **J** lies fixed on the former. Either view describes **J** in the body-frame by Euler polar and azimuth angles −*β,*−γ with angle *β* = *θ_n_^J^* and |*J*|^2^ = *J*(*J* + 1) fixed.

(27) $$J=(J_{\bar{x}},J_{\bar{y}},J_{\bar{z}})=(-|J|\text{cos}\;\gamma \;\text{sin }\beta ,|J|\text{sin }\gamma \;\text{sin }\beta ,|J|\text{cos }\beta )$$

**Figure 2:**
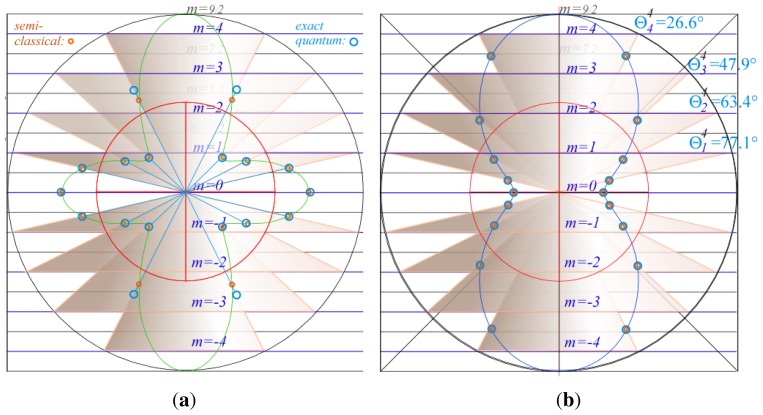
Quantum eigenvalues (blue) compared with semi-classical cone values (orange) for multipole tensor rank (**a**) *k* = 4 (approximate), 〈**v**_0_^4^〉*_m_*^4^ and *P*_4_(cosΘ *_m_*^4^) diverge for large *m*; and (**b**) *k* = 2 (exact), 〈**v**_0_^2^〉*_m_*^4^ and *P*_2_(cosΘ*_m_*^4^) correspond for all *m*.

The difference between quantum solutions and semi-classical *P_k_* solutions can be easily plotted as in Figure 2. The figure shows a slice of the semi-classical surface and the uncertainty cones for each *m* from *J* to −*J*. The orange circles indicate the intersection of the surface with the uncertainty cones and the blue circles indicate the energy of the quantum value, 〈**v**_0_*^k^*〉*_m_^J^*, placed along the uncertainty cone. Figure 2(a) shows some divergence between quantum and semi-classical energies for 〈**v**_0_^4^〉*_m_*^4^, while Figure 2(b) are exact for all *J* as in the *J* = 4 cases 〈**v**_0_^2^〉*_m_*^4^ shown. As *J* and *m* are made larger than *k* then semi-classical values converge on exact eigenvalues as described below.

#### 2.1.1. Reduced Matrix and RES Scaling

An RES is a radial plot along **J** direction −*β,* −γ that has hills where energy is high and valleys where it is low, but at all points the same magnitude $|J|=\sqrt{J(J+1)}$. Origin-shift to keep RES radius positive and scaling to display hills, valleys, and saddles, may be needed to make useful RES plots.

A scalar term *s* · **v**_0_^0^ added to a tensor combination **T** = **∑***_k_t_k_***v**_0_*^k^* does not affect the **T**-eigen*vectors* and neither does an overall scaling of **T** to c**T**. This is true since eigenvectors are invariant to adding a multiple *s***1** of unit matrix **1** to **T** or multiplying it by *c***1**. (Of course, eigen*values* would, respectively, be shifted by *s* or scaled by *c*.)

Wigner–Racah tensor algebra defines a reduced matrix element 〈*J*′||*T^k^*||*J*〉 to serve as a scale factor for each Clebsch–Gordan tensor matrix element having Wigner–Eckart form Equation (13a).

(28) $$\langle \begin{array}{c} J^{\prime }\\ m^{\prime } \end{array}|T_{q}^{k}|\begin{array}{c} J\\ m \end{array}\rangle =C_{qmm^{\prime }}^{kJJ^{\prime }}\langle J^{\prime }||T^{k}||J\rangle$$

This matrix $\langle \begin{array}{c} J\\ m \end{array}|T_{0}^{2}|\begin{array}{c} J\\ m \end{array}\rangle$ of quadrupole-*J*-tensor *T*_0_^2^ = **J**_0_^2^ in Equation (24b) reveals some key points.

(29a) $$\begin{array}{l} \langle \begin{array}{c} J\\ m \end{array}|\left(\frac{3}{2}J_{z}^{2}-\frac{1}{2}J^{2}\right)|\begin{array}{c} J\\ m \end{array}\rangle =\langle \begin{array}{c} J\\ m \end{array}|T_{0}^{2}|\begin{array}{c} J\\ m \end{array}\rangle \\ =(C_{0mm}^{2JJ})\cdot \langle J||T^{2}||J\rangle \end{array}$$

(29b) $$\begin{array}{l} \frac{3}{2}m^{2}-\frac{1}{2}J(J+1)=\langle \begin{array}{c} J\\ m \end{array}|T_{0}^{2}|\begin{array}{c} J\\ m \end{array}\rangle \\ =\frac{4[J]}{\sqrt{2J+3:-1}}\left(\frac{3}{2}m^{2}-\frac{1}{2}J(J+1)\right)\cdot \frac{\sqrt{2J+3:-1}}{4[J]} \end{array}$$

Reduced matrix element 〈*J* ||*T*^2^|| *J*〉 cancels norm factor $4\sqrt{\left[J\right]}/\sqrt{2J+3:-1}$ in *C*_0_*_mm_*^2^*^JJ^*. The result is the quadratic Legendre form |*J*|^2^*P*_2_(*m/*|*J*|) found inside [. . . ]-braces of Table 4 with norm $4\sqrt{\left[k\right]}/\sqrt{2J+3:-1}$ outside the braces. (The latter is just a norm in Equation (29a) and (29b) multiplied by the factor $\sqrt{\left[k]/[J\right]}$ from definition Equation (15b).)

Apparent conflicts in factors are due to having sum-of-*squared*-component normalization of unit **v***^k^* on one hand and sum-of-component normalization of *P_k_* on the other. Matrix elements $\langle \begin{array}{c} J\\ m \end{array}|T|\begin{array}{c} J\\ m \end{array}\rangle$ or $|\begin{array}{c} J\\ m \end{array}\rangle \;\langle \begin{array}{c} J\\ m \end{array}|$ use the former since amplitude squares give probability. However, it is *un*squared amplitude sum ∑*c_k_* that measures anisotropy of a tensor *T* = ∑*c_k_* · *P_k_* since ∑*c_k_* is a maximum *T*-amplitude. (Each *P_k_* contribues *P_k_*(0) = 1.) Expectation values $\langle \begin{array}{c} J\\ m \end{array}|T|\begin{array}{c} J\\ m \end{array}\rangle$ scale linearly, too, but **J**^2^ tensors may have extra scale factors.

Tensor *T*^2^ = **J**^2^ in Equation (29a) and (29b) scales as |*J*|^2^ = *J*(*J* + 1) and *T^k^* = **J***^k^* scale as |*J*|*^k^*. Factor $4\sqrt{\left[J\right]}/\sqrt{2J+3:-1}$ of *C*_0_*_mm_*^2^*^JJ^* reduces scale |*J*|^2^ to $\sqrt{2J+1}=\sqrt{\left[J\right]}$. Then the reduced factor 〈*J* ||*T*|| *J*〉 brings it back to |*J*|^2^.

(30) $$\begin{array}{r} \frac{4[J]}{\sqrt{2J+3:-1}}=\\ \frac{4(2J+1)}{\sqrt{\left(2J+3)(2J+2)(2J+1)(2J)(2J-1\right)}}\\ =\frac{\sqrt{\left[J\right]}}{\sqrt{\left(J+\frac{3}{2})(J+1)J(J-\frac{1}{2}\right)}}\approx \frac{\sqrt{\left[J\right]}}{|J{|}^{2}} \end{array}$$

Each rank-*k* part has a factor |*J*|*^k^* = |*J*(*J* + 1)|*^k/^*^2^. Anisotropy of mixed-rank **J**-tensor **T** = ∑*c_k_* · **J***^k^* is ∑|*J*|*^k^c_k_*, and thus is quite sensitive to quantum number *J*. So also are the RES and related eigensolutions of **T**.

### 2.2. Asymmetric Top and Rank-2 RES

Plotting RES of non-diagonal Hamiltonians for the asymmetric top Equation (24a) involves 2*^nd^*-rank tensors **v***_q_*^2^ with reduced z-axial symmetry, nonzero *q*-values, and non-commuting *J_a_* combinations. Each *J_a_* in the quadratic expressions in Equation (24a) is replaced by its classical Euler-angle form in Equation (27).

Or else, each tensor *T_q_^k^* in Equation (24a) is replaced by a multipole fucntion $X_{q}^{k}=|J{|}^{k}D_{0q}^{k*}(.,\beta ,\gamma )$. (Recall Equation (24b)).

(31a) $$\begin{array}{r} H^{\text{AsymTop}}=A\;{\left(J_{\bar{x}}\right)}^{2}+B\;{\left(J_{\bar{y}}\right)}^{2}+C\;{\left(J_{\bar{z}}\right)}^{2}\\ \Rightarrow RES^{\text{AsymTop}}=A\;{\left(|J|\text{cos }\gamma \;\text{sin }\beta \right)}^{2}+B\;{\left(|J|\text{sin }\gamma \;\text{sin }\beta \right)}^{2}+C\;{\left(|J|\text{cos }\beta \right)}^{2} \end{array}$$

(31b) $$\begin{array}{l} H^{\text{AsymTop}}=\frac{A+B+C}{3}T_{0}^{0}+\frac{2C-A-B}{3}T_{0}^{2}+\frac{A-B}{\sqrt{6}}(T_{2}^{2}+T_{-2}^{2})\\ \Rightarrow RES^{\text{AsymTop}}=\frac{A+B+C}{3}|J{|}^{2}+\frac{2C-A-B}{3}X_{0}^{2}+\frac{A-B}{\sqrt{6}}(X_{2}^{2}+X_{-2}^{2})\\ =|J{|}^{2}\left[\frac{A+B}{2}+\frac{2C-A-B}{2}{\text{cos}}^{2}\;\beta +\frac{A-B}{2}{\text{sin}}^{2}\;\beta \;\text{cos }2\gamma \right] \end{array}$$

**Figure 3:**
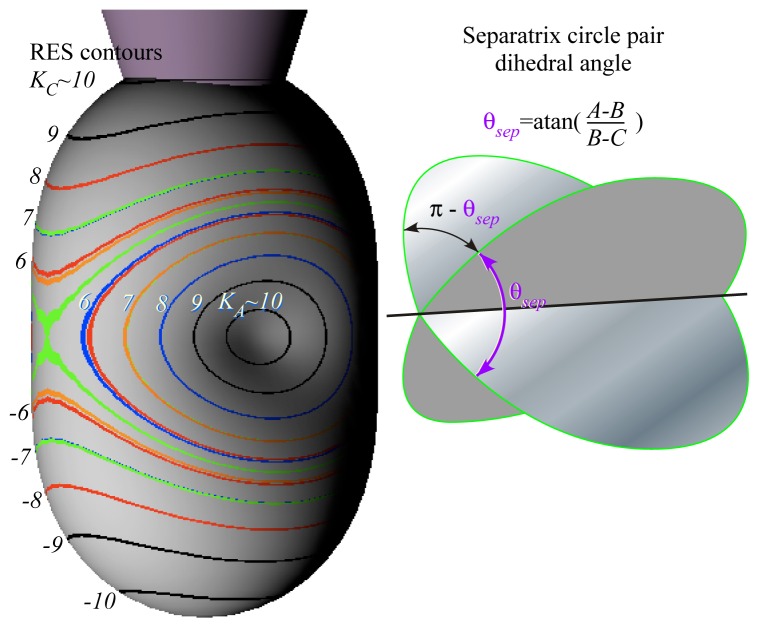
Asymmetric top RES *J* = 10.

Forms Equations (31a) and (31b) of rank-(*k* = 2)-RES are equal and give the same plots shown in Figure 3, but tensor form Equation (31b) reveals symmetry. Terms *X*_0_^0^ and *X*_0_^2^ (Figure 1) are *z*-symmetric and non-zero near *z*-axis while *X*_±2_^2^ terms are asymmetric and vary as sin^2^*β* with polar angle *β* between the **J** vector and the *z*-axis. As *β* approaches *π/*2, *X*_±2_^2^ terms grow to give equatorial valleys and saddles in Figure 3 while *X*_0_^2^ vanishes.

**Figure 4:**
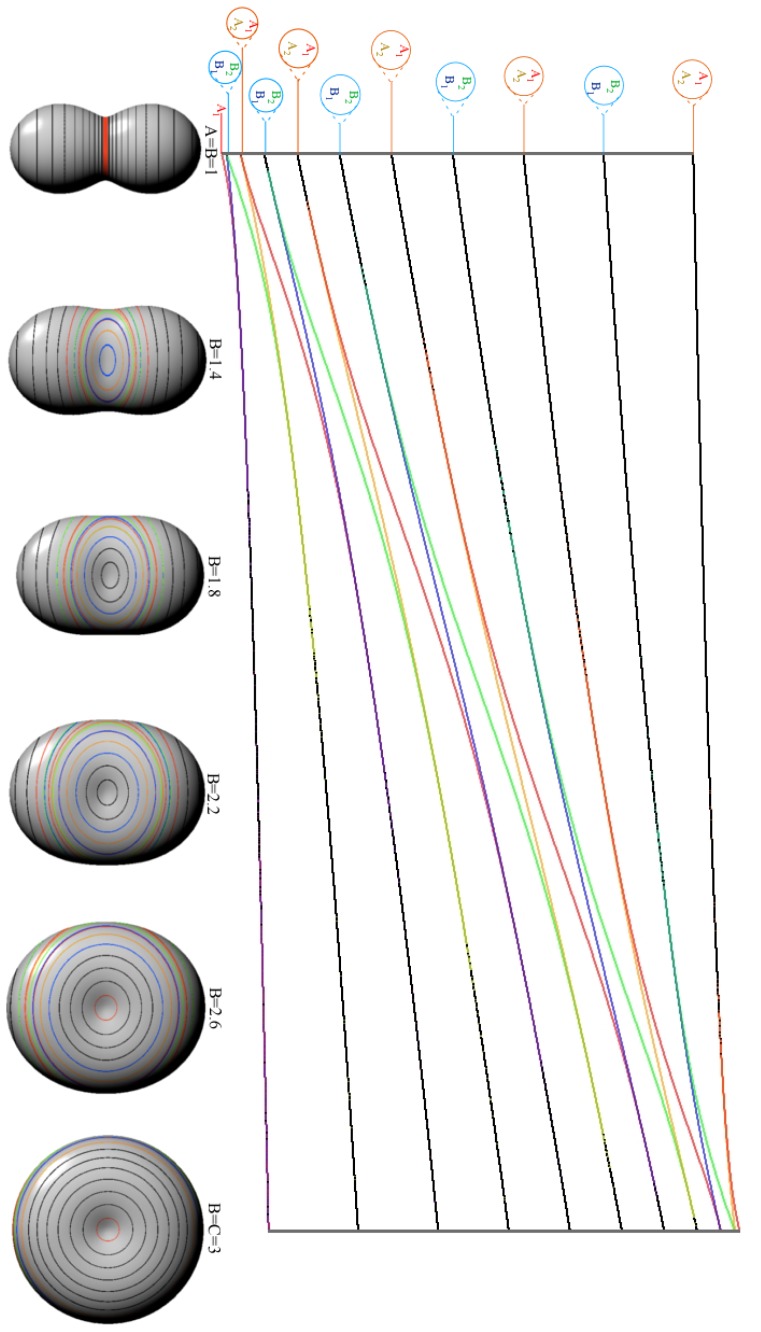
Asymmetric top energy levels with corresponding RES.

Asymmetric tensor operators *T*_±_*_q_^k^* are non-diagonal and do not commute with diagonal *T*_0_*^k^* or with each other, and so *H*^AsymTop^ eigenstates as well as eigenvalues vary with coefficient (*A* − *B*) in Equation (31b). As *T*_±2_^2^ mixes symmetric-top states $|\begin{array}{c} J\\ K \end{array}\rangle$ into asymmetric-top eigenstates, *θ_K_^J^* cone circles around the *z*-axis of Figure 1 warp into oval-pairs squeezed by nascent ovals emerging from the *x*-axis and bound by a pair of separatrix circle-planes that meet at an angle *θ*^sep^ on the *y*(or*B*)-axis. Figure 4 shows a range of RES and levels between symmetric-prolate top (*B* = *A* or *θ*^sep^ = 0) and oblate top (*B* = *C* or *θ*^sep^ = *π*). A most-asymmetric case (*B* = *C* or *θ*^sep^ = *π/*2) is midway between the symmetric cases.

(32) $$\theta^{\text{sep}}={\text{tan}}^{-1}\frac{|A-B|}{|B-C|}=\{\begin{array}{ll} 0 & \text{for}:B=A\\ \pi /2 & \text{for}:B=(A+C)/2\\ \pi & \text{for}:B=C \end{array}$$

As B first differs a little from A, off-diagonal *T*_±2_^2^ and asymmetric *X*_±2_^2^ first “quench” degenerate ±*K*-momentum eigenstate pairs $|\begin{array}{c} J\\ \pm K \end{array}\rangle$ into non-degenerate standing cos or sin-wave pairs.

(33a) $$|c_{K}^{J}\rangle =\frac{1}{\sqrt{2}}\left(|\begin{array}{c} J\\ +K \end{array}\rangle +|\begin{array}{c} J\\ -K \end{array}\rangle \right)$$

(33b) $$|s_{K}^{J}\rangle =\frac{-i}{\sqrt{2}}\left(|\begin{array}{c} J\\ +K \end{array}\rangle -|\begin{array}{c} J\\ -K \end{array}\rangle \right)$$

These states have nodes or anti-nodes standing on hills, saddles, or valleys of the RES topography at the principal body axes. Whether a wave is cos-like or sin-like at an axial point depends on whether it is symmetric or antisymmetric at the point and thus whether that point is an anti-node or node. Nodal location can determine whether a cos-like or sin-like wave has higher energy.

As *B* differs more and more from *A*, off-diagonal *T*_±2_^2^ will mix standing waves like *|c_K_^J^*〉 with others such as|*c_K_*_±2_*^J^*〉, |*c_K_*_±4_*^J^*〉, and |*c_K_*_±6_*^J^*〉 that share the same *H*^AsymTop^ symmetry described below.

### 2.3. Symmetry Labeling of Asymmetric Top Eigenstates

Throughout the range of asymmetric cases in Figure 4 the symmetry of *H*^A Top^ in Equation (31a) and (31b) is at least orthorhombic group *D*_2_ of 180° rotations **R***_x_*, **R***_y_*, and **R***_z_* about inertial body axes that mutually commute (**R***_x_***R***_y_* = **R***_z_* = **R***_y_***R***_x_*), *etc.*). Unit square (**R***_x_*^2^ = **1**, *etc.*) **R**-eigenvalues ±1 label nodal symmetry (+1) or antisymmetry (−1) on each axis. *D*_2_ is an outer product of cyclic *C*_2_ groups for two axes, say *C*_2_(*x*) and *C*_2_(*y*). *x* and *y* values also label nodal symmetry for the *z* axis since **R***_x_***R***_y_* = **R***_z_*. A Cartesian 2-by-2 product of *C*_2_(*x*) and *C*_2_(*y*) symmetry character tables shown in Table 6 gives four sets of characters and four symmetry labels [*A*_1_*,B*_1_*,A*_2_*,B*_2_] for *D*_2_ = *C*_2_(*x*) ⊗ *C*_2_(*y*).

Labels (*A,B*) or (1*,* 2) for (*x*) or (*y*) symmetric and anti-symmetric states follow ancient arcane conventions. We prefer a binary (0_2_*,* 1_2_) notation for *C*_2_ characters and N-ary notation (0*_N_,* 1*_N_,* 2*_N_, . . . ,* (*N*−1)*_N_*) for *C_N_* characters $D^{m_{N}}(R^{p})$ where each label *m_N_* denotes “*m*-wave-quanta-modulo N” as in Table 7.

(34) $$D^{m_{N}}(R^{p})=e^{-im\cdot p(2\pi /N)}$$

This notation is used in correlation Table 8 between symmetry labels of *D*_2_ and its subgroups *C*_2_(*x*), *C*_2_(*y*), and *C*_2_(*z*), respectively. Each row belongs to a *D*_2_ species and indicates which *C*_2_ symmetry, *even* (0_2_) or *odd* (1_2_), correlates to it. The Table 8(a), 8(b) and 8(c) follow respectively from the columns **R***_x_*, **R***_y_*, and **R***_z_* of Table 6. An *even* (0_2_) *D*_2_ character is $D^{0_{2}}(R)=+1$ and *odd* (1_2_) is $D^{1_{2}}(R)=-1$.

*J* = 10 *H*^AsymTop^-levels in Figure 3 consist of two sets of five pairs [(*A*_1_*,B*_1_) (*A*_2_*,B*_2_) (*A*_1_*,B*_1_) (*A*_2_*,B*_2_) (*A*_1_*,B*_1_)] and [(*B*_2_*,A*_1_) (*B*_1_*,A*_2_) (*B*_2_*,A*_1_) (*B*_1_*,A*_2_) (*B*_2_*,A*_1_)] separated by a single (*A*_2_) level. Each is related to RES *x*-valley path pairs *K_x_* ~ [±10*,*±9*,*±8*,*±7*,*±6] or *z*-hill pairs *K_z_* ~ [±6, ±7, ±8, ±9, ±10] separated by a single *y*-path (*A*_2_: *K_y_* ~ 5). Even-*K* belongs to a (0_2_) column and odd-*K* belongs to a (1_2_) column of *C*_2_(*x*) Table 8(a) or *C*_2_(*z*) Table 8(c).

Valley-pair sequence (*A*_1_,*B*_1_), (*A*_2_,*B*_2_) . . . is consistent with (0_2_) and (1_2_) columns of the *C*_2_(*x*) Table 8(a), and hill-pair sequence (*B*_2_,*A*_1_), (*B*_1_,*A*_2_) . . . is consistent with (0_2_) and (1_2_) column of the *C*_2_(*z*) Table 8(c). This is because lower pairs correspond to *x*-axial RES loops of approximate momentum *K_x_* ~ ±10, ±9 · · · ± 6 while upper pairs correspond to *z*-axial RES loops of approximate momentum *K_z_* ~ ±6,±7 · · · ± 10 in Figure 3

Table 8(b) for *C*_2_(*y*) is not used since ±*y*-axes are hyperbolic saddle points on one separatrix path, unlike the disconnected *pairs* of elliptic RES paths that encircle ±*x*-axes or ±*z*-axes at hill or valley points. Only a single *E* level exists in Figure 3 at the energy *E*^Sep^ of the saddle points and their separatrix.

(35) $$E^{\text{Sep}}=H^{\text{AsymTop}}(J_{x},J_{y},J_{z})=BJ(J+1)\text{for}\{\begin{array}{ll} J_{x} & =0\\ J_{y} & =|J{|}^{2}\\ J_{z} & =0 \end{array}$$

As symmetric*H*^Sym^ becomes a more asymmetric*H*^AsymTop^ in Figure 4, a hill or valley path bends away from its ideal single-*K* symmetric-top cone circle at constant polar angle *θ_K_^J^* Equations (23a) and (23b). Each *H*^Sym^ state $|\begin{array}{c} J\\ K \end{array}\rangle$ turns into an *H*^AsymTop^ eigenstate expansion of states $|\begin{array}{c} J\\ K\pm 2p \end{array}\rangle$ with *K* ± 2*p* above and below *K*, and its RES path bends from constant *θ_K_^J^* toward polar angles *θ_K_*_±2_*^J^*, *θ_K_*_±4_*^J^*, *θ_K_*_±6_*^J^*. . . above and below angle *θ_K_^J^*. At energy near the separatrix *E*^Sep^, bending of hill and valley paths become more severe as they approach separatrix asymptotes where the polar angle range Equation (32) expands to 2*θ*^Sep^ or *π* and the bend becomes a kink.

It is conventional to label *H*^Sep^ eigenstate |*E*〉 by both *K_x_* and *K_z_* quantum values since |*E*〉 may use either a *K_x_* basis or else a *K_z_* basis. However, **J***_x_* and **J***_z_* do not commute. For energy *E* above *E*^Sep^, a |*J*,*K_z_*〉 expansion is more compact and a dominate |*K_z_*| value may label |*E*〉. For *E* below *E*^Sep^, a |*J*,*K_x_*〉 expansion has a meaningful |*K_x_*| label. For *E* near *E*^Sep^, *K*-labels are questionable.

Though a general form of the symmetry identification process may be unfamiliar, it may implemented by computer. Group projectors Equation (36) can distinguish how each eigenvector splits with respect to subgroup operations. The product of these projectors and the calculated eigenvectors identifies the subgroup symmetry of each level.

(36) $$P_{jk}^{\alpha }=\left(\frac{l^{\alpha }}{{}^{\circ}G}\right)\sum_{g}D_{jk}^{\alpha^{*}}(g)g$$

Only projectors in lower symmetry subgroups are used because they are easy to calculate and there are fewer in number. With the eigenvector projection lengths and knowledge of the correlation table between the molecular group itself and the subgroup one can start to deduce the eigenvector symmetry. As mentioned earlier, one correlation table is not enough to fully identify an eigenvector’s symmetry, but using several subgroups one can assign symmetry. This process is simpler than calculating projectors of the full group, particularly if one can use a *C_n_* subgroup and $D_{jk}^{\alpha^{*}}(g)$ in Equation (36) is found by Equation (34).

This method can be significantly simpler than a traditional block diagonalization. Block diagonlalizing the Hamiltonian requires projectors of the entire molecular symmetry group rather than of the smaller subgroups.

The disadvantage of this method is that it becomes unstable when clusters are tightest. As eigenvectors become more mixed with tighter clustering the algorithm may be unable to distinguish. Some RES paths and level curves indistinguishable to numeric projector then appear black. Symmetry definitions hold for asymmetric tops where *J <* 50. Spherical tops are quite challenging as seen in Section 7.

### 2.4. Tunneling between RES-Path States

N-atom inversion in ammonia, *NH*_3_, is an example of molecular tunneling modeled by a particle whose closely paired levels (inversion doublets) lie below the barrier of a double-well PES. An RES generalization, sketched in Figure 3, shows level pairs such as (*A*_1_,*B*_1_), (*A*_2_,*B*_2_), *etc.* as rotational analogs of inversion doublets. Here the tunneling between left and right positions on a PEs is replaced by an RES inversion between left-handed and right-handed rotation of an entire molecule. Instead of oscillation of expected position values 〈**r**〉 between PES valleys there is oscillation of momentum 〈**J**〉 between pairs of *x*-paths (+*K_x_* ↔ −*K_x_*) in RES valleys or else between pairs of *z*-paths (+*K_z_* ↔ −*K_z_*) on RES hills. Section 7 describes this phenomenon in more detail for molecules of *O_h_* and *T_d_* symmetry.

## 3. Tensor Eigensolutions for Octahedral Molecules

Section 2 has shown that asymmetric top molecules may be treated semi-classically, using only tensor operators and RES plots with a seperatrix between regions of local symmetry. Spherical top molecules experience such symmetry locality, but with greater variety of local symmetry. This section focuses on the added complication and convenience of higher symmetry as well as showing novel rotational level clustering patterns related to RES paths and tunneling.

### 3.1. Tensor Symmetry Considerations

Theory of asymmetric top spectra in Section 2 may be generalized to a semi-classical treatment of tensor operators for *T_d_* or *O_h_* symmetric molecules such as *CH*_4_ or *SF*_6_. The results contain level clusterings that first appeared in computer studies by Lea, Leask, andWolf [20], Dorney and Watson [21] and followed by symmetry analyses [22,23] and others described below.

Up to fourth order, any such molecule may be treated using the Hecht Hamiltonian [24] rewritten in terms of tensor operators below in Equation (37a) and (37b) that isolates the rank-4 tensor term Equation (37c).

(37a) $$H=BJ^{2}+10t_{004}{\left(J_{x}^{4}+J_{y}^{4}+J_{z}^{4}-\frac{3}{5}J\right)}^{4}$$

(37b) $$H=BT_{0}^{0}+4t_{044}\left[T_{0}^{4}+\sqrt{\frac{5}{14}}(T_{4}^{4}+T_{4}^{4})\right]$$

(37c) $$T^{\left[4\right]}=\sqrt{\frac{7}{12}}\left[T_{0}^{4}+\sqrt{\frac{5}{14}}(T_{4}^{4}+T_{4}^{4})\right]$$

This is continued below to higher rank tensors with more complicated structure [14]. The coefficients of each tensor operator may be found from a spherical harmonic addition-theorem expansion of points at vertices of an octahedron. Coefficients *c_n_*,*_m_* are based on Equation (38), where *Y_m_^n^* is the spherical harmonic and *f*(*⇉*) is a position of octahehral vertices (100), (010), ..., (00 − 1).

(38) $$c_{n,m}=\int_{V}f(\vec{r})Y_{m}^{n}\cdot r^{n}d\tau$$

A normalized sum of these coefficients gives the rank-6 *O_h_* tensor as follows.

(39) $$T^{\left[6\right]}=\frac{1}{2\sqrt{2}}T_{0}^{6}-\frac{\sqrt{7}}{4}\left(T_{4}^{6}+T_{-4}^{6}\right)$$

The first study of RES and eigenvalue spectrum with varying rank-4 and rank-6 tensor operators [25] expressed in Equation (40) revealed intricate level cluster crossing shown below.

(40) $$T^{\left[4,6\right]}=\text{cos}(\theta )T^{\left[4\right]}+\text{sin}(\theta )T^{\left[6\right]}$$

Later studies [26] examined rank-8 contributions such as expressed by Equation (41). Effects peculiar to combining Hamiltonian terms of rank-8 and higher include extreme clusters.

(41) $$T^{\left[4,6,8\right]}=\text{cos}(\varphi )\;\left(\text{cos}(\theta )T^{\left[4\right]}+\text{sin}(\theta )T^{\left[6\right]}\right)+\text{sin}(\varphi )T^{\left[8\right]}$$

As with the asymmetric top Hamiltonian, the octahedral Hamiltonian uses non-axial operators shown in Equations (37b) and (39). Such operators involve more than Legendre functions, complicating purely semi-classical analysis. Thus, approximate solutions based on axial operators alone apply only asymptotically for high *J >* 10 and in regions away from RES seperatrices. Combined powers of *J* and *J_z_* do not give all levels. Tensor operators provide a sparse banded Hamiltonian matrix, but full numerical diagonalization is needed to get all levels to high precision.

Gulacsi and coworkers [26] explored how eigenvalues vary with *T*^[4]^ and *T*^[8]^ for *J* ≤ 10 and small contributions of *T*^[6]^. Results below agree but extend to larger *J* and use RES topology.

While the asymmetric top systems show clustering related to symmetry subduction from *D*_2_ to a related *C*_2_ subgroup, octahedral molecular clusters relate to a variety of subgroups. *O_h_* may cluster into *D*_4_, *D*_3_, *D*_2_, *C*_4_, *C*_3_, *C*_2_ or other subgroups involving reflection or inversion.

For simplicity, this discussion will focus on *O* rather than *O_h_* molecules to make equations and correlation tables easier to display and interpret. Later Sections 6–7 give fuller discussions and explain the reciprocity relations that are behind correlation tables.

For *D*_2_-symmetric molecules, clustering patterns are described in terms of the correlation tables found in Table 8. The similar correlation tables for octahedral molecules are given for *C*_4_, *C*_3_ and *C*_2_ in Table 9. The columns of Table 9 represent the different clusters of rotational levels found within the spectra of given molecule at a given rotational transition. These clusterings are identified by their degeneracy as well as their RES location. Since symmetry labeling of octahedral group *O* differs form asymmetric top *D*_2_, a new coloring convention for *O* levels is defined: *A*_1_ is red, *A*_2_ is orange, *E*_2_ is green, *T*_1_ is dark blue and *T*_2_ is light blue.

**Figure 5:**
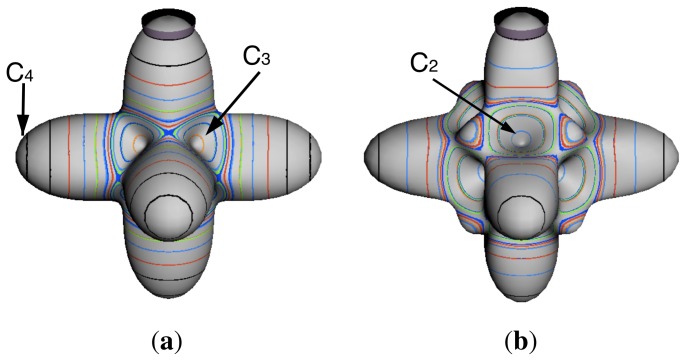
Symmetry axes of *T*^[4,6]^ RES for differing contributions of *T*^[4]^ and *T*^[6]^. (**a**) *C*_3_ and *C*_4_ local regions; (**b**) *C*_2_ local region.

In the RES, rotationally induced deformation or symmetry breaking is seen from the shape of local regions of the RES involving a specific contour. Figure 5 shows two different RES plots, both globally octahedral, but with local regions that correspond to a subgroup symmetry of the octahedron. Figure 5(a) demonstrates a possible RES of an octahedral molecule with Hamiltonian parameters that allow for *C*_4_ and *C*_3_ local symmetry regions to be present. The *C*_4_ regions are identified by their location and by their square base. Similarly, the *C*_3_ regions are identified by their location and triangular base. In this case *C*_3_ symmetric regions are concave while *C*_4_ regions are convex. This is not required and is dependent on Hamiltonian fitting terms that change the relative contributions of *T*^[4]^ and *T*^[6]^. Likewise, Figure 5(b) shows the *C*_2_ regions that are determined by their location and rectangular base.

Cluster degeneracy is a hallmark of a specific symmetry breaking. While a symmetric top spectra may be resolved into *m_J_* levels, a rotationally-induced symmetry-reduced spherical top has several identical *z* axes. The *m_J_* levels can then localize on a single symmetry-reduced local region. The number of these regions must equal the degeneracy of the cluster in that same region. This degeneracy, *ℓ^α^*, is also found using the sum of the numbers in the columns of Table 9 or by Equation (42) given °G is the order of the molecular symmetry group and ∘ℋ is the order of the subgroup.

(42) $$\ell^{\alpha }=\frac{{}^{\circ}G}{{}^{\circ}H}$$

In the cases shown here cluster degeneracy *ℒ ^α^* becomes 6, 8 and 12 for *C*_4_, *C*_3_ and *C*_2_ respectively.

### 3.2. Numerical Assignment of Symmetry Clusters

As mentioned previously, it is possible to diagonalize the Hamiltonian and organize species by the order of each block, yet this alone will not distinguish all levels. For Hamiltonians defined by *T*^[4]^ as Equation (37a) it is possible to analytically [25] determine the symmetry of each level. Once *T*^[6]^ or *T*^[8]^ terms are present, a numerical examination of eigenvectors is required to assign the symmetry of each level. Subgroup projectors are used here where the cluster degeneracy increases and the symmetry becomes challenging to distinguish. These projectors represent a simplification of the symmetry analysis of an octahedral molecule into projections onto *C*_4_ symmetric projectors. The correlation table for *O* ⊃ *C*_4_, shown in Table 9, and Equation (36) give the information necessary for the assignment. Moreover, when using the subgroup *C*_4_ there are only four projectors to create and a clever choice of axis can force several of these projectors to be entirely real or entirely imaginary. Conveniently, the *C*_4_ projectors can be used to diagnose level symmetry for clusters in any subgroup region.

### 3.3. Octahedral Clustering vs. RES Topography

#### 3.3.1. Variation of *T*^[4,6]^ Topography

The Hecht Hamiltonian Equation (37a) and its higher order analogues are generic Hamiltonians. Such Hamiltonians have numerous fitting constants specific to a given molecule and a given vibronic species. To better understand all such octahedral systems, one must focus on changes in the level spectrum and RES plots with varying contributions of *T*^[4]^, *T*^[6]^ and *T*^[8]^.

**Figure 6:**
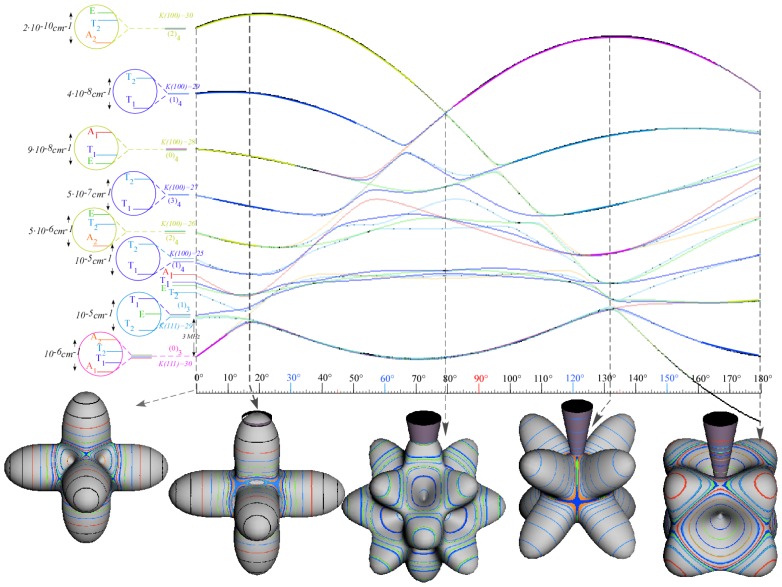
*J*=30 Energy levels and RES plots for *T*^[4,6]^*vs.* [4,6] mix-angle *φ* with *T*^[4]^ levels above *θ*=0° (extreme left), *T*^[6]^ levels at *θ*=90° (center), and −*T*^[4]^ levels at *θ*=180° (extreme right). *C*_4_ local symmetry and 6-fold level clusters dominate at *θ*=17°while *C*_3_ type 8-fold level clusters dominate at *θ*=132°. In between these extremes are *C*_2_ type 12-fold level clusters particularly around *θ*=80° where a *C*_3_ − *C*_4_ level-cluster-crossing of the top 14 levels occurs.

*T*^[4,6,8]^ in Equation (41) has two bounded parameters *θ* and *φ* so several plots are required to explore this parameter space. By setting *T*^[8]^ contributions to zero the eigenvalue spectrum for *T*^[4,6]^ in Equation (40) can be plotted for changing values of *θ*, relative contributions of 4*^th^* and 6*^th^* rank tensor terms. Figure 6 plots such an eigenvalue spectrum and also places the RES plots that go along with important parts of the level diagram and, conversely, points out what spots on the level diagram correspond to important changes in the RES plot. We note *T*^[4,6]^ RES have circular ring separatrices not unlike those on *D*_2_ RES in Figure 3.

To understand the behavior of the level diagram in Figure 6 it is critical to inspect the changing shape of the RES plots. In particular, the clustering of levels in the eigenvalue diagram is dependent on the localized symmetry regions of the RES at each value of *θ*. Locally, the RES forms hills and valleys of a lower symmetry than that of the molecule. The local symmetry must also be a sub-group of the molecular symmetry. Figure 5 identifies regions of local symmetry *C*_4_, *C*_3_ and *C*_2_ whose local rotation axis lie fixed normal to the RES at the center of each region, respectively, even as *θ* varies from Figure 5(a) to Figure 5(b). For some *θ* one or two of the *C_n_* regions may shrink out of existence as shown below in Figure 6.

#### 3.3.2. Semi-Classical Outlines *vs*. Quantum Eigenvalues

**Figure 7:**
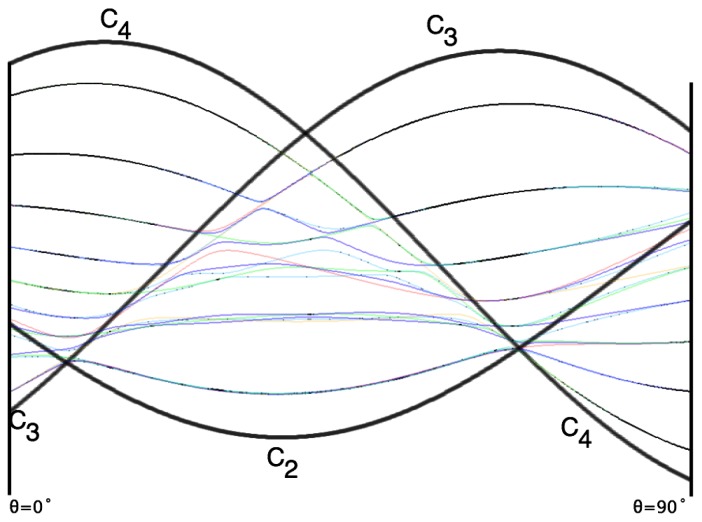
Quantum spectrum of octahedral Hamiltonian (Equation (40)) with changing *θ*. Bold lines are the energy of the classical symmetry axis labeled.

With this understanding of local subgroup regions it is possible to discuss more detail of Figure 6. The correspondence between the RES plots and the level diagram can also be seen by appending the eigenvalue spectrum in Figure 6 with the height of the *C*_4_, *C*_3_ and *C*_2_ axes. This serves two purposes: To confirm that the quantum spectrum sits inside the semi-classical boundaries and to see that there is a change in the eigenvalue spectrum corresponding to changes in RES topology. Figure 7 shows the same quantum spectrum as Figure 6, but also includes the height (energy) of each symmetry axis. The outlines are printed in bold and are labeled for which *C_n_* axis they each belong.

Section 2 described how to predict the error between a fully quantum mechanical calculation and a semi-classical approximation of the symmetric rotor rotational spectra. For the symmetric rotor this was done analytically. It is difficult to be as exact in calculating error for an octahedrally symmetric Hamiltonian, but a line plot can show when an RES plot fails to describe quantum mechanical behavior.

Rather than plotting the Hamiltonian as Equation (40) we will arrange it as

(43) $$T^{\left[4,6\right]}=(1-x)T^{\left[4\right]}+xT^{\left[6\right]}$$

This changes semi-classical outlines from cosines to lines and shows where quantum levels exceed semi-classical bounds and where an RES approximation fails. Also, *x*-line plots show by degree of avoided-crossing-curvature for each level the degree of its state mixing at *x*.

**Figure 8:**
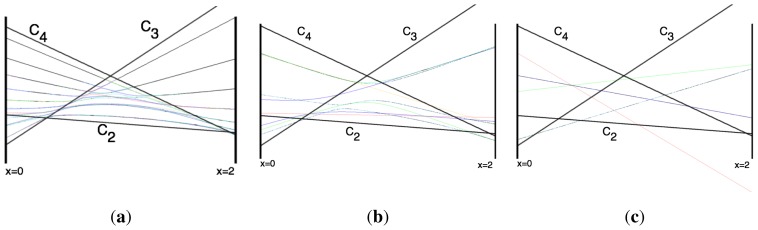
Spectrum of Octahedral Rotor Showing Semi-Classical Boundaries Given Equation (43). (**a**) J = 30; (**b**) J = 10; (**c**) J = 4.

The three plots in Figure 8 show these spectra and semi-classical outlines for *J* = 30, *J* = 10 and *J* = 4. Figure 8(a) shows that the quantum levels fit for all values of *x* at *J* = 30, while Figure 8(b) shows some small disagreement near *x* = 2 for *J* = 10. Figure 8(c) shows that for low *J* there is strong disagreement between quantum calculations and semi-classical approximations.

Indeed, such plots as Figure 6 have been created before, both for the RES plots and the level diagrams [25]. Next, we show such an analysis of *T*^[4,6,8]^ and demonstrate how such a Hamiltonian can show a different type of topology than previously reported.

#### 3.3.3. Variation of *T*^[4,6,8]^ Topography and Level Clusters

**Figure 9:**
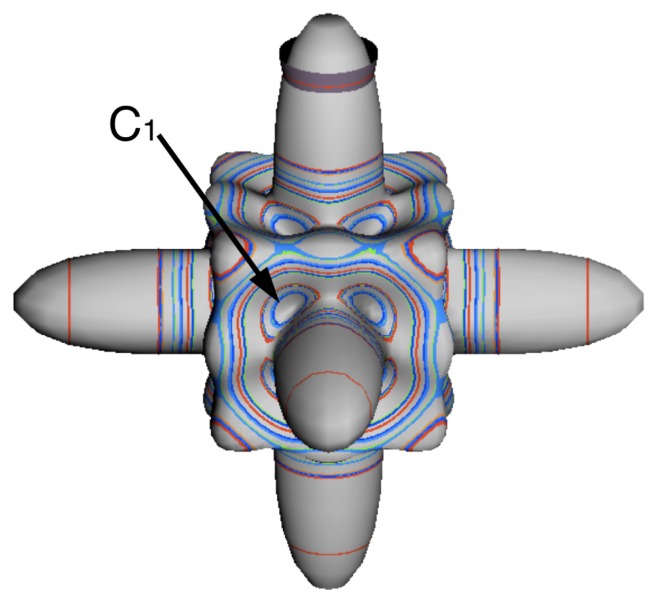
RES with *C*_1_ local symmetry regions visible.

The inclusion of eighth rank operators to the Hamiltonian dramatically changes the possible types of RES local symmetry and the related level clustering. While Figure 5 shows *C*_4_, *C*_3_ and *C*_2_ symmetric local structures for RES plots for *T*^[4,6]^ Hamiltonians, Figure 9 shows a new kind of local *T*^[4,6,8]^ RES path pointed out there with *C*_1_ symmetry. (That means *no* rotational symmetry!) The path is repeated 24 times and thus belongs to a single cluster of 24 levels. As shown in Section 6.7.2. the cluster spans an *induced* representation *D*^0_1_^ (*C*_1_) ↑ *O*, also known as a *regular* representation of *O*.

Details of the two dimensional *T*^[4,6,8]^ parameter space appear in a figure Table 10 containing RES plots for several (*θ*, *φ*) points. To be consistent with Equation (41), the plots increase *θ* from 0 to *π* going left to right and *φ* from 0 to *π* going top to bottom. RES *O* levels are colored with the usual red for *A*_1_, orange for *A*_2_, green for *E*_2_, blue for *T*_1_ and cyan for *T*_2_.

As expected from Equation (41), the top and bottom rows are opposites to one another. That is, where one RES has a hill (higher energy), the other has a crater (lower energy.) The RES at *θ* = 0, *φ* = 0 has convex *C*_4_ and concave *C*_3_ structure as does the RES at *θ* = *π*, *φ* = *π*, but opposite the shape of the RES at either *θ* = 0, *φ* = *π* or *θ* = *π*, *φ* = 0. The ordering of the levels is also opposite. These two extremal rows also have no eighth order contribution, so they produce simpler shapes than the others and are incapable of producing *C*_1_ local symmetry regions. The middle row shows a different behavior: all the diagrams are identical. Again, this follows from Equation (41) wherever *φ* = *π/*2.

**Figure 10:**
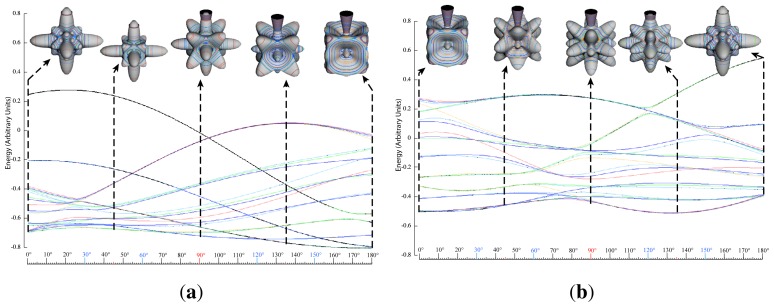
Level diagrams of energy *vs. θ* for given *φ* with RES plots at selected positions. (**a**) *φ* = *π/*4; (**b**) *φ* = 3*π/*4.

While Table 10 shows only the RES plots along the parameter space defined by Equation (41), Figure 10 shows level diagrams with RES plots placed showing the symmetry and topology present at a given point in the space. The bold vertical lines next to the RES plots indicate the spot in the level diagram that particular RES plot would exist. Again, it is clear the *θ* = *π/*2 case would be unchanged, so it is not shown. The *θ* = *π* case is neglected as it is a mirror image of the *θ* = 0 case.

### 3.4. CriteriaforC_1_ Level Clustering

**Figure 11:**
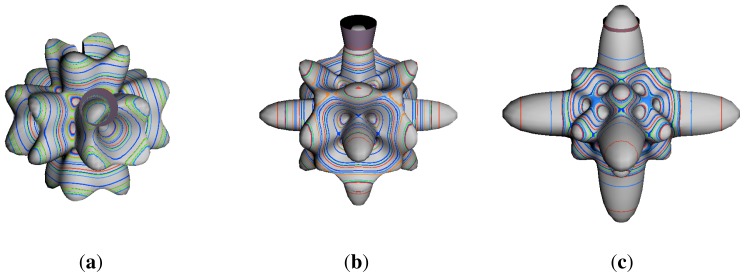
*J* = 30 RES for various rank *k*=4, 6, 8 combinations giving *C*_1_ features. (**a**) *C*_1_ hills around *C*_3_ and *C*_4_; (**b**) *C*_1_ valleys near *C*_4_; (**c**) *C*_1_ valleys near *C*_3_.

Figures 9, 10 and 11 show where the local regions of hills and valleys form on the RES depending on mixing angles *φ* and *θ*. Unlike the local symmetry regions known previously, the local *C*_1_ structures associated with 24-fold level clusters have no rotation axis to locate their central maxima or minima on the RES. However, they do have bisecting reflection planes that must contain surface gradient vectors and an extreme point for which the gradient points radially. RES plots with *C*_1_ local symmetries are shown in parts of second, third and fourth rows of Table 10 as well as parts of Figure 10(a) and 10(b). Figure 11 shows how *C*_1_ regions lie on hills or else valleys and how they can be arranged with their neighbors into either a square or triangular pattern.

*C*_1_ clusters require tensors of rank-8 with *φ* between *π* and zero as *θ* varies. Momentum *J* must be large enough for its minimum-uncertainty (*J*=*K*)-cone angle Θ*_J_^J^* to fit in a *C*_1_ region.

(44) $$\Theta_{J}^{J}={\text{cos}}^{-1}(\frac{J}{\sqrt{J+1}})≃\sqrt{\frac{1}{J}}$$

RES with *J* as low as *J*=4 may have *C*_1_ regions but fail to fit its (2Θ_4_^4^=56°)-wide cones. *C*_1_ clusters begin to appear around *J*=20 (2Θ_20_^20^=26°) but even for *J*=30 (2Θ_30_^30^=21°) are still barely formed in Figure 11(a). There a minimum uncertainty cone appears to barely fit within a separatrix on a *C*_1_ hill between *C*_3_ and *C*_4_ valleys of its (*J*=30)-RES. Others are situated more comfortably in valleys of RES shown in Figure 11(b) where they appear to encircle a *C*_4_ axes as in Figure 10(a) where a corresponding cluster of 24 eigenvalues in 10 levels appear at the lower left hand side of the level diagram. In Figure 11(c) they surround a *C*_2_ axis.

With higher *J* and *O_h_*-tensors of greater rank than *k*=8, one expects clusters of 48-*fold* degeneracy corresponding to *C*_1_-regions centered away from *O_h_* symmetry axes or planes. So far, these are only beginning to be explored and analyzed. To analyze such complicated tunneling effects (and better understand older ones) requires an improved symmetry analysis developed in Sections 4–7.

## 4. Introducing Dual Symmetry Algebra for Tunneling and Superfine Structure

For a system to have symmetry means two or more of its parts are the same or similar and therefore subject to resonance. This can make a system particularly sensitive to internal parameters and external perturbations and give rise to interesting and useful effects. However, resonances can make it more difficult to analyze and understand a system’s eigensolutions. The tensor level cluster states give rise to spectral fine structure discussed in the preceding sections and that splits further into complex *superfine* structure due to *J*-tunneling that is the focus of the following sections.

Fortunately, the presence of symmetry in a physical system allows algebraic or group theoretical analysis of quantum eigensolutions and their dynamics. Groups of operators (*g*, *g*′, *g*″, ...) leave a Hamiltonian operator *H* invariant (*g*^†^*Hg* = *H*) if and only if each *g* commutes with it (*gH* = *Hg*). Then each *g* in the group shares a set of eigenfunctions with *H*. However, if (*g*′) and (*g*) do not commute then the (*g*′) and (*g*) sets will differ.

Hamiltonians may themselves be symmetry operators or linear expansions thereof. Multipole tensor expansions used heretofore are examples. Expanding *H* into operators with symmetry properties, such as (*a*^†^*a*) or (*T_q_^k^*), helps to analyze its eigensolutions since, in some sense, a symmetry algebra “knows” its spectral resolutions. The underlying isometry of a system’s variables and states contains all the sub-algebras that are possible *H*-symmetries.

If *H*-symmetry operators (*g*, *g*′, ...) also commute with each other (*gg*′ = *g*′*g*, *etc.*) then all *g* share with *H* a single set of eigenvectors as discussed in Section 5. Such commutative or *Abelian* symmetry analysis is just a Fourier analysis where all *H* are linear expansion of its symmetry elements (*g*, *g*′, *g*″, ...) and simultaneously diagonalized with *H*. Such *g* expansions define both Hamiltonians *H* and their states as described in Section 5.

However, non-commuting (non-Abelian) symmetry operators (*g*, *g*′, *g*″, ...) of *H* cannot both expand *H* and commute with *H*. This impasse is resolved in Section 6 by using a dual *local* operator group (*ḡ*, *ḡ*′, *ḡ*″, ...) that mutually commutes with the original *global* group. Then local (*ḡ*) expand any *H* that commutes with global *g*, while the global *g* define base states and their combinations define symmetry projected states. Roughly put, one labels location while the other labels tunneling to and fro.

In Section 6, the dual group (*D̄*_3_ ~ *C̄*_3_*_v_*) of the smallest non-Abelian group (*D*_3_ ~ *C*_3_*_v_*) is defined. Dual symmetry-analysis is demonstrated for a trigonal tunneling system by group parametrization of all possible (*D*_3_)-symmetric **H** matrices and all possible eigensolutions for each. The example shows how global (*g*) label states while the local (*ḡ*) label tunneling paths. In this way symmetry labels processes as well as states. An added benefit is a kind of “slide-rule-lattice” to compute group products.

In Section 7, the local symmetry expansion is applied to octahedral *O*⊂*O_h_* tensor superfine structure. Local symmetry conditions are used to relate tunneling paths to RES topography discussed previously and predict possible energy level patterns. The *O*⊂*O_h_* slide-rule-lattices appear in Figures 22, 23 and 24.

## 5. Abelian Symmetry Analysis

An introductory analysis of tunneling symmetry begins with elementary cases involving homocyclic *C_n_* symmetry of *n*-fold polygonal structure. But, it applies to all Abelian (mutually commuting) groups *A* since all *A* reduce to outer products *C_p_* × *C_q_* × · · · of cyclic groups of prime order.

### 5.1. Operator Expansion of C_n_ Symmetric Hamiltonian

The analysis described here and in Section 6 deviates from standard procedure [27–31]. Instead of beginning with a given quantum Hamiltonian **H**-matrix, we start with a *C_n_* symmetry matrix (**r**) and build all possible *C_n_* symmetric (**H**)-matrices by combining *n* powers (**r***^p^*) = (**r**)*^p^* of (**r**) ranging from identity **r**^0^ = **1** = **r***^n^* to inverse **r***^n^*^−1^ = **r**^−1^ [32].

(45) $$\begin{array}{l} H=r_{0}r^{0}+r_{1}r^{1}+r_{2}r^{2}+\ldots +r_{n-2}r^{n-2}+r_{n-1}r^{n-1}\\ =r_{0}1+r_{1}r^{1}+r_{2}r^{2}+\ldots +r_{-2}r^{-2}+r_{-1}r^{-1} \end{array}$$

In Equation (45) the rotation **r** is by angle 2*π/n* so rotation **r***^n^* is by angle *n*2*π/n* = 2*π*, that is, the identity operator **r**^0^ = **1** = **r***^n^*. Thus power-*p* indices label *modulo*-*n* or base-*n* algebras. If *n*=2, it is a *Boolean* algebra *C*_1_ ⊂ *C*_2_ of *parity* [+1,−1] or classical *bits* [0,1]. (*U*(2) spin-algebras of *q*-*bits* have 4*π* identity but are not considered here.)

(46) $$\text{Sum rule}:p+p^{\prime }=(p+p^{\prime })\;\text{mod}\;(n)\;\text{Product rule}:p\cdot p^{\prime }=(p\cdot p^{\prime })\;\text{mod }(n)$$

We construct the general **H**-matrix using *C_n_* group-product tables shown below in a **g**^−1^**g**-form and a **g**^†^**g**-form that is equivalent for unitary operators **g**^†^ = **g**^−1^. In each table the *k^th^*-row label **g**^−1^ matches *k^th^*-column label **g** so that the identity operator **1** = **g**^−^**^1^g** resides only on the diagonal. This example is for hexagonal symmetry *C*_6_ for which **r**^−6^ = **r**^0^ = **1** = **r**^6^ = **r**^6†^, **r**^−5^ = **r**^1^ = **r**^5†^, **r**^−4^ = **r**^2^ = **r**^4†^, **r**^−3^ = **r**^3^ = **r**^3†^, and so forth.

(47) $$\begin{array}{ccccccc} \begin{array}{l} g^{-1}g\\ form \end{array} & r^{0} & r^{1} & r^{2} & r^{3} & r^{4} & r^{5}\\ r^{0} & r^{0} & r^{1} & r^{2} & r^{3} & r^{4} & r^{5}\\ r^{5} & r^{5} & r^{0} & r^{1} & r^{2} & r^{3} & r^{4}\\ r^{4} & r^{4} & r^{5} & r^{0} & r^{1} & r^{2} & r^{3}\\ r^{3} & r^{3} & r^{4} & r^{5} & r^{0} & r^{1} & r^{2}\\ r^{2} & r^{2} & r^{3} & r^{4} & r^{5} & r^{0} & r^{1}\\ r^{1} & r^{1} & r^{2} & r^{3} & r^{4} & r^{5} & r^{0} \end{array}=\begin{array}{ccccccc} \begin{array}{l} g^{†}g\\ form \end{array} & 1 & r^{+1} & r^{+2} & r^{+3} & r^{-2} & r^{-1}\\ 1 & 1 & r^{+1} & r^{+2} & r^{+3} & r^{-2} & r^{-1}\\ r^{-1} & r^{-1} & 1 & r^{+1} & r^{+2} & r^{+3} & r^{-2}\\ r^{-2} & r^{-2} & r^{-1} & 1 & r^{+1} & r^{+2} & r^{+3}\\ r^{+3} & r^{+3} & r^{-2} & r^{-1} & 1 & r^{+1} & r^{+2}\\ r^{+2} & r^{+2} & r^{+3} & r^{-2} & r^{-1} & 1 & r^{+1}\\ r^{+1} & r^{+1} & r^{+2} & r^{+3} & r^{-2} & r^{-1} & 1 \end{array}$$

The **g**^†^**g**-form produces a *regular representation R*(**g**) = (**g**) of each operator **g** as shown below. Each *R*(**r***^p^*) is a zero-matrix with a *1* inserted wherever a **r***^p^* appears in the **g**^†^**g**-table.

(48) $$\begin{array}{llll} R(1)= & R(r^{1})= & R(r^{2})= & R(r^{3})=\\ \left(\begin{array}{cccccc} 1 & \cdot & \cdot & \cdot & \cdot & \cdot \\ \cdot & 1 & \cdot & \cdot & \cdot & \cdot \\ \cdot & \cdot & 1 & \cdot & \cdot & \cdot \\ \cdot & \cdot & \cdot & 1 & \cdot & \cdot \\ \cdot & \cdot & \cdot & \cdot & 1 & \cdot \\ \cdot & \cdot & \cdot & \cdot & \cdot & 1 \end{array}\right), & \left(\begin{array}{cccccc} \cdot & 1 & \cdot & \cdot & \cdot & \cdot \\ \cdot & \cdot & 1 & \cdot & \cdot & \cdot \\ \cdot & \cdot & \cdot & 1 & \cdot & \cdot \\ \cdot & \cdot & \cdot & \cdot & 1 & \cdot \\ \cdot & \cdot & \cdot & \cdot & \cdot & 1\\ 1 & \cdot & \cdot & \cdot & \cdot & \cdot \end{array}\right), & \left(\begin{array}{cccccc} \cdot & \cdot & 1 & \cdot & \cdot & \cdot \\ \cdot & \cdot & \cdot & 1 & \cdot & \cdot \\ \cdot & \cdot & \cdot & \cdot & 1 & \cdot \\ \cdot & \cdot & \cdot & \cdot & \cdot & 1\\ 1 & \cdot & \cdot & \cdot & \cdot & \cdot \\ \cdot & 1 & \cdot & \cdot & \cdot & \cdot \end{array}\right), & \left(\begin{array}{cccccc} \cdot & \cdot & \cdot & 1 & \cdot & \cdot \\ \cdot & \cdot & \cdot & \cdot & 1 & \cdot \\ \cdot & \cdot & \cdot & \cdot & \cdot & 1\\ 1 & \cdot & \cdot & \cdot & \cdot & \cdot \\ \cdot & 1 & \cdot & \cdot & \cdot & \cdot \\ \cdot & \cdot & 1 & \cdot & \cdot & \cdot \end{array}\right) \end{array}\cdots$$

The *C_n_* Hamiltonian (**H**) matrix has matrices from (48) inserted into expansion (45) of operator **H**.

(49) $$(H)=\sum_{p=0}^{n-1}r_{p}(r^{p})=\left(\begin{array}{cccccc} r_{0} & r_{1} & r_{2} & r_{3} & r_{4} & r_{5}\\ r_{5} & r_{0} & r_{1} & r_{2} & r_{3} & r_{4}\\ r_{4} & r_{5} & r_{0} & r_{1} & r_{2} & r_{3}\\ r_{3} & r_{4} & r_{5} & r_{0} & r_{1} & r_{2}\\ r_{2} & r_{3} & r_{4} & r_{5} & r_{0} & r_{1}\\ r_{1} & r_{2} & r_{3} & r_{4} & r_{5} & r_{0} \end{array}\right)=\left(\begin{array}{cccccc} r_{0} & r_{1} & r_{2} & r_{3} & r_{-2} & r_{-1}\\ r_{-1} & r_{0} & r_{1} & r_{2} & r_{3} & r_{-2}\\ r_{-2} & r_{-1} & r_{0} & r_{1} & r_{2} & r_{3}\\ r_{3} & r_{-2} & r_{-1} & r_{0} & r_{1} & r_{2}\\ r_{2} & r_{3} & r_{-2} & r_{-1} & r_{0} & r_{1}\\ r_{1} & r_{2} & r_{3} & r_{-2} & r_{-1} & r_{0} \end{array}\right)$$

Matrices in Equation (49) are simply group tables Equation (47) with complex tunneling amplitude *r_p_* replacing operator **r***^p^*. Parameters *r*_0_ = (*r*_0_)^*^ and *r*_3_ = (*r*_3_)^*^ match self-conjugate binary subgroups *C*_1_ ⊂ *C*_2_ = (**1**, **r**^3^) related by **1** = (**r**^3^)^2^. Both are real if matrix (**H**) is Hermitian self-conjugate (*H_ab_* = H_ba_^*^).

**Figure 12:**
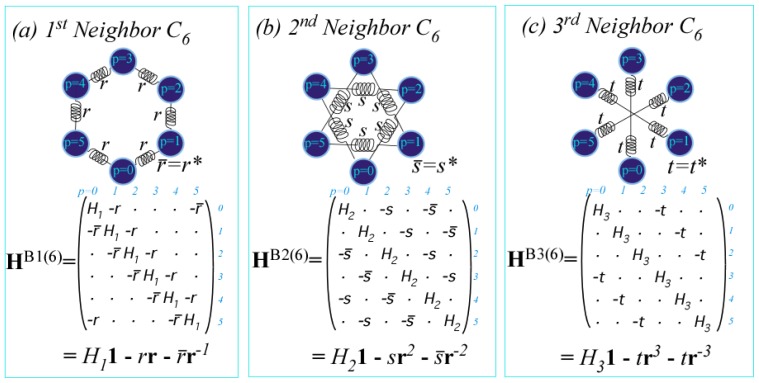
Three classes of tunneling paths and parameters.

Three distinct classes of tunneling or coupling parameters are depicted in Figure 12 using classical spring-mass analogs for quantum systems [22]. Tunneling matrices have a long history [33] going back to Wilson [34]. Here this is being revived to treat extreme *J*-tunneling and more recently by Ortigoso [17] and Hougen [35,36] to treat extremely floppy molecule dynamics. Both these tasks use tunneling parametrization that has so far been quite *ad.hoc.* To accomplish either of these tasks, or what will surely be needed, namely *both* tasks, we need a tighter symmetry analysis. The group operator scheme being introduced here seeks a way to achieve this.

The 1*^st^*-neighbor class has non-zero parameters *r*_1_=−*r* and conjugate *r*_−1_=−r^*^=−*r̄* coupling only nearest neighbors each with self-energy *r*_0_=*H*_1_. The 2*^nd^*-neighbor class has non-zero parameters *r*_2_=−*s* and conjugate *r*_−2_=−s^*^=−*s̄* coupling only next-nearest neighbors with self-energy *r*_0_=*H*_2_. Finally, 3*^rd^*-neighbor coupling *r*_3_=−*t*=−t^*^ is real as required for binary self-conjugacy **r**^3^=(**r**^3^)^†^.

### 5.2. Spectral Resolution of C_n_ Symmetry Operators

Eigenvalues *χ_p_^m^* of each operator **r***^p^* are *m^th^* multiples of *n^th^*-roots of unity since all *C_n_* symmetry operators **g** = **r***^p^* satisfy **g***^n^* = **1** and are *characters* of *C_n_* symmetry operators. Magnetic or *mode*-wavenumber indices *m* label a base-*n* algebra as do the power or position-*point* indices *p* in Equation (46). Spatial lattice points *x_p_* = *L*·*p*(*meters*) are indexed by *p* while reciprocal-(*k*)-wavevector space *k_m_* = 2*πm/L*(*per meter*) is indexed by integer *m*.

(50) $${\langle r^{p}\rangle }_{m}=\chi_{p}^{m}=e^{-i(m\cdot p)2\pi /n}=e^{-ik_{m}x_{p}}=D^{\left(m\right)}(r^{p})$$

The *χ_p_^m^* are *C_n_* irreducible representations *D*^(^*^m^*^)^(**r***^p^*) as well as *C_n_* characters. General group characters are traces (diagonal sums) of D-matrices (*χ*^(^*^m^*^)^(**g**) = *traceD*^(^*^m^*^)^(**g**)). Abelian group irreducible representations are 1-dimensional due to their commutativity, and so for them characters and representations are identical. (*χ*^(^*^m^*^)^(**g**) = *D*^(^*^m^*^)^(**g**)) All this is generalized in subsequent Section 6. Any number of mutually commuting unitary matrices may be diagonalized by a single unitary transformation matrix. The characters in Equation (50) form a unitary transformation matrix *T_m_*,*_p_* that diagonalizes each *C_n_* matrix (**r***^p^*).

(51) $$T_{m,p}=\chi_{p}^{m}/\sqrt{n}$$

**Figure 13:**
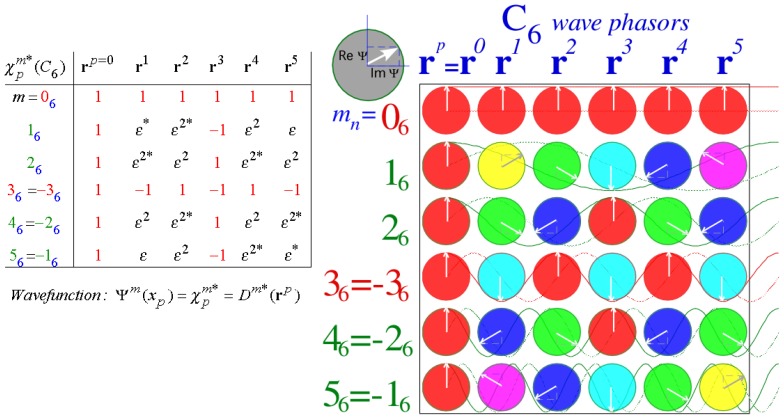
*C*_6_ characters (**a**) numerical table; (**b**) wave phasor table.

This *T* is a discrete (*n*-*by*-*n*) Fourier transformation. A 6-*by*-6 example that diagonalizes all matrices in Equations (48) and (49) and in Figure 12 is shown in Figure 13 by a character table of wave phasors based on *D*^(^*^m^*^)^(**r***^p^*) in Equation (50) or (51). The irreducible representations *D*^(^*^m^*^)^(**r***^p^*) or *irreps* play multiple roles. They are variously eigenvalues, eigenvectors, eigenfunctions, transformation components, and Fourier components of dispersion relations. This hyper-utility centers on their role as coefficients in *spectral resolution* of operators **r***^p^* into idempotent projection operators **P**^(^*^m^*^)^. **P**^(^*^m^*^)^ are like irrep *placeholders*.

(52) $$r^{p}=\sum_{m=0}^{n-1}\chi_{p}^{m}P^{\left(m\right)}=\chi_{p}^{0}P^{\left(0\right)}+\chi_{p}^{1}P^{\left(1\right)}+\chi_{p}^{2}P^{\left(2\right)}+\chi_{p}^{3}P^{\left(3\right)}+\chi_{p}^{4}P^{\left(4\right)}+\chi_{p}^{5}P^{\left(5\right)}$$

Equation (52) is column-*p* of Figure 13. Column-0 is a *completeness* or *identity resolution* relation.

(53) $$r^{0}=\sum_{m=0}^{n-1}P^{\left(m\right)}=1=P^{\left(0\right)}+P^{\left(1\right)}+P^{\left(2\right)}+P^{\left(3\right)}+P^{\left(4\right)}+P^{\left(5\right)}$$

Dirac notation for **P**^(^*^m^*^)^ is |(*m*)〉〈(*m*)|. Its representation in its own basis (*eigenbasis*) is simply a zero matrix with a single 1 at the (*m*,*m*)-diagonal component. **P**^(^*^m^*^)^-product table in Equation (54) is equivalent through Equation (52) to **g**-product table in Equation (47) but the **P**^(^*^m^*^)^-table given below has an orthogonal (*e.g.***P**^(1)^**P**^(2)^ = **0**) idempotent (*e.g.***P**^(1)^**P**^(1)^ = **P**^(1)^) form.

(54) $$\begin{array}{l} P^{\left(m\right)}P^{\left(n\right)}=\delta^{mn}P^{\left(n\right)}\to \begin{array}{ccccccc} \begin{array}{l} P^{†}P\\ form \end{array} & P^{\left(0\right)} & P^{\left(1\right)} & P^{\left(2\right)} & P^{\left(3\right)} & P^{\left(4\right)} & P^{\left(5\right)}\\ P^{\left(0\right)} & P^{\left(0\right)} & \cdot & \cdot & \cdot & \cdot & \cdot \\ P^{\left(1\right)} & \cdot & P^{\left(1\right)} & \cdot & \cdot & \cdot & \cdot \\ P^{\left(2\right)} & \cdot & \cdot & P^{\left(2\right)} & \cdot & \cdot & \cdot \\ P^{\left(3\right)} & \cdot & \cdot & \cdot & P^{\left(3\right)} & \cdot & \cdot \\ P^{\left(4\right)} & \cdot & \cdot & \cdot & \cdot & P^{\left(4\right)} & \cdot \\ P^{\left(5\right)} & \cdot & \cdot & \cdot & \cdot & \cdot & P^{\left(5\right)} \end{array}\\ \to {\left(P^{\left(2\right)}\right)}_{P}=\left(\begin{array}{cccccc} \cdot & \cdot & \cdot & \cdot & \cdot & \cdot \\ \cdot & \cdot & \cdot & \cdot & \cdot & \cdot \\ \cdot & \cdot & 1 & \cdot & \cdot & \cdot \\ \cdot & \cdot & \cdot & \cdot & \cdot & \cdot \\ \cdot & \cdot & \cdot & \cdot & \cdot & \cdot \\ \cdot & \cdot & \cdot & \cdot & \cdot & \cdot \end{array}\right) \end{array}$$

The location of each **P**^(^*^m^*^)^ in the **P**-table is a location of a 1 in its representation as indicated in the right hand side of Equation (54) in the same way that locations in **g**-table Equation (47) place 1’s in representations Equation (48). However, idempotent self-conjugacy (**P**^†^ = **P**) makes row labels of **P**-table Equation (54) identical to its column labels, whereas only **g** = **1** and **g** = **r^3^** are self-conjugate in **g**-table Equation (47).

Character arrays such as Figure 13 represent operator eigen-products between **r***^p^* and **P**^(^*^m^*^)^.

(55) $$r^{p}P^{\left(m\right)}=\chi_{p}^{m}P^{\left(m\right)}=P^{\left(m\right)}r^{p}$$

Also character *χ_p_^m^* is the scalar product *overlap* of position state bra or ket with momentum ket or bra.

(56a) $$\begin{array}{l} \text{Position bra}:\;\;\;\langle x_{p}|=\langle p|=\langle 0|r^{-p}\\ \text{Position ket}:\;\;\;|x_{p}\rangle =|p\rangle =r^{p}|0\rangle \end{array}$$

(56b) $$\begin{array}{l} \text{Momentum bra}:\langle k_{m}|=\langle (m)|=\langle 0|P^{\left(m\right)}\sqrt{n}\\ \text{Momentum ket}:|k_{m}\rangle =|(m)\rangle =P^{\left(m\right)}|0\rangle \sqrt{n} \end{array}$$

Momentum eigenwave *ψ_k_*_*_m_*_ (*x_p_*) is character Equation (50) conjugated to *e^ik^*^_*m*_^*^x^*^_*p*_^ and normalized by $\sqrt{n}$.

(57) $$\begin{array}{l} \psi_{k_{m}}(x_{p})=\langle x_{p}|k_{m}\;\rangle =\langle p|(m)\rangle ={\left(\chi_{p}^{m}/\sqrt{n}\right)}^{*}\\ ={\left(\langle (m)|p\rangle \right)}^{*}=e^{ik_{m}x_{p}}/\sqrt{n} \end{array}$$

Action of **r***^p^* on *m*-ket |(*m*)〉 = |*k_m_*〉 is conjugate and inverse to action on coordinate bra 〈*x_q_*| = 〈*q*|.

(58) $$\begin{array}{l} \psi_{k_{m}}(x_{q}-p\cdot L)=\langle x_{q}|r^{p}|k_{m}\rangle =\langle q|r^{p}|(m)\rangle \\ =\langle q-p|(m)\rangle =e^{-ik_{m}x_{p}}\langle q|(m)\rangle \end{array}$$

The same overlap results whether **r***^p^* moves a (*m*)-wave *p*-points forward or moves the coordinate grid *p*-points backward. This *C_n_* relativity-duality principle generalizes to non-Abelian symmetry and is key to operator labeling of coordinates, base states, Hamiltonians, and their eigensolutions.

**P**^(^*^m^*^)^ projects *m*-states with conjugate characters *φ_p_^m^*= (*χ_p_^m^*)^*^ with factor 1*/n* so **P**^(^*^m^*^)^’s are idempotent and sum to **1**. (∑*_p_***P**^(^*^m^*^)^ = **1**) But, |*k_m_*〉 has *φ_p_^m^* with factor $1/\sqrt{n}$ to be orthonormal so its *squares* sum to 1. (∑*_p_*|〈*x_p_*|*k_m_*〉|^2^ = 1) Thus projection Equation (56b) of |*k_m_*〉 by **P**^(^*^m^*^)^ has a factor $\sqrt{n}$. Inverse spectral resolution Equation (52) sums over column points *p* using *φ_p_^m^* from each row-(*m*) of Figure 13. Factor 1*/n* makes **P**^(^*^m^*^)^ complete (∑*_m_***P**^(^*^m^*^)^ = **1** in Equation (53)) and idempotent (**P**^(^*^m^*^)^**P**^(^*^m^*^)^ = **P**^(^*^m^*^)^) in Equation (54)).

(59) $$P^{\left(m\right)}=\left(\sum_{p=0}^{n-1}\varphi_{p}^{m}r^{p}\right)/n=(\varphi_{0}^{m}r^{0}+\varphi_{1}^{m}r^{1}+\varphi_{2}^{m}r^{2}+\varphi_{3}^{m}r^{3}+\varphi_{4}^{m}r^{4}+\varphi_{5}^{m}r^{5})/6$$

First row ((*m*)=(0)-row) of Figure 13 is an average, *i.e*., sum of all symmetry operators weighted by 1*/n*.

(60) $$P^{\left(0\right)}=\left(\sum_{p=0}^{n-1}r^{p}\right)/n=(r^{0}+r^{1}+r^{2}+r^{3}+r^{4}+r^{5})/6$$

Thus factors $\sqrt{n}=\sqrt{6}$ in state projections in Equation (56b) give state norms $\sqrt{n}/n=1/\sqrt{n}$ in Equation (57).

(61) $$P^{\left(m\right)}|0\rangle \sqrt{n}=\left(\sum_{p=0}^{n-1}\varphi_{p}^{m}|p\rangle \right)/\sqrt{n}=(\varphi_{0}^{m}|0\rangle +\varphi_{1}^{m}|1\rangle +\varphi_{2}^{m}|2\rangle +\varphi_{3}^{m}|3\rangle +\varphi_{4}^{m}|4\rangle +\varphi_{5}^{m}|5\rangle )/\sqrt{6}$$

The (0)-momentum or *scalar* state is a sum over the (*m*)=(0)-row of Figure 13 normalized by $1/\sqrt{n}$.

(62) $$P^{\left(0\right)}|0\rangle \sqrt{n}=\left(\sum_{p=0}^{n-1}|p\rangle \right)/\sqrt{n}=(|0\rangle +|1\rangle +|2\rangle +|3\rangle +|4\rangle +|5\rangle )/\sqrt{6}$$

### 5.3. Spectral Resolution of C_n_ Symmetric Hamiltonian

**Figure 14:**
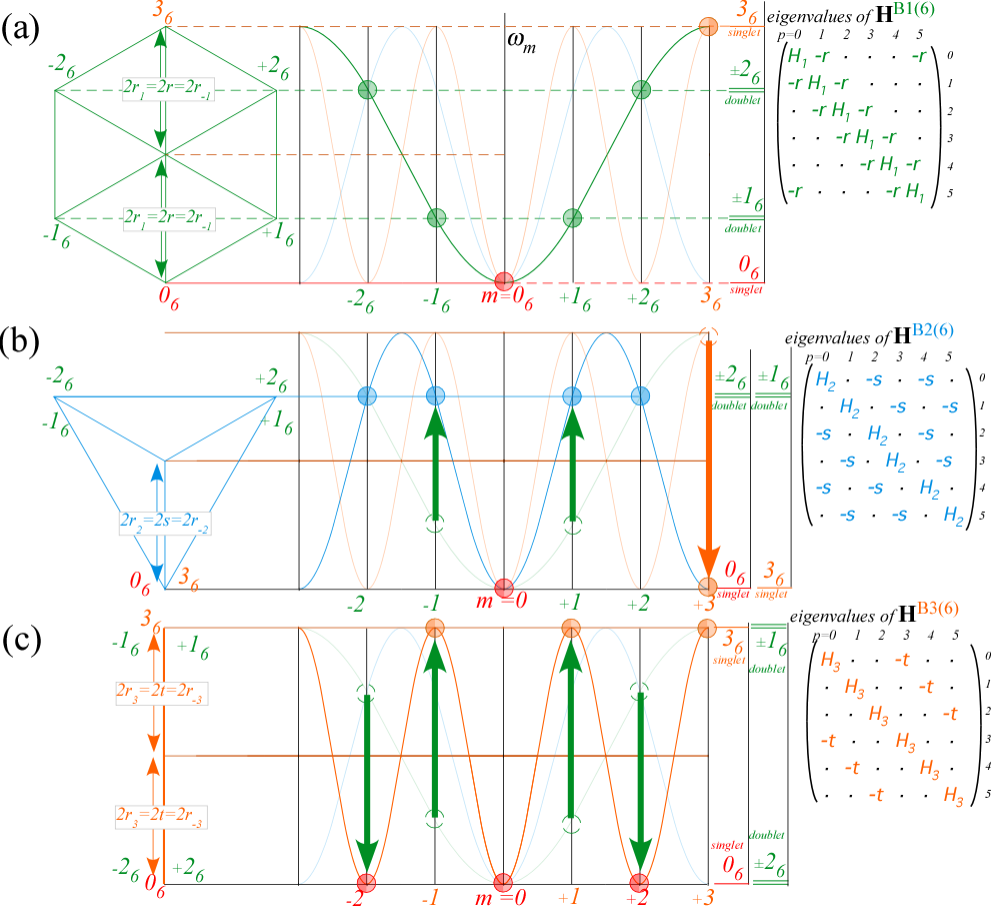
Energy level dispersion for architypical tunneling parameters: B1:*r*_1_ = −*r*, B2:*r*_2_ = −*s*, B3:*r*_3_ = −*t*.

Given Hamiltonian **H** expansion in Equation (45) in operators **r***^p^* and the spectral resolution in Equation (52) of **r***^p^*, there follows the desired spectral resolution of **H**. The eigenvalue coefficients *ω*^(^*^m^*^)^ of **P**^(^*^m^*^)^ define the dispersion function *ω*(*k_m_*) of **H** in Figure 14(a) where it is conventional to center scalar origin (*m*)=(0).

(63) $$H=\sum_{p=0}^{n-1}r_{p}r^{p}=\sum_{p=0}^{n-1}r_{p}\sum_{m=0}^{n-1}\chi_{p}^{m}P^{\left(m\right)}=\sum_{m=0}^{n-1}\omega^{\left(m\right)}P^{\left(m\right)}\;\;\;where:\omega^{\left(m\right)}=\sum_{p=0}^{n-1}r_{p}\chi_{p}^{m}=\omega (k_{m})$$

Positive *k_m_*-axis *C*_6_ array [...(0), (1), (2), (3), (4), (5), ...] of Equation (54) shifts to a zone-center-array *mod*-6: [...(4), (5), (0), (1), (2), (3), ...]=[...(−2), (−1), (0), (1), (2), (3), ...] using Equation (46).

Examples of dispersion relations for three classes of tunneling paths in Figure 12 are shown in Figure 14. Dispersion *ω*(*k_m_*) for *C*_6_ symmetry depends sensitively on the Hamiltonian tunneling amplitudes *r_p_* for −3 *< p* ≤ 3 (or 0 ≤ *p <* 6) in Equation (49), and for any set of eigenvalues *ω*(*k_m_*) there is a unique set of *r_p_* found by inverting Equation (63).

(64) $$r_{p}=\sum_{m=0}^{n-1}\varphi_{p}^{m}\omega^{\left(m\right)}/n\;\;\;where:\varphi_{p}^{m}={\left(\chi_{p}^{m}\right)}^{*}$$

A common tunneling spectral model is the elementary Bloch 1*^st^*-neighbor *B*1(6)-model shown in Figure 14a, much like that developed in Reference [33]. For negative values of *r*_1_=−*r*, a *B*1(6) spectra for *C*_6_ consist of six points on a single inverted cosine-wave curve centered at *m*=0 with its maxima at the *Brillouin*-*band boundaries* (*m*)=±3. This curve applies to *B*1(*n*) spectra for *C_n_* where *n* equally spaced (*m*) points lie on the dispersion curve between *m*=±*n/*2 for even-*n*. The *n* energy eigenvalues *ω*^(^*^m^*^)^ are projections of an *n*-polygon. For *n*=6 that is the hexagon shown in Figure 14a projecting two doublet levels *ω*^(±1)^ and *ω*^(±2)^ between singlet *ω*^(0)^ and singlet *ω*^(3)^ at lowest and highest hexagonal vertices as follows from Equation (63).

(65) $$\omega^{B1(n)}(k_{m})=r_{0}\chi_{0}^{m}+r_{1}\chi_{1}^{m}+r_{-1}\chi_{-1}^{m}=H_{1}-2r\;cos(2\pi m/6)$$

The 2*^nd^*-neighbor *B*2-model (Figure 14b) has a two-cosine-wave dispersion curve. An equilateral triangle projects energy doublet levels [*ω*^(0)^, *ω*^(3)^] from its lowest vertex and a quartet [*ω*^(±1)^, *ω*^(±2)^] from its upper vertices.

(66) $$\omega^{B2(n)}(k_{m})=r_{0}\chi_{0}^{m}+r_{2}\chi_{2}^{m}+r_{-2}\chi_{-2}^{m}=H_{2}-2s\;cos(4\pi m/6)$$

The 3*^rd^*-neighbor *B*3-model (Figure 14c) has a three-cosine-wave dispersion, which for *n*=6 and *r*_3_=−*t* separates levels into an even-*m* triplet [*ω*^(0)^, *ω*^(±2)^] below an odd-*m* triplet [*ω*^(3)^, *ω*^(±1)^].

(67) $$\omega^{B3(n)}(k_{m})=r_{0}\chi_{0}^{m}+r_{3}\chi_{3}^{m}+r_{-3}\chi_{-3}^{m}=H_{3}-2t\;{\left(-1\right)}^{m})$$

**Figure 15:**
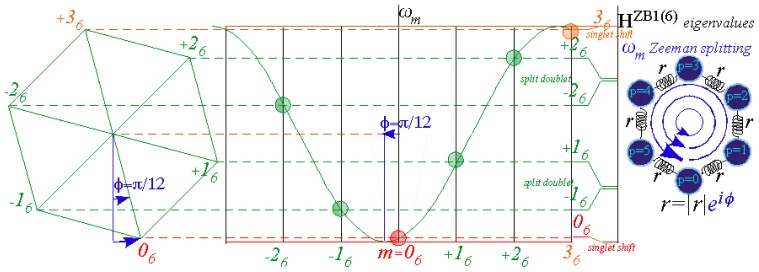
Zeeman shifted Bloch dispersion for complex parameter in ZB1(6) model: *r*_1_ = −*re^iφ^* with *φ* = *π/*12.

Combining of *k^th^*-neighbor *r_k_*-terms gives dispersion *ω*^(^*^m^*^)^ as a *k*-term Fourier cosine series that is, for real *r_k_*, a sum of the preceding three Equations (65)–(67). However, real *r_k_* imply symmetry that is higher than *C*_6_, namely non-Abelian reflection-rotation symmetry such as *C*_6_*_v_* or *D*_6_*_h_* and a corresponding degeneracy between *ω*^(±^*^m^*^)^ levels that will be treated shortly. Simple *C*_6_ symmetry allows six real parameters with complex *r*_1_ and *r*_2_. Then Equation (63) implies six levels that are generally non-degenerate as shown in Figure 15. Complex *r*_1_ = |*r*|*e^iφ^* of a ZB1 model describes chiral magnetic or rotational effects that include Zeeman-like splitting of *m*-doublets. The projecting hexagon tilts by the “gauge” phase angle *φ* = *π/*12 as the ZB1(6) dispersion *ω*(*m*) shifts. Then *m* doublets (±1) and (±2) suffer splittings that are 1*^st^*-order in *φ* while singlets (0) and (3) undergo shifts that are 2*^nd^*-order in *φ*.

## 6. Non-Abelian Symmetry Analysis

**Figure 16:**
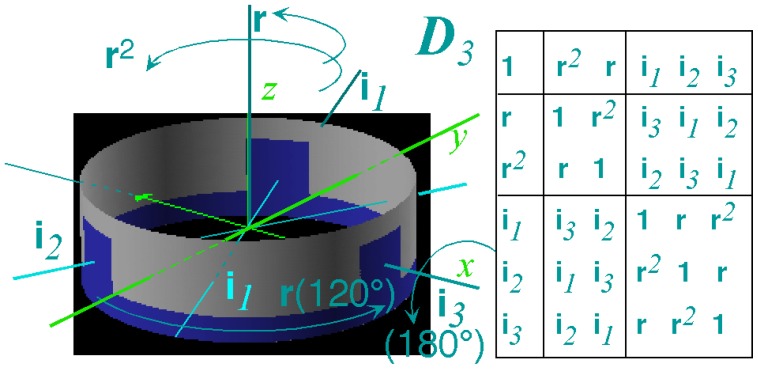
Rotation operators [**1**, **r^1^**, **r^2^**, **i_1_**, **i_2_**, **i_3_**] for a *D*_3_ symmetric square-well potential.

Characterization and spectral resolution in Equation (63) of a Hamiltonian **H***^Bk^*^(6)^ uses its expansion in Equation (45) in Abelian group *C*_6_. Similar spectral resolution of a Hamiltonian **H** by a non-Abelian group *G* = [...**g**_1_, **g**_2_...] of non-commuting symmetry operators might seem impossible. To be symmetry operators of **H**, elements **g**_1_ and **g**_2_ must commute with **H**, but that cannot be if **H** is a linear expansion of them like Equation (45). The impasse is broken by introducing operator *relativity*-*duality* detailed below. A *D*_3_-symmetric tunneling **H** with a 3-well potential sketched in Figure 16 is used as an example.

### 6.1. Operator Expansion of D_3_ Symmetric Hamiltonian

The simplest non-Abelian group is the rotational symmetry *D*_3_ = [**1**, **r**^1^, **r**^2^, **i**_1_, **i**_2_, **i**_3_] of an equilateral triangle. *D*_3_ is used to show how to generalize *C*_6_ operator analysis of the preceding section to any symmetry group. The *D*_3_ analysis begins with a **g**^†^**g**-form of group product table like Equation (47) for *C*_6_. However, *D*_3_ also requires a **gg**^†^-form giving the same product rules but using inverse **g**^†^ ordering |..**r**^2^, **r**^1^, ...|=|..**r**^1†^, **r**^2†^, ...| along the top instead of down the left side as is done for the **g**^†^**g**-form of table. (The two ±120° rotations **r**^1^ and **r**^2^ are the only pair (**r**^1†^=**r**^2^) to be switched by conjugation). The three ±180° rotations are each self-conjugate (**i***_p_*^†^=**i***_p_*) as is (always) the identity **1**^†^=**1**.

(68) $$\begin{array}{ccccccc} \begin{array}{l} g^{†}g\\ form \end{array} & 1 & r^{1} & r^{2} & i_{1} & i_{2} & i_{3}\\ 1 & 1 & r^{1} & r^{2} & i_{1} & i_{2} & i_{3}\\ r^{2} & r^{2} & 1 & r^{1} & i_{2} & i_{3} & i_{1}\\ r^{1} & r^{1} & r^{2} & 1 & i_{3} & i_{1} & i_{2}\\ i_{1} & i_{1} & i_{2} & i_{3} & 1 & r^{1} & r^{2}\\ i_{2} & i_{2} & i_{3} & i_{1} & r^{2} & 1 & r^{1}\\ i_{3} & i_{3} & i_{1} & i_{2} & r^{1} & r^{2} & 1 \end{array}\;\;\;\begin{array}{ccccccc} \begin{array}{l} gg^{†}\\ form \end{array} & \bar{1} & {\bar{r}}^{2} & {\bar{r}}^{1} & {\bar{i}}_{1} & {\bar{i}}_{2} & {\bar{i}}_{3}\\ \bar{1} & \bar{1} & {\bar{r}}^{2} & {\bar{r}}^{1} & {\bar{i}}_{1} & {\bar{i}}_{2} & {\bar{i}}_{3}\\ {\bar{r}}^{1} & {\bar{r}}^{1} & \bar{1} & {\bar{r}}^{2} & {\bar{i}}_{3} & {\bar{i}}_{1} & {\bar{i}}_{2}\\ {\bar{r}}^{2} & {\bar{r}}^{2} & {\bar{r}}^{1} & \bar{1} & {\bar{i}}_{2} & {\bar{i}}_{3} & {\bar{i}}_{1}\\ {\bar{i}}_{1} & {\bar{i}}_{1} & {\bar{i}}_{3} & {\bar{i}}_{2} & \bar{1} & {\bar{r}}^{1} & {\bar{r}}^{2}\\ {\bar{i}}_{2} & {\bar{i}}_{2} & {\bar{i}}_{1} & {\bar{i}}_{3} & {\bar{r}}^{2} & \bar{1} & {\bar{r}}^{1}\\ {\bar{i}}_{3} & {\bar{i}}_{3} & {\bar{i}}_{2} & {\bar{i}}_{1} & {\bar{r}}^{1} & {\bar{r}}^{2} & \bar{1} \end{array}$$

Over-bar notation is used for *dual*-*group D̄*_3_ = [**1̄, r̄**^1^, **r̄**^2^, **ī**_1_, **ī**_2_, **ī**_2_] of “body”-based operators isomorphic to “lab”-based group.

Matrix representations Equation (69a) for *D*_3_ or matrices Equation (69b) for *D̄*_3_ are given, respectively, by **g**^†^**g** or **gg**^†^-forms Equation (68) just as **g**^†^**g** form Equation (47) for *C*_6_ gives matrices in Equation (48).

(69a) $$\begin{array}{lll} (1)= & (r^{1})= & (r^{2})=\\ \left(\begin{array}{cccccc} 1 & \cdot & \cdot & \cdot & \cdot & \cdot \\ \cdot & 1 & \cdot & \cdot & \cdot & \cdot \\ \cdot & \cdot & 1 & \cdot & \cdot & \cdot \\ \cdot & \cdot & \cdot & 1 & \cdot & \cdot \\ \cdot & \cdot & \cdot & \cdot & 1 & \cdot \\ \cdot & \cdot & \cdot & \cdot & \cdot & 1 \end{array}\right) & \left(\begin{array}{cccccc} \cdot & 1 & \cdot & \cdot & \cdot & \cdot \\ \cdot & \cdot & 1 & \cdot & \cdot & \cdot \\ 1 & \cdot & \cdot & \cdot & \cdot & \cdot \\ \cdot & \cdot & \cdot & \cdot & 1 & \cdot \\ \cdot & \cdot & \cdot & \cdot & \cdot & 1\\ \cdot & \cdot & \cdot & 1 & \cdot & \cdot \end{array}\right) & \left(\begin{array}{cccccc} \cdot & \cdot & 1 & \cdot & \cdot & \cdot \\ 1 & \cdot & \cdot & \cdot & \cdot & \cdot \\ \cdot & 1 & \cdot & \cdot & \cdot & \cdot \\ \cdot & \cdot & \cdot & \cdot & \cdot & 1\\ \cdot & \cdot & \cdot & 1 & \cdot & \cdot \\ \cdot & \cdot & \cdot & \cdot & 1 & \cdot \end{array}\right)\\ (i_{1})= & (i_{2})= & (i_{3})=\\ \left(\begin{array}{cccccc} \cdot & \cdot & \cdot & 1 & \cdot & \cdot \\ \cdot & \cdot & \cdot & \cdot & \cdot & 1\\ \cdot & \cdot & \cdot & \cdot & 1 & \cdot \\ 1 & \cdot & \cdot & \cdot & \cdot & \cdot \\ \cdot & \cdot & 1 & \cdot & \cdot & \cdot \\ \cdot & 1 & \cdot & \cdot & \cdot & \cdot \end{array}\right) & \left(\begin{array}{cccccc} \cdot & \cdot & \cdot & \cdot & 1 & \cdot \\ \cdot & \cdot & \cdot & 1 & \cdot & \cdot \\ \cdot & \cdot & \cdot & \cdot & \cdot & 1\\ \cdot & 1 & \cdot & \cdot & \cdot & \cdot \\ 1 & \cdot & \cdot & \cdot & \cdot & \cdot \\ \cdot & \cdot & 1 & \cdot & \cdot & \cdot \end{array}\right) & \left(\begin{array}{cccccc} \cdot & \cdot & \cdot & \cdot & \cdot & 1\\ \cdot & \cdot & \cdot & \cdot & 1 & \cdot \\ \cdot & \cdot & \cdot & 1 & \cdot & \cdot \\ \cdot & \cdot & 1 & \cdot & \cdot & \cdot \\ \cdot & 1 & \cdot & \cdot & \cdot & \cdot \\ 1 & \cdot & \cdot & \cdot & \cdot & \cdot \end{array}\right) \end{array}$$

(69b) $$\begin{array}{lll} (\bar{1})= & ({\bar{r}}^{1})= & ({\bar{r}}^{2})=\\ \left(\begin{array}{cccccc} 1 & \cdot & \cdot & \cdot & \cdot & \cdot \\ \cdot & 1 & \cdot & \cdot & \cdot & \cdot \\ \cdot & \cdot & 1 & \cdot & \cdot & \cdot \\ \cdot & \cdot & \cdot & 1 & \cdot & \cdot \\ \cdot & \cdot & \cdot & \cdot & 1 & \cdot \\ \cdot & \cdot & \cdot & \cdot & \cdot & 1 \end{array}\right) & \left(\begin{array}{cccccc} \cdot & \cdot & 1 & \cdot & \cdot & \cdot \\ 1 & \cdot & \cdot & \cdot & \cdot & \cdot \\ \cdot & 1 & \cdot & \cdot & \cdot & \cdot \\ \cdot & \cdot & \cdot & \cdot & 1 & \cdot \\ \cdot & \cdot & \cdot & \cdot & \cdot & 1\\ \cdot & \cdot & \cdot & 1 & \cdot & \cdot \end{array}\right) & \left(\begin{array}{cccccc} \cdot & 1 & \cdot & \cdot & \cdot & \cdot \\ \cdot & \cdot & 1 & \cdot & \cdot & \cdot \\ 1 & \cdot & \cdot & \cdot & \cdot & \cdot \\ \cdot & \cdot & \cdot & \cdot & \cdot & 1\\ \cdot & \cdot & \cdot & 1 & \cdot & \cdot \\ \cdot & \cdot & \cdot & \cdot & 1 & \cdot \end{array}\right)\\ ({\bar{i}}_{1})= & ({\bar{i}}_{2})= & ({\bar{i}}_{3})=\\ \left(\begin{array}{cccccc} \cdot & \cdot & \cdot & 1 & \cdot & \cdot \\ \cdot & \cdot & \cdot & \cdot & 1 & \cdot \\ \cdot & \cdot & \cdot & \cdot & \cdot & 1\\ 1 & \cdot & \cdot & \cdot & \cdot & \cdot \\ \cdot & 1 & \cdot & \cdot & \cdot & \cdot \\ \cdot & \cdot & 1 & \cdot & \cdot & \cdot \end{array}\right) & \left(\begin{array}{cccccc} \cdot & \cdot & \cdot & \cdot & 1 & \cdot \\ \cdot & \cdot & \cdot & \cdot & \cdot & 1\\ \cdot & \cdot & \cdot & 1 & \cdot & \cdot \\ \cdot & \cdot & 1 & \cdot & \cdot & \cdot \\ 1 & \cdot & \cdot & \cdot & \cdot & \cdot \\ \cdot & 1 & \cdot & \cdot & \cdot & \cdot \end{array}\right) & \left(\begin{array}{cccccc} \cdot & \cdot & \cdot & \cdot & \cdot & 1\\ \cdot & \cdot & \cdot & 1 & \cdot & \cdot \\ \cdot & \cdot & \cdot & \cdot & 1 & \cdot \\ \cdot & 1 & \cdot & \cdot & \cdot & \cdot \\ \cdot & \cdot & 1 & \cdot & \cdot & \cdot \\ 1 & \cdot & \cdot & \cdot & \cdot & \cdot \end{array}\right) \end{array}$$

Most pairs of resulting *D*_3_ matrices in Equation (69a) do not commute. (For example (**r^1^**)(**i_1_**)=(**i_3_**) does not equal (**i_1_**)(**r^1^**)=(**i_2_**).) Identical non-commutative product rules apply to the dual bar group *D̄*_3_ matrices in Equation (69b). However, all matrices of the latter *D̄*_3_ commute with all matrices of the former *D*_3_. This suggests that the Hamiltonian matrix, in order to commute with its symmetry group *D*_3_, is constructed by linear combination of bar group operators of *D̄*_3_ [32].

(70) $$H=r_{0}\bar{1}+r_{1}{\bar{r}}^{1}+r_{2}{\bar{r}}^{2}+i_{1}{\bar{i}}_{1}+i_{2}{\bar{i}}_{2}+i_{3}{\bar{i}}_{3}$$

*D*_3_ symmetric (**H**) matrix Equation (71) generalizes *C*_6_ symmetric (**H**) matrix Equation (49) to a non-Abelian case.

(71) $$(H)=\sum_{g=1}^{{}^{\circ}G}r_{g}(\bar{g})=\left(\begin{array}{cccccc} r_{0} & r_{2} & r_{1} & i_{1} & i_{2} & i_{3}\\ r_{1} & r_{0} & r_{2} & i_{3} & i_{1} & i_{2}\\ r_{2} & r_{1} & r_{0} & i_{2} & i_{3} & i_{1}\\ i_{i} & i_{3} & i_{2} & r_{0} & r_{1} & r_{2}\\ i_{2} & i_{1} & i_{3} & r_{2} & r_{0} & r_{1}\\ i_{3} & i_{2} & i_{1} & r_{1} & r_{2} & r_{0} \end{array}\right)$$

### 6.2. Spectral Resolution of D_3_ Symmetry Operators

Spectral resolution of *D*_3_ or any non-Abelian group *G* = [...**g**_1_, **g**_2_...] entails more than the *C*_6_ expansion into a unique combination of idempotent operators **P***^α^*=|*α*〉〈*α*| multiplied by eigenvalue *D*^(^*^α^*^)^(*g*) coefficients as in Equation (52). It is not possible to diagonalize two non-commuting **g**_1_ and **g**_2_ in one basis since numbers (eigenvalues) always commute. If **g**_1_ and **g**_2_ do not commute, their collective resolution must include eigen-*matrix* coefficients *D_m_*,*_n_^α^* involving *nilpotent* (**N**^2^ = **0**) operators **P***_m_*,*_n_^α^*=|*_m_^α^*〉〈*_n_^α^*| as well as idempotent (**I**^2^ = **I**) operators **P***_m_*,*_m_^α^*=| *_m_^α^* 〉〈*_m_^α^*| seen in Equation (52).

Unlike a commutative algebra of *C_n_* idempotents, which are shown in Equation (54) and uniquely defined by Equation (59), a non-Abelian algebra yields a panopoly of equivalent choices of **P** operators that resolve it. The number and types of these **P**’s is uniquely determined by size and structure of certain key commuting sub-algebras. The key to symmetry analysis of quantum physics is to first sort out the operators and algebras that commute from those that do not. It amounts to a kind of symmetry analysis of symmetry and leads to a far greater diversity than is found in commutative Abelian systems.

#### 6.2.1. Sorting Commuting Subalgebras: Rank and Commuting Observables

The *rank ρ*(*G*) of a *G*-algebra is the maximal number of *mutually*-*commuting* operators available by linearly combining the *^o^G* operators **g***_k_* of symmetry group *G. ρ*(*G*) is also the greatest number of orthogonal idempotents **P***^m^* that can resolve the *G*-identity **1** as in Equation (53). (*^o^G* is total number or *order* of *G*. Here *^o^D*_3_ and *^o^C*_6_ both equal 6.)

*C*_6_ rank is obviously equal to its order (*ρ*(*C*_6_) = 6), but the rank of *D*_3_ turns out to be only four (*ρ*(*D*_3_) = 4). As shown below, *D*_3_ can have no more than four **P**-operators that *mutually* commute though there exist quite different sets of them. On the other hand, *D*_3_ has just *three* linearly independent **P***^α^*-operators that commute with *all* of *D*_3_, and there is but *one* invariant set of them just as there is but one set of **P**^(^*^m^*^)^ for *C*_6_ in Equation (59).

Rank is a key quantum concept since it is the total number of commuting observables, the operators that label and define eigenstates. Of primary importance are *G*-*invariant* labeling operators **I***_G_* that commute with *all***g** and not just with other labeling operators. **I***_G_* are uniquely defined within their group *G* and invariant to all **g**. (**gI***_G_***g**^−1^=**I***_G_*) For example, total angular momentum **J**^2^ and e-values *J*(*J* + 1) are *R*(3)-invariant.

Next in importance are labeling operators [**I***_H__n_*_−1_, **I***_H__n_*_−2_, *...,***I***_H_*__1__ ] belonging to nested subgroups of *G*=*H_n_* in a *subgroup chain G*⊃*H_n_*_−1_⊃*H_n_*_−2_⊃*... H*_1_. Multiple choices of chains exists since each subgroup link *H_k_* is not uniquely determined by the *H_k_*_+1_ that contains it, but each **I***_H_*_*_k_*_ is invariant to all possible *H_j_*_≤_*_k_* at level-*k* or below.

For example, the *z*-axial momentum **J***_z_* and its e-values *m_z_* belong to a 2*^nd^* link in chain-*R*(3)⊃*R*(2*_z_*)⊃*C*_6_(*z*). Given *R*(3) there are an infinity of *R*(2) subgroups besides the one for *z*-axis of quantization. **J***_x_* or **J***_y_* are just two of an infinite number of possible alternatives to **J***_z_*. Each *R*(2*_ζ_*) has an infinite number of cyclic *C_n_*(*ζ*) sub-subgroups.

#### 6.2.2. Sorting Commuting Subalgebras: Centrum and Class Invariants

The *centrum κ*(*G*) of a *G*-algebra is the number of *all*-*commuting* operators available by combining **g***_k_*. It is also the number of *G*-*invariant***P***^α^*-operators. Students of group theory know *κ*(*G*) as the number of equivalence *classes* of group *G. D*_3_ elements in Figure 16 are separated into three classes of elements [**1**], [**r**^1^*,***r**^2^], and [**i**_1_*,***i**_2_*,***i**_3_]. (*κ*(*D*_3_) = 3)

Elements in each class are related through transformation **g**_1_=**g***_t_***g**_2_**g***_t_*^−1^ by **g***_t_* in group *G*. Sum *κ***_k_** of *^o^c_k_* elements in **g***_k_*’s class is invariant to **g***_t_* transformation. (It only permutes **g***_k_*-terms in *κ***_k_** thus *κ***_k_** commutes with all **g***_t_* in *G*.)

(72) $$g_{t}\kappa_{k}g_{t}^{-1}=\kappa_{k}\;\text{where}:\kappa_{k}=\sum_{j=1}^{j={}^{\circ}c_{k}}g_{j}=1/{}^{\circ}s_{k}\sum_{t=1}^{t={}^{\circ}G}g_{t}g_{k}g_{t}^{-1}$$

The product table for *D*_3_ class algebra [*κ*_1_ = **1,***κ*_2_ = **r**^1^ + **r**^2^, *κ*_3_ = **i**_1_ + **i**_2_ + **i**_3_] in Equation (73) below follows by inspecting *D*_3_ group product tables in Figure 16 or Equation (68). It is a commutative algebra since each *κ***_j_** commutes with each *κ***_k_** as well as with each **g***_t_*. This guarantees a class algebra has a unique and invariant spectral resolution.

(73) $$\begin{array}{cccccc} 1 & r^{1} & r^{2} & i_{1} & i_{2} & i_{3}\\ r^{2} & 1 & r^{1} & i_{2} & i_{3} & i_{1}\\ r^{1} & r^{2} & 1 & i_{3} & i_{1} & i_{2}\\ i_{1} & i_{2} & i_{3} & 1 & r^{1} & r^{2}\\ i_{2} & i_{3} & i_{1} & r^{2} & 1 & r^{1}\\ i_{3} & i_{1} & i_{2} & r^{1} & r^{2} & 1 \end{array}\to \begin{array}{ccc} \kappa_{1}=1 & \kappa_{2}=r^{1}+r^{2} & \kappa_{3}=i_{1}+i_{2}+i_{3}\\ \kappa_{2} & 2\kappa_{1}+\kappa_{2} & 2\kappa_{3}\\ \kappa_{3} & 2\kappa_{3} & 3\kappa_{1}+3\kappa_{2} \end{array}$$

The first sum in Equation (72) is over the *^o^c_k_* elements in **g***_k_*’s class. (*^o^c_k_* is order of *κ***_k_**.) The second sum is over all *^o^G* group elements. The number of elements **g***_t_* that commute with **g***_k_* is *^o^s_k_*, the order of **g***_k_*’s self-symmetry *s_k_*. Each group operator **g***_k_* has a self-symmetry group consisting of (at least) the identity **1** and powers (**g***_k_*)*^p^* of itself. The order of class-*k* is the (integer) fraction *^o^c_k_*=*^o^G*/*^o^s_k_*.

#### 6.2.3. Resolving All-commuting Class Subalgebra: Centrum=*κ*(*D*_3_) = 3

Spectral resolution gives class-sum operators *κ***_1_**, *κ***_2_**, and *κ***_3_** as combinations of three *D*_3_-invariant **P***^α^*-operators with each of the *κ***_k_** eigenvalues as coefficients. The *κ***_3_** characteristic equation found by Equation (73) gives three **P***^α^* directly.

(74) $$\begin{array}{l} 0={\kappa_{3}}^{3}-9\kappa_{3}=(\kappa_{3}-3\cdot 1)(\kappa_{3}+3\cdot 1)(\kappa_{3}+0\cdot 1)\\ =(\kappa_{3}-3\cdot 1)P^{A_{1}}=(\kappa_{3}+3\cdot 1)P^{A_{2}}=(\kappa_{3}-0\cdot 1)P^{E} \end{array}$$

Standard notation *A*_1_, *A*_2_, and *E* is used for the three invariant idempotents **P***^α^*.

(75) $$\begin{array}{lll} \kappa_{1}=1\cdot P^{A_{1}}+1\cdot P^{A_{2}}+1\cdot P^{E} & P^{A_{1}}=(\kappa_{1}+\kappa_{2}+\kappa_{3})/6 & =(1+r^{1}+r^{2}+i_{1}+i_{2}+i_{3})/6\\ \kappa_{2}=2\cdot P^{A_{1}}-2\cdot P^{A_{2}}-1\cdot P^{E} & P^{A_{2}}=(\kappa_{1}+\kappa_{2}-\kappa_{3})/6 & =(1+r^{1}+r^{2}-i_{1}-i_{2}-i_{3})/6\\ \kappa_{3}=3\cdot P^{A_{1}}-3\cdot P^{A_{2}}+0\cdot P^{E} & P^{E}=(2\kappa_{1}-\kappa_{2})/3 & =(21-r^{1}-r^{2})/3 \end{array}$$

Traces of *D*_3_ matrices (**g***_k_*) in Equation (69a) are zero excepting *Trace*(**1**) = 6. Traces of (**P***^α^*) then follow.

(76) $$\begin{array}{ccc} traceP^{A_{1}}=1, & traceP^{A_{2}}=1, & traceP^{E}=4 \end{array}$$

This means (**P***^A^*^_1_^) and (**P***^A^*^_2_^) are each 1-*by*-1 projectors while (**P***^E^*) splits into two 2-*by*-2 projectors. The latter splitting is not uniquely defined until subgroup chain *D*_3_⊃*C*_3_ or a particular *D*_3_⊃*C*_2_ chain is chosen, but relations in Equation (75) are invariant and unique. The *κ_k_* coefficients inside parentheses of **P***^α^* expansion give the *D*_3_*character* table for traces of irreducible representations (irreps). Irrep *dimension ℓ^α^* is trace of the *α^th^*-irrep of identity **g**_1_ = **1**.

(77) $$\begin{array}{cccc} D_{3} & \kappa_{1} & \kappa_{2} & \kappa_{3}\\ A_{1} & 1 & 1 & 1\\ A_{2} & 1 & 1 & -1\\ E & 2 & -1 & 0 \end{array}\;\;\;\chi_{k}^{\alpha }=TraceD^{\alpha }(g_{k}),\;\;\;\ell^{\alpha }=\chi_{1}^{\alpha }=TraceD^{\alpha }(1)$$

#### 6.2.4. Resolving Maximal Mutually Commuting Subalgebra: rank = *ρ*(*D*_3_) = 4

Completing resolution of *D*_3_ uses a product of two completeness relations, the resolution of class identity *κ*_1_ = **1** in Equation (75) with the identity resolution of a *D*_3_ subgroup *C*_3_ = [**1***,***r**^1^*,***r**^2^] or else *C*_2_ = [**1***,***i**_3_]. In either case invariant **P***_E_* splits but **P***^A^*^_1_^ and **P***^A^*^_2_^ do not. In Equation (78)**P***^E^* is split by *C*_2_ into plane-polarizing projectors **P***_x,x_^E^* + **P***_y,y_^E^* = **P**_0_2_0_2__*^E^* + **P**_1_2_1_2__*^E^*.

(78) $$\begin{array}{l} \left[\begin{array}{c} D_{3}\;\left(\begin{array}{l} class\;algebra\\ completeness \end{array}\right)\\ 1=P^{A_{1}}+P^{A_{2}}+P^{E} \end{array}\right]\cdot \left[\begin{array}{c} C_{2}\;\left(\begin{array}{l} subgroup\\ completeness \end{array}\right)\\ 1=P^{0_{2}}+P^{1_{2}} \end{array}\right]=\left[\begin{array}{c} D_{3}\;\left(\begin{array}{l} group\\ completeness \end{array}\right)\\ 1=P_{0_{2}0_{2}}^{A_{1}}+P_{1_{2}1_{2}}^{A_{2}}+P_{0_{2}0_{2}}^{E}+P_{1_{2}1_{2}}^{E} \end{array}\right]\\ \text{where}:\;\;\;\begin{array}{l} P^{A_{1}}=P_{0_{2}0_{2}}^{A_{1}}=(1+r^{1}+r^{2}+i_{1}+i_{2}+i_{3})/6=P^{A_{1}}P^{0_{2}}\\ P^{A_{2}}=P_{1_{2}1_{2}}^{A_{2}}=(1+r^{1}+r^{2}-i_{1}-i_{2}-i_{3})/6=P^{A_{2}}P^{1_{2}}\\ P_{x,x}^{E}=P_{0_{2}0_{2}}^{E}=(21-r^{1}-r^{2}-i_{1}-i_{2}+2i_{3})/6=P^{E}P^{0_{2}}\\ P_{y,y}^{E}=P_{1_{2}1_{2}}^{E}=(21-r^{1}-r^{2}+i_{1}+i_{2}-2i_{3})/6=P^{E}P^{1_{2}} \end{array}\;\;\;(\text{All other }P^{\alpha }P^{m_{2}}=0) \end{array}$$

In Equation (79)**P***^E^* is split by *C*_3_ into Right and Left circular-polarized projectors **P***_R,R_^E^* +**P***_L,L_^E^* =**P**_1_3_1_3__*^E^*+**P**_2_3_2_3__*^E^*.

(79) $$\begin{array}{l} \left[\begin{array}{c} D_{3}\;\left(\begin{array}{l} class\;algebra\\ completeness \end{array}\right)\\ 1=P^{A_{1}}+P^{A_{2}}+P^{E} \end{array}\right]\cdot \left[\begin{array}{c} C_{3}\;\left(\begin{array}{l} subgroup\\ completeness \end{array}\right)\\ 1=P^{0_{3}}+P^{1_{3}}+P^{2_{3}} \end{array}\right]=\left[\begin{array}{c} D_{3}\;\left(\begin{array}{l} group\\ completeness \end{array}\right)\\ 1=P_{0_{3}0_{3}}^{A_{1}}+P_{0_{3}0_{3}}^{A_{2}}+P_{1_{3}1_{3}}^{E}+P_{2_{3}2_{3}}^{E} \end{array}\right]\\ \text{where}:\;\;\;\begin{array}{l} P^{A_{1}}=P_{0_{3}0_{3}}^{A_{1}}=(1+r^{1}+r^{2}+i_{1}+i_{2}+i_{3})/6=P^{A_{1}}P^{0_{3}}\\ P^{A_{2}}=P_{0_{3}0_{3}}^{A_{2}}=(1+r^{1}+r^{2}-i_{1}-i_{2}-i_{3})/6=P^{A_{2}}P^{0_{3}}\\ P_{R,R}^{E}=P_{1_{3}1_{3}}^{E}=(1+ɛ\;r^{1}+{ɛ}^{*}r^{2})/3=P^{E}P^{1_{3}}\\ P_{L,L}^{E}=P_{2_{3}2_{3}}^{E}=(1+{ɛ}^{*}r^{1}+ɛ\;r^{2})/3=P^{E}P^{2_{3}} \end{array}\;\;\;(\text{All other }P^{\alpha }P^{m_{3}}=0)\;ɛ={\text{e}}^{i2\pi /3} \end{array}$$

In Equations (78) and (79), neither **P***^A^*^_1_^ nor **P***^A^*^_2_^ split or change except to acquire some *C*_2_ or *C*_3_ labels. The total number (four) of irreducible idempotents after either complete splitting is the same group rank noted before: *ρ*(*D*_3_)=4. But, the *RL*-circularly polarized pairs **P***_R,R_^E^* and **P***_L,L_^E^* split-out by *C*_3_=[**1***,***r**^1^*,***r**^2^] differ from the linear *xy*-polarized pairs **P***_x,x_^E^* and **P***_y,y_^E^* split-out by *C*_2_=[**1***,***i**_3_]. **P***_x,x_^E^* and **P***_y,y_^E^* are, respectively, parallel (symmetric **i**_3_**P***_x_^E^* =+**P***_x_^E^)* and anti-parallel (anti-symmetric **i**_3_**P***_y_^E^* =−**P***_y_^E^)* to *x*-axial 180*^o^* rotation **i**_3_ in Figure 16 and will be used in examples.

#### 6.2.5. Final Resolutions of Non-Commuting Algebra: *^o^*(*D*_3_) = 6

Mutually commuting algebras resolve into (**I**_2_ = **I**) operators $P_{m,m}^{\alpha }=|\begin{array}{c} \alpha \\ m \end{array}\rangle \langle \begin{array}{c} \alpha \\ m \end{array}|$ that sum to identity operator **1**. They are split using the “one-equals-one-times-one” (**1**=**1**·**1**) trick in Equations (78) and (79).

Non-commuting algebras resolve into idempotents and nilpotent (**N**^2^ = **0**) operators $P_{m,n}^{\alpha }=|\begin{array}{c} \alpha \\ m \end{array}\rangle \langle \begin{array}{c} \alpha \\ n \end{array}|$ that are split out using the following “operator-equals-one-times-operator-times-one” (**g**=**1**·**g**·**1**) trick. It is only necessary that **1** be resolved into rank-number *ρ* of irreducible idempotents as in Equation (78) or (79). (Here *ρ*(*D*_3_) = 4.)

(80) $$g=1\cdot g\cdot 1=(P_{x,x}^{A_{1}}+P_{y,y}^{A_{2}}+P_{x,x}^{E}+P_{y,y}^{E})\cdot g\cdot (P_{x,x}^{A_{1}}+P_{y,y}^{A_{2}}+P_{x,x}^{E}+P_{y,y}^{E})$$

The product in Equation (80) could have sixteen terms, but only six survive due to idempotent orthogonality **P***_j,j_^α^***P***_k,k_^β^* = *δ^α,β^δ_j,k_***P***_j,j_^α^,* and the fact that both **P***^A^*^_1_^ and **P***^A^*^_2_^ remain invariant and commute with all **P***_j,j_^α^* and all **g**.

(81) $$g=P^{A_{1}}\cdot g\cdot P^{A_{1}}+P^{A_{2}}\cdot g\cdot P^{A_{2}}+P_{x,x}^{E}\cdot g\cdot P_{x,x}^{E}+P_{x,x}^{E}\cdot g\cdot P_{y,y}^{E}+P_{y,y}^{E}\cdot g\cdot P_{x,x}^{E}+P_{y,y}^{E}\cdot g\cdot P_{y,y}^{E}$$

This reduces to a non-Abelian spectral resolution of *D*_3_ that generalizes resolution Equation (52) of Abelian *C*_6_ and includes two nilpotent projectors **P***_j,k_^α^* multiplied by off-diagonal irrep matrix components *D_j,k_^α^* as well as the four idempotents **P***_j,j_^α^* with their diagonal irrep matrix coefficients *D_j,j_^α^* that are not altogether unlike the *D*^(^*^m^*^)^(**r***^p^*)**P**^(^*^m^*^)^ terms in Equation (52). ( Now *X* has matrix indices (*X_j,k_*).)

(82a) $$g=\sum_{irreps\;(\alpha )}\sum_{j=1}^{\ell^{\alpha }}\sum_{k=1}^{\ell^{\alpha }}D_{j,k}^{\alpha }(g)P_{j,k}^{\alpha }$$

(82b) $$\begin{array}{l} g=D^{A_{1}}(g)P^{A_{1}}+D^{A_{2}}(g)P^{A_{2}}+D_{x,x}^{E}(g)P_{x,x}^{E}+D_{x,y}^{E}(g)P_{x,y}^{E}+D_{y,x}^{E}(g)P_{y,x}^{E}+D_{y,y}^{E}(g)P_{y,y}^{E}\\ \text{where}:P_{\text{j},\text{j}}^{\alpha }\cdot g\cdot P_{\text{j},\text{j}}^{\alpha }={\text{D}}_{\text{j},\text{j}}^{\alpha }(\text{g})P_{\text{j},\text{j}}^{\alpha }\;\;\;P_{\text{j},\text{j}}^{\alpha }\cdot g\cdot P_{\text{k},\text{k}}^{\alpha }={\text{D}}_{\text{j},\text{k}}^{\alpha }(\text{g})P_{\text{j},\text{k}}^{\alpha } \end{array}$$

Terms (1*/n*)*D*^(^*^m^*^)*^(**r***^p^*)**r***^p^* in Equation (59) of **P**^(^*^m^*^)^ of *C_n_* in Equation (52) generalize here to **P***_j,k_^α^* and invert Equation (82a) to Equation (83).

(83) $$P_{j,k}^{\alpha }=(\ell^{\alpha }/{}^{\circ}G)\sum_{g=1}^{{}^{\circ}G}D_{j,k}^{\alpha *}(g)g$$

*D*_3_ resolution in Equation (82b) has two irreps *D^A^*^_1_^ and *D^A^*^_2_^ of dimension *ℓ^A^*^_1_^=1=*ℓ^A^*^_2_^ and a third irrep *D^E^* of dimension *ℓ^E^*=2 as noted in the first column of the character array in Equation (77). The irrep dimensions are related to the centrum *κ*(*D*_3_)=3, rank *ρ*(*D*_3_)=4, and order *^o^D*_3_=6. The following power sums of *ℓ^α^* apply to any finite group *G*.

(84) $$\begin{array}{ll} G-centrum: & \kappa (G)=\sum_{irrep(\alpha )}{\left(\ell^{\alpha }\right)}^{0}=Number\;of\;classes,invariants,or\;irrep\;types\\ G-rank: & \rho (G)=\sum_{irrep(\alpha )}{\left(\ell^{\alpha }\right)}^{1}=Number\;of\;mutually\;commuting\;observables\\ G-order: & {}^{\circ}(G)=\sum_{irrep(\alpha )}{\left(\ell^{\alpha }\right)}^{2}=Number\;of\;symmetry\;operators \end{array}$$

### 6.3. Spectral Resolution of Dual Groups D_3_ and D̄_3_

Spectral resolution shown in Equations (82a) and (83) of non-Abelian group *G* reduce **g**·**h**-product tables in Equation (68) to *P*-projector algebra.

(85) $$P_{jk}^{\alpha }P_{j^{\prime }k^{\prime }}^{\beta }=\delta^{\alpha \beta }\delta_{kj^{\prime }}P_{jk^{\prime }}^{\alpha }$$

Product tables in Equation (86) for *D*_3_ projectors **P***_jk_^α^* generalize the *C*_6_ idempotent table in Equation (54). Non-commutativity entails a pair of tables like the **g**^†^**g** form and **gg**^†^-forms in Equation (68) for “lab” **g** and “body” **ḡ** operators. Tables in Equation (68) differ by switching conjugate pair **r**^1^ and **r**^2^ on side and top.(**r**^1†^ = **r**^2^) The rest are self conjugate. (**i**_1_^†^=**i**_1_, *etc*.) Similarly, tables in Equation (86) differ by switching conjugate nilpotent pair **P***_xy_^E^* and **P***_yx_^E^*. (**P***_xy_^E^*^†^ =**P***_yx_^E^*) The rest are self-conjugate. (**P***_jj_^α^*^†^ =**P***_jj_^α^*)

(86) $$\begin{array}{l} \begin{array}{ccccccc} \begin{array}{l} p^{†}p\\ form \end{array} & P_{xx}^{A_{1}} & P_{yy}^{A_{2}} & P_{xx}^{E_{1}} & P_{xy}^{E_{1}} & P_{yx}^{E_{1}} & P_{yy}^{E_{1}}\\ P_{xx}^{A_{1}} & P_{xx}^{A_{1}} & \cdot & \cdot & \cdot & \cdot & \cdot \\ P_{yy}^{A_{2}} & \cdot & P_{yy}^{A_{2}} & \cdot & \cdot & \cdot & \cdot \\ P_{xx}^{E_{1}} & \cdot & \cdot & P_{xx}^{E_{1}} & P_{xy}^{E_{1}} & \cdot & \cdot \\ P_{yx}^{E_{1}} & \cdot & \cdot & P_{yx}^{E_{1}} & P_{yy}^{E_{1}} & \cdot & \cdot \\ P_{xy}^{E_{1}} & \cdot & \cdot & \cdot & \cdot & P_{xx}^{E_{1}} & P_{xy}^{E_{1}}\\ P_{yy}^{E_{1}} & \cdot & \cdot & \cdot & \cdot & P_{yx}^{E_{1}} & P_{yy}^{E_{1}} \end{array}\\ \begin{array}{ccccccc} \begin{array}{l} pp^{†}\\ form \end{array} & P_{xx}^{A_{1}} & P_{yy}^{A_{2}} & P_{xx}^{E_{1}} & P_{yx}^{E_{1}} & P_{xy}^{E_{1}} & P_{yy}^{E_{1}}\\ P_{xx}^{A_{1}} & P_{xx}^{A_{1}} & \cdot & \cdot & \cdot & \cdot & \cdot \\ P_{yy}^{A_{2}} & \cdot & P_{yy}^{A_{2}} & \cdot & \cdot & \cdot & \cdot \\ P_{xx}^{E_{1}} & \cdot & \cdot & P_{xx}^{E_{1}} & \cdot & P_{xy}^{E_{1}} & \cdot \\ P_{xy}^{E_{1}} & \cdot & \cdot & \cdot & P_{xx}^{E_{1}} & \cdot & P_{xy}^{E_{1}}\\ P_{yx}^{E_{1}} & \cdot & \cdot & P_{yx}^{E_{1}} & \cdot & P_{yy}^{E_{1}} & \cdot \\ P_{yy}^{E_{1}} & \cdot & \cdot & \cdot & P_{yx}^{E_{1}} & \cdot & P_{yy}^{E_{1}} \end{array} \end{array}$$

The **p**^†^**p** and **pp**^†^ tables in Equation (86) give commuting representations of projector **P***_jk_^α^* just as **g**^†^**g** and **gg**^†^ tables in Equation (68) give commuting (**g**)*_G_*-matrices in Equation (69a). Wherever **P***_jk_^α^* appears in a table, a “1” is put in its (**p**)-matrix. Putting “*D_jk_^α^* (*g*)” at each **P***_jk_^α^* spot instead gives the following **p**^†^**p**-representation (**g**)*_P_* of **g** since it is a sum of *D_jk_^α^* (*g*)**P***_jk_^α^* in Equation (82a).

(87) $$\begin{array}{l} {\left(g\right)}_{P}=T{\left(g\right)}_{G}T^{†}=\\ \left(\begin{array}{cccccc} |P_{xx}^{A_{1}}\rangle & |P_{yy}^{A_{2}}\rangle & |P_{xx}^{E_{1}}\rangle & |P_{yx}^{E_{1}}\rangle & |P_{xy}^{E_{1}}\rangle & |P_{yy}^{E_{1}}\rangle \\ D^{A_{1}}(g) & \cdot & \cdot & \cdot & \cdot & \cdot \\ \cdot & D^{A_{2}}(g) & \cdot & \cdot & \cdot & \cdot \\ \cdot & \cdot & D_{xx}^{E_{1}}(g) & D_{xy}^{E_{1}}(g) & \cdot & \cdot \\ \cdot & \cdot & D_{yx}^{E_{1}}(g) & D_{yy}^{E_{1}}(g) & \cdot & \cdot \\ \cdot & \cdot & \cdot & \cdot & D_{xx}^{E_{1}}(g) & D_{xy}^{E_{1}}(g)\\ \cdot & \cdot & \cdot & \cdot & D_{yx}^{E_{1}}(g) & D_{yy}^{E_{1}}(g) \end{array}\right) \end{array}$$

Conjugate **pp**^†^-representation (**ḡ**)*_P_* of **ḡ** has complex conjugate “D_jk_^α^^*^ (*g*)” put at each **P***_jk_^α^* spot. The matrices in Equations (87) and (88) are transformations (**g**)*_P_* = *T*(**g**)*_G_T*^†^ and (**ḡ**)*_P_* = *T*(**ḡ**)*_G_T*^†^ of the respective matrices in Equations (69a) and (69b) by transformation *T* composed of *D_jk_^α^* (*g*) components. The *C*_6_ analogy is Fourier transform Equation (51) from Equation (48) to Equation (54).

(88) $$\begin{array}{l} {\left(\bar{g}\right)}_{P}=T{\left(\bar{g}\right)}_{G}T^{†}=\\ \left(\begin{array}{cccccc} |P_{xx}^{A_{1}}\rangle & |P_{yy}^{A_{2}}\rangle & |P_{xx}^{E_{1}}\rangle & |P_{yx}^{E_{1}}\rangle & |P_{xy}^{E_{1}}\rangle & |P_{yy}^{E_{1}}\rangle \\ D^{A_{1}*}(g) & \cdot & \cdot & \cdot & \cdot & \cdot \\ \cdot & D^{A_{2}*}(g) & \cdot & \cdot & \cdot & \cdot \\ \cdot & \cdot & D_{xx}^{E_{1}*}(g) & \cdot & D_{xy}^{E_{1}*}(g) & \cdot \\ \cdot & \cdot & \cdot & D_{xx}^{E_{1}*}(g) & \cdot & D_{xy}^{E_{1}*}(g)\\ \cdot & \cdot & D_{yx}^{E_{1}*}(g) & \cdot & D_{yy}^{E_{1}*}(g) & \cdot \\ \cdot & \cdot & \cdot & D_{yx}^{E_{1}*}(g) & \cdot & D_{yy}^{E_{1}*}(g) \end{array}\right) \end{array}$$

Matrices ...(**r̄**^2^)*_P_,* (**ī**_1_)*_P_, ...* defined by Equation (88) commute with every ...(**r**^2^)*_P_,* (**i**_1_)*_P_, ...* defined by Equation (87) while each represents identical *non*-commutative *D*_3_ product tables in Equation (68). Both use real [*x, y*]-based **i**_3_-diagonal irreps *D_jk_^α^* (*g*) given below.

(89) $$\begin{array}{ccccccc} g= & 1 & r & r^{2} & i_{1} & i_{2} & i_{3}\\ D^{A_{1}}(g)= & 1 & 1 & 1 & 1 & 1 & 1\\ D^{A_{2}}(g)= & 1 & 1 & 1 & -1 & -1 & -1\\ D_{\begin{array}{cc} xx & xy\\ yx & yy \end{array}}^{E}(g)= & \left(\begin{array}{cc} 1 & \cdot \\ \cdot & 1 \end{array}\right) & \left(\begin{array}{cc} \frac{-1}{2} & \frac{-\sqrt{3}}{2}\\ \frac{\sqrt{3}}{2} & \frac{-1}{2} \end{array}\right) & \left(\begin{array}{cc} \frac{-1}{2} & \frac{\sqrt{3}}{2}\\ \frac{-\sqrt{3}}{2} & \frac{-1}{2} \end{array}\right) & \left(\begin{array}{cc} \frac{-1}{2} & \frac{-\sqrt{3}}{2}\\ \frac{-\sqrt{3}}{2} & \frac{1}{2} \end{array}\right) & \left(\begin{array}{cc} \frac{-1}{2} & \frac{\sqrt{3}}{2}\\ \frac{\sqrt{3}}{2} & \frac{1}{2} \end{array}\right) & \left(\begin{array}{cc} 1 & \cdot \\ \cdot & -1 \end{array}\right) \end{array}$$

Appendix-A describes elementary derivation and visualization of *D_jk_^α^* (*g*) and their projectors **P***_jk_^α^* (*g*).

### 6.4. Spectral Resolution of D_3_ Hamiltonian

**Figure 17:**
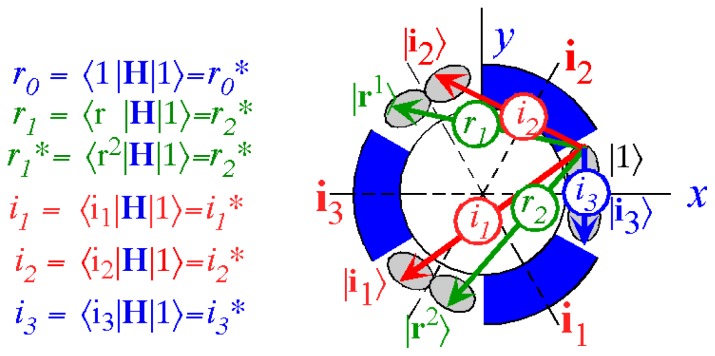
*D*_3_-operator defined states and tunneling paths.

Hamiltonian **H**-matrix in Equation (71) has six parameters [*r*_0_*, r*_1_*, r*_2_*, i*_1_*, i*_2_*, i*_3_] or coefficients of its expansion Equation (70) in terms of intrinsic *D̄*_3_ operators [**1** = **r̄**^0^*,***r̄**^1^*,***r̄**^2^*,***ī**_1_*,***ī**_2_*,***ī**_3_]. The parameters are indicated in Figure 17 by tunneling paths between the first *D*_3_ base state |1〉 and other *D*_3_-defined base states |**g**〉 = **g**|1〉 representing potential minima.

The resolution of **H**-matrix then follows that of **ḡ** and (**ḡ**)*_P_* -matrices. Any reduction of all (**ḡ**)*_P_* -matrices, such as the [*x, y*]-reduction in Equation (88), also reduces the (**H**)*_P_* -matrix accordingly. Row-**1** of (**H***_P_)* in Equation (71) has all six parameters.

(90) $$H_{ab}^{\alpha }=\sum_{g=1}^{{}^{\circ}G}\langle 1|H|g\rangle D_{ab}^{\alpha *}(g)=\sum_{g=1}^{{}^{\circ}G}r_{g}D_{ab}^{\alpha *}(g)$$

If the *P*-nilpotent pair are switched to ... **P***_xy_^E^*, **P***_yx_^E^*.., then (**H**)*_P_* and all (**ḡ**)*_P_* (instead of all (**g**)*_P_* as in Equation (87)) are diagonal with eigenvalues *H^A^*^_1_^ and *H^A^*^_2_^ or block-diagonal with a pair of identical 2-*by*-2 *H^E^*-blocks.

(91) $${\left(H\right)}_{P}=\bar{T}{\left(H\right)}_{G}{\bar{T}}^{†}=\left(\begin{array}{cccccc} |P_{xx}^{A_{1}}\rangle & |P_{yy}^{A_{2}}\rangle & |P_{xx}^{E_{1}}\rangle & |P_{xy}^{E_{1}}\rangle & |P_{yx}^{E_{1}}\rangle & |P_{yy}^{E_{1}}\rangle \\ H^{A_{1}} & \cdot & \cdot & \cdot & \cdot & \cdot \\ \cdot & H^{A_{2}} & \cdot & \cdot & \cdot & \cdot \\ \cdot & \cdot & H_{xx}^{E} & H_{xy}^{E} & \cdot & \cdot \\ \cdot & \cdot & H_{yx}^{E} & H_{yy}^{E} & \cdot & \cdot \\ \cdot & \cdot & \cdot & \cdot & H_{xx}^{E} & H_{xy}^{E}\\ \cdot & \cdot & \cdot & \cdot & H_{yx}^{E} & H_{yy}^{E} \end{array}\right)$$

The *H*-block matrix components follow by combining Equation (89) with Equation (90).

(92) $$\begin{array}{l} H^{A_{1}}=r_{0}D^{A_{1}*}(1)+r_{1}D^{A_{1}*}(r^{1})+r_{1}^{*}D^{A_{1}*}(r^{2})+i_{1}D^{A_{1}*}(i_{1})+i_{2}D^{A_{1}*}(i_{2})+i_{3}D^{A_{1}*}(i_{3})=r_{0}+r_{1}+r_{1}^{*}+i_{1}+i_{2}+i_{3}\\ H^{A_{2}}=r_{0}D^{A_{2}*}(1)+r_{1}D^{A_{2}*}(r^{1})+r_{1}^{*}D^{A_{2}*}(r^{2})+i_{1}D^{A_{2}*}(i_{1})+i_{2}D^{A_{2}*}(i_{2})+i_{3}D^{A_{2}*}(i_{3})=r_{0}+r_{1}+r_{1}^{*}-i_{1}-i_{2}-i_{3}\\ H_{xx}^{E}=r_{0}D_{xx}^{E*}(1)+r_{1}D_{xx}^{E*}(r^{1})+r_{1}^{*}D_{xx}^{E*}(r^{2})+i_{1}D_{xx}^{E*}(i_{1})+i_{2}D_{xx}^{E*}(i_{2})+i_{3}D_{xx}^{E*}(i_{3})\\ =(2r_{0}-r_{1}-r_{1}^{*}-i_{1}-i_{2}+2i_{3})/2\\ H_{xy}^{E}=r_{0}D_{xy}^{E*}(1)+r_{1}D_{xy}^{E*}(r^{1})+r_{1}^{*}D_{xy}^{E*}(r^{2})+i_{1}D_{xy}^{E*}(i_{1})+i_{2}D_{xy}^{E*}(i_{2})+i_{3}D_{xy}^{E*}(i_{3})\\ =\sqrt{3}(-r_{1}+r_{1}^{*}-i_{1}+i_{2})/2=H_{yx}^{E*}\\ H_{yy}^{E}=r_{0}D_{yy}^{E*}(1)+r_{1}D_{yy}^{E*}(r^{1})+r_{1}^{*}D_{yy}^{E*}(r^{2})+i_{1}D_{yy}^{E*}(i_{1})+i_{2}D_{yy}^{E*}(i_{2})+i_{3}D_{yy}^{E*}(i_{3})\\ =(2r_{0}-r_{1}-r_{1}^{*}+i_{1}+i_{2}-2i_{3})/2 \end{array}$$

Irrep-dimension *ℓ^E^* = 2 implies (at least) 2-fold degenerate *E*-level since eigenvalues of identical *H^E^*-blocks must also be identical, but only certain parameter values give diagonal *H^E^*-blocks in Equation (92), *i.e*., real *r*_1_ = *r*_2_^*^ and equal *i*_1_ = *i*_2_.

(93) $$\begin{array}{l} \left(\begin{array}{cc} H_{xx}^{E} & H_{xy}^{E}\\ H_{yx}^{E} & H_{yy}^{E} \end{array}\right)=\frac{1}{2}\left(\begin{array}{cc} 2r_{0}-r_{1}-r_{1}^{*}-i_{1}-i_{2}+2i_{3} & \sqrt{3}(-r_{1}+r_{1}^{*}-i_{1}+i_{2})\\ \sqrt{3}(-r_{1}^{*}+r_{1}-i_{1}+i_{2}) & 2r_{0}-r_{1}-r_{1}^{*}+i_{1}+i_{2}-2i_{3} \end{array}\right)\\ ={\left(\begin{array}{cc} r_{0}-r_{1}-i_{12}+i_{3} & 0\\ 0 & r_{0}-r_{1}+i_{12}-i_{3} \end{array}\right)}_{\text{For}:r_{1}=r_{1}^{*}\;\text{and}:i_{1}=i_{12}=i_{2}} \end{array}$$

These are the values that respect the local *D*_3_ ⊃ *C*_2_ [**1***,***i**_3_] subgroup chain symmetry that gave (*x, y*)-plane polarized splitting in Equation (78). This is broken by a complex *r*_1_ or by unequal *i*_1_ and *i*_2_. Complex *r*_1_ = |*r*|*e^iφ^* gives rise to complex rotating-wave eigenstates similar to ones in Figure 15 but, unlike that ZB1 model, cannot split *E*-degeneracy. Unequal *i*_1_ and *i*_2_ shift standing-wave nodes but cannot split *E*-doublets either. *E*-levels may split if **H** contains *external* or *lab*-based operators **g** in addition to its *internal* or *body*-based **ḡ**, but it thereby loses its *D*_3_ symmetry.

### 6.5. Global-Lab-Relative G versus Local-Body-Relative Ḡ Base State Definition

Non-Abelian symmetry analysis in general, and the present example of *D*_3_ resolution in particular, involves a dual-group relativity between an *extrinsic* or *global* “lab-based” group *G*=*D*_3_ on one hand, and an *intrinsic* or *local* “body-based” group *Ḡ* = *D̄*_3_ on the other hand. Each **ḡ** in *Ḡ* commutes with each **g** in *G*.

In the present example, the *global* “lab-based” group *G*=*D*_3_=[**1***,***r**^1^*,***r**^2^*,***i**_1_*,***i**_2_*,***i**_3_] labels equivalent locations in a potential or lab-based field and is a reference frame for an excitation wave or “body” occupying lab locations.

On the other hand, the *local* “bod-based” group *Ḡ*=*D̄*_3_=[**1***,***r̄**^1^*,***r̄**^2^*,***ī**_1_*,***ī**_2_*,***ī**_3_] regards the excitation wave as a reference frame to define relative location of the potential or laboratory field.

Quantum waves provide the most precise space-time reference frames that are possible in any situation due to the ultra-sensitive nature of wave interferometry. This is the case for optical coherent waves or electronic and nuclear matter waves. The latter derive their space-time symmetry properties from the former, and these are deep classical and quantum mechanical rules of engagement for currently accepted Hamiltonian quantum theory.

Interference of two waves depends only on *relative* position as reflected in the following equivalent definitions of base kets for waves in a *D*_3_ potential of Figure 16 with six localized wave bases [|**1**〉*,* |**r**^1^〉*,* |**r**^2^〉*,* |**i**_1_〉*,* |**i**_2_〉*,* |**i**_3_〉] in Figure 17. (We call this the “Mock-Mach Principle” of wave relativity.)

(94) $$|g_{k}\rangle =g_{k}|1\rangle ={\bar{g}}_{k}^{-1}|1\rangle$$

Key to this definition is the independence and *mutual commutation* of dual sets Equation (69a) and (69b).

(95) $$g_{j}{\bar{g}}_{k}={\bar{g}}_{k}g_{j}$$

**Figure 18:**
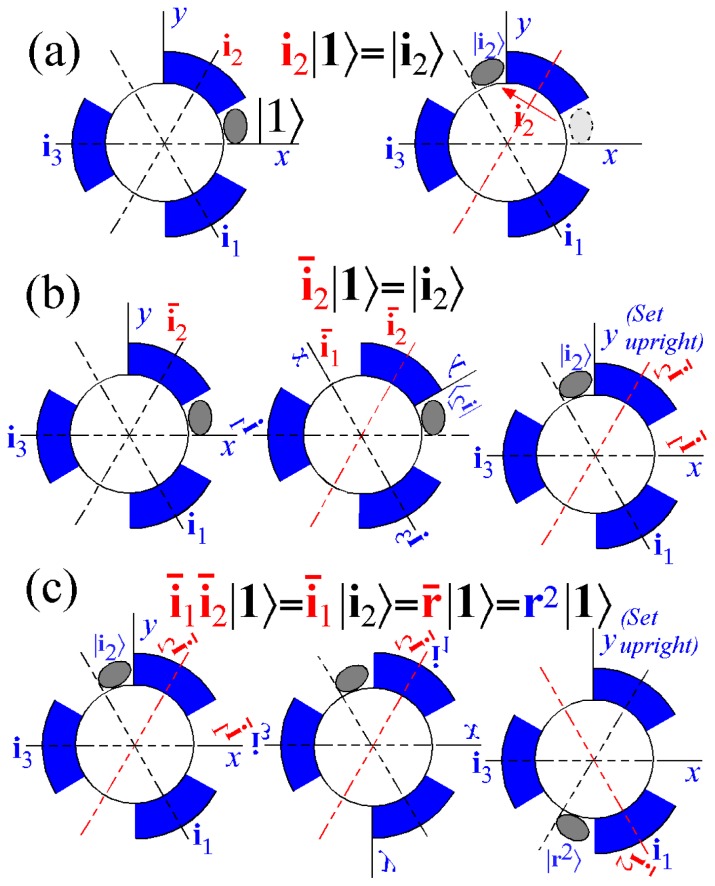
*D*_3_-operators compared (**a**) Global **i**_2_; (**b**) Local **ī**_2_; (**c**) **ī**_2_ followed by **ī**_1_.

Neither relation makes sense if we were to equate **g***_k_* with **ḡ***_k_*^−1^. The effect of **g***_k_* is equal to that of **ḡ***_k_*^−1^*only* when acting on the origin-state |**1**〉. The action of global **i**_2_ in Figure 18a is compared with local**ī**_2_ in Figure 18b that gives the same *relative* position of wave and wells. In Figure 18c product **ī**_1_**ī**_2_ = **r̄** has the same action as **i**_2_**i**_1_=**r**^−1^=**r**^2^ on |**1**〉.

Different points of view show how “body” **ḡ** operations relate to the “lab” **g**. Starting from state |**1**〉, **r̄**^1^ = **r̄** rotates lab potential clockwise (−120*^o^*) in a view where the body “stays put”. The body wave ends up in the same well as it would if, instead, the body rotates counter-clockwise (+120*^o^*) by **r**=**r**^1^ in a lab frame that “stays put.”

In a lab view, effects of body operation **ḡ***_k_* and lab operation **g***_k_*^−1^ on |**1**〉 are the same except that **ḡ***_k_*^−1^ also moves each body operation **ḡ***_j_* in the same way to **ḡ***_k_***ḡ***_j_***ḡ***_k_*^−1^. The lab view of a lab operation **g***_k_* does not see any of lab **g***_j_* axes change location. The following generalization of lab-body relativity relation Equation (94) using Equation (95) shows how **ḡ***_j_* affects arbitrary |**g***_k_*〉.

(96) $$\begin{array}{l} {\bar{g}}_{j}^{-1}|g_{k}\rangle ={\bar{g}}_{j}^{-1}g_{k}|1\rangle =g_{k}{\bar{g}}_{j}^{-1}|1\rangle \\ =g_{k}g_{j}|1\rangle =g_{k}g_{j}g_{k}^{-1}g_{k}|1\rangle =g_{k}g_{j}g_{k}^{-1}|g_{k}\rangle \end{array}$$

### 6.6. Global versus Local Eigenstate Symmetry

Applying projector **P***_jk_^α^* in Equation (83) to origin ket |**1**〉 gives a local-global symmetry-defined ket |*_jk_^α^* 〉.

(97) $${|}_{jk}^{\alpha }\rangle =P_{jk}^{\alpha }|1\rangle \sqrt{{}^{\circ}G/\ell^{\alpha }}=\sqrt{\ell^{\alpha }/{}^{\circ}G}\sum_{g=1}^{{}^{\circ}G}D_{j,k}^{\alpha *}(g)|g\rangle$$

The norm-factor *N*=*^o^G*/*ℓ^α^* is a non-Abelian generalization of the integral norm *N* for Abelian *C_N_* eigenket projection in Equation (61). Interestingly, the non-Abelian norm is also an integer since irrep dimension *ℓ^α^* is always a factor of its group’s order *^o^G*.

A non-Abelian projection ket in Equation (97) has two independent symmetry labels *j* and *k* belonging to global-lab symmetry operators **g** and local-body operators **ḡ**, respectively. Application of **g**-resolution Equation (82a) to ket Equation (97) is reduced by **P**-product rules in Equation (85) to the following global transformation.

(98) $$\begin{array}{l} g{|}_{jk}^{\alpha }\rangle =gP_{jk}^{\alpha }|1\rangle \;\sqrt{N}\\ =\sum_{j^{\prime }=1}^{\ell^{\alpha }}\sum_{k^{\prime }=1}^{\ell^{\alpha }}D_{j^{\prime }k^{\prime }}^{\mu }(g)\;P_{j^{\prime }k^{\prime }}^{\alpha }P_{jk}^{\alpha }|1\rangle \;\sqrt{N}=\sum_{j^{\prime }=1}^{\ell^{\alpha }}D_{j^{\prime }j}^{\alpha }(g)\;P_{j^{\prime }k}^{\alpha }|1\rangle \;\sqrt{N}\\ =\sum_{j^{\prime }=1}^{\ell^{\alpha }}D_{j^{\prime }j}^{\alpha }(g){|}_{j^{\prime }k}^{\alpha }\rangle \end{array}$$

The corresponding local operator **ḡ** first commutes through **P***_jk_^α^* according to Equation (95) and is converted by Equation (94) to inverse global **g**_−_**_1_** on the right of **P***_jk_^α^* using Equation (82a) again. Finally, unitary irreps *D_α_*(*g*^−1^) = *D^α^*^†^(*g*) are assumed.

(99) $$\begin{array}{l} \bar{g}{|}_{jk}^{\alpha }\rangle =\bar{g}P_{jk}^{\alpha }|1\rangle \;\sqrt{N}=P_{jk}^{\alpha }\bar{g}|1\rangle \;\sqrt{N}=P_{jk}^{\alpha }g^{-1}|1\rangle \;\sqrt{N}\\ =\sum_{j^{\prime }=1}^{\ell^{\alpha }}\sum_{k^{\prime }=1}^{\ell^{\alpha }}D_{j^{\prime }k^{\prime }}^{\mu }(g^{-1})\;P_{jk}^{\alpha }P_{j^{\prime }k^{\prime }}^{\alpha }|1\rangle \;\sqrt{N}=\sum_{j^{\prime }=1}^{\ell^{\alpha }}D_{kk^{\prime }}^{\alpha }(g^{-1})\;P_{jk^{\prime }}^{\alpha }|1\rangle \;\sqrt{N}\\ =\sum_{j^{\prime }=1}^{\ell^{\alpha }}D_{kk^{\prime }}^{\alpha }(g^{-1}){|}_{jk^{\prime }}^{\alpha }\rangle =\sum_{j^{\prime }=1}^{\ell^{\alpha }}D_{k^{\prime }k}^{\alpha *}(g){|}_{jk^{\prime }}^{\alpha }\rangle \end{array}$$

A summary of the results is consistent with the block matrix forms in Equations (87) and (88).

(100) $$\langle_{j^{\prime }k}^{\alpha }|g{|}_{jk}^{\alpha }\rangle =D_{j^{\prime }j}^{\alpha }(g),\;\;\;\langle_{j^{\prime }k}^{\alpha }|g{|}_{jk}^{\alpha }\rangle =D_{k^{\prime }k}^{\alpha *}(g)$$

Choice of subgroup *C*_2_ = [**1***,***i_3_**] in Equation (78) leads to (*x, y*)-polarized states (*m*)_2_ labeled by their **i**_3_ eigenvalues (−1)*^m^*.

(101) $$\begin{array}{r} \langle_{j^{\prime }k}^{\alpha }|i_{3}{|}_{jk}^{\alpha }\rangle =D_{j^{\prime }j}^{\alpha }(i_{3}),\;\;\;\langle_{j^{\prime }k}^{\alpha }|{\bar{i}}_{3}{|}_{jk}^{\alpha }\rangle =D_{k^{\prime }k}^{\alpha *}(i_{3}).\\ =\delta_{j^{\prime }j}\{\begin{array}{c} +1\text{for}:j=x\\ -1\text{for}:j=y \end{array},\;\;\;=\delta_{k^{\prime }k}\{\begin{array}{c} +1\text{for}:k=x\\ -1\text{for}:k=y \end{array} \end{array}$$

**Figure 19:**
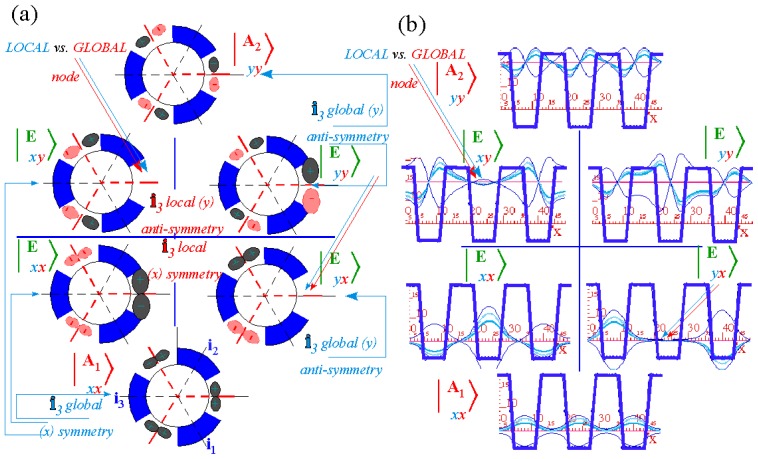
*D*_3_-symmetry waves (**a**) Sketch of projection; (**b**) 3-Well wave simulation (Compare with Figure 20).

Physical significance of these global-(*j*) and local-(*k*) values are now discussed using Figure 19.

Wherever the global *j* is *x* or **i**_3_-symmetric (0_2_), then the entire wave is symmetric to *x*-axial rotation by *π* in Figure 19a or horizontal reflection through the middle square-well in Figure 19b. Similarly, wherever the global *j* is *y* or **i**_3_-*anti*symmetric (1_2_), that is seen for each overall figure, too.

However, if the local *k* is *x* or **i**_3_-symmetric (0_2_), the local wave in *each* well has no node and is symmetric to its local axis of rotation by *π* in Figure 19a or horizontal reflection of *each* square-well in Figure 19b. Similarly, wherever the local *k* is *y* or **i**_3_-*anti*symmetric (1_2_), that antisymmetry and one node is seen in *each* well, too.

Local and global symmetry clash along the **i**_3_-axis for states projected by nilpotent **P***_xy_^α^* or **P***_yx_^α^*. The result is the *x*-axial wave nodes indicated by pairs of arrows in Figure 19. The |*E_yx_*〉 wave in the lower right of Figure 19b appears quite suppressed on the **i**_3_-axis. However, the simulation of the |*E_xy_*〉 in the upper left seems to have its “node” coming unglued.

The “unglued” level *ω_xy_^E^* is higher than *ω_yx_^E^* and enjoys more tunneling. If tunneling increases so do parameters such as *r*_1_ and *r*_2_ in Equation (92) that do not respect *x*-axial local subgroup *C*_2_ = [**1***,***i_3_**]. This breaks *x*-axial nodes and **i**_3_ local symmetry causing *E*-modes to be less *C*_2_-local and more like current-carrying above-barrier *C*_3_-local waves rotating on *r*-paths. *D*_3_ correlation arrays in Equation (102) with *C*_2_ or *C*_3_ indicate level cluster structure for extremes of each case.

(102) $$\begin{array}{ccc} D_{3}⊃C_{2} & 0_{2} & 1_{2}\\ A_{1} & 1 & \cdot \\ A_{2} & \cdot & 1\\ E & 1 & 1 \end{array}\;\;\;\begin{array}{cccc} D_{3}⊃C_{3} & 0_{3} & 1_{3} & 2_{3}\\ A_{1} & 1 & \cdot & \cdot \\ A_{2} & 1 & \cdot & \cdot \\ E & \cdot & 1 & 1 \end{array}$$

Column 0_2_ of array *D*_3_ ⊃ *C*_2_ in Equation (102) correlates to *A*_1_ and *E*. The lower (*A*_1_*, E*)-level cluster in Figure 19 has 0_2_ local symmetry and lies below cluster-(*A*_2_*, E*) that has local 1_2_ symmetry according to the 1_2_ column of Equation (102). Column 0_3_ of table *D*_3_ ⊃ *C*_3_ indicates that *A*_1_ and *A*_2_ levels cluster under extreme *C*_3_ localization, but columns 1_3_ and 2_3_ indicate that each *E* doublet level is unclustered under *C*_3_ with no extra degeneracy beyond its own (*ℓ^E^* = 2).

**Figure 20:**
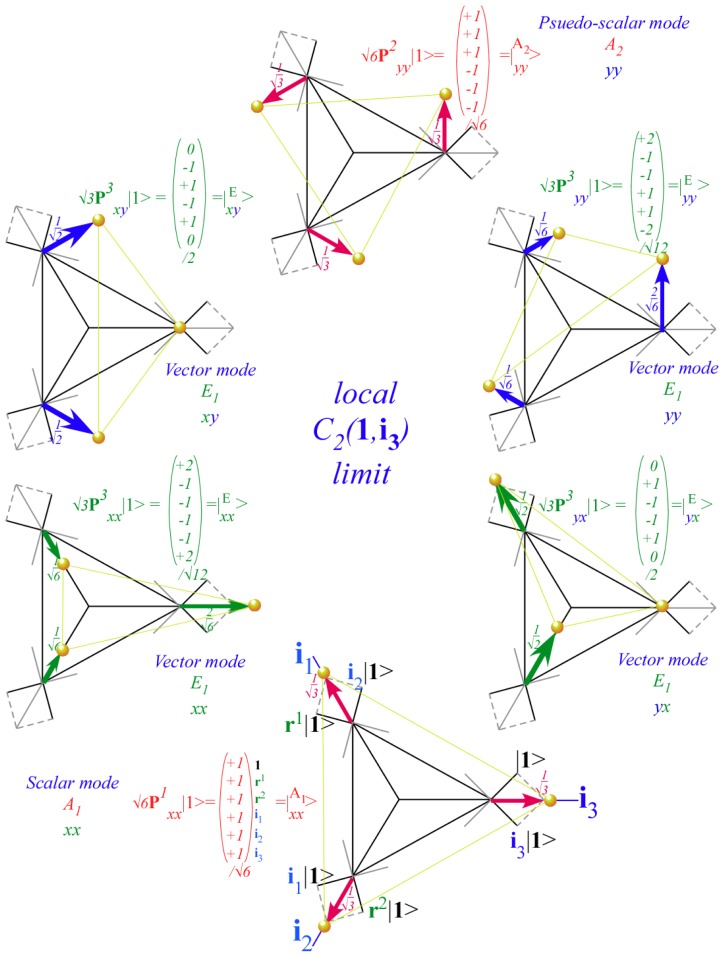
*D*_3_⊃*C*_2_(*i*_3_)-local symmetry modes of *X*_3_ molecule (Compare with Figure 19).

A classical analog of quantum waves states in Figure 19 is displayed in Figure 20 in the form of vibrational modes for an *X*_3_ molecule. A detailed description of this analogy in Appendix A includes modes of various local symmetry combinations analogous to those introduced above and in Sections 6.7.1 and 6.7.2 below.

### 6.7. Symmetry Correlation and Frobenius Reciprocity

The mathematical basis of correlation arrays in Equation (102) is a Frobenius reciprocity relation that exists between irreps of a group and its subgroups. This may be clarified by appealing to the physics of **P***_jk_^α^* -projected states |*_jk_^α^* 〉 such as are displayed in Figure 19 and by exploiting the duality between their local and global symmetry and subgroups.

*D*_3_-symmetric Hamiltonian **H** in (71) is made only of local **ḡ** that couple |*_jk_^α^* 〉-states through local *k*-indices by Equation (100) but leave all *ℓ^α^* values of global *j*-indices unchanged. Thus *α*-eigenstates of **H** mix *k*-values to form *ℓ^α^*-fold degenerate levels labeled by *j*-indices. (Recall *ℓ^E^* = 2 equal sub-matrices Equation (93) in (91).) Further degeneracy or near-degeneracy (“clustering”) occurs if inter-and-intra local tunneling coefficients decrease exponentially with quantum numbers thus isolating equivalent local modes into nearly degenerate sets of “spontaneously” broken local symmetry.

In contrast to this clustering or “un-splitting” associated with local **ḡ** symmetry operators, global **g** are associated with external or “applied” symmetry reduction that causes level *splitting*. Adding global **g***_m_* to a Hamiltonian **H** reduces its *G*-symmetry to a self-symmetry subgroup *K*=*s_m_* consisting of operators that commute with **g***_m_*. Adding a combination of **g***_m_* and **g***_n_* reduces *K* to an even smaller self-symmetry intersection group *s_m_* ∩ *s_n_*.

Global **g** couple |*_jk_^α^* 〉-states through global *j*-indices according to Equation (98). The more global perturbations are added to a Hamiltonian **H** the more likely it is to split *ℓ^α^*-fold *j*-degeneracy (for *ℓ^α^* ≥ 2) and/or linebreak alter eigenfunctions.

#### 6.7.1. Global “Applied” Symmetry Reduction, Subduction, and Level Splitting

In the *G*=*D*_3_ example, adding matrix (**r**^1^) from Equation (69a) to (**H**) in Equation (71) reduces its symmetry to *K*=*C*_3_=[**1***,***r**^1^*,***r**^2^], and adding (**i**_3_) reduces it to *K*=*C*_2_=[**1***,***i**_3_]. Adding a combination of (**r**^1^) and (**i**_3_) completely reduces (**H**)-symmetry to intersection *C*_3_∩*C*_2_=*C*_1_=[**1**], which corresponds to having *no* global symmetry.

By reducing *G* to a subgroup *K*⊂*G*, each *G*-labeled *α*-level becomes relabeled by that subgroup *K* and split (if *ℓ^α^* ≥ 2) in precisely the way that central *G*-idempotent **P***^α^* is relabeled and/or split by unit resolution shown in Equation (78) or (79). The splitting in Equation (79) of *D*_3_ idempotent **P***^E^* into *C*_3_-labeled **P**_1_3_1_3__*^E^* plus **P**_2_3_2_3__*^E^* implies the *D*_3_ doublet level *ω^E^* splits into *C*_3_-labeled singlets *ω*^1_3_^ and *ω*^2_3_^. Both *D*_3_ singlets *A*_1_ and *A*_2_ end up relabeled with *C*_3_ scalar 0_3_ labels.

(103) $$\begin{array}{llll} D_{3}⊃C_{3} & P^{\alpha }relabel/split & D^{\alpha }relabel/reduce & \omega^{\alpha }relabel/split\\ A_{1} & P^{A_{1}}=P^{A_{1}}P^{0_{3}}=P_{0_{3}0_{3}}^{A_{1}} & \Rightarrow D^{A_{1}}↓C_{3}\sim D^{0_{3}} & \Rightarrow \omega^{A_{1}}\to \omega^{0_{3}}\\ A_{2} & P^{A_{2}}=P^{A_{2}}P^{0_{3}}=P_{0_{3}0_{3}}^{A_{2}} & \Rightarrow D^{A_{2}}↓C_{3}\sim D^{0_{3}} & \Rightarrow \omega^{A_{2}}\to \omega^{0_{3}}\\ E & P^{E}=P^{E}P^{1_{3}}+P^{E}P^{2_{3}} & \Rightarrow D^{E}↓C_{3}\sim & \Rightarrow \omega^{E}\to \omega^{1_{3}}\\ & =P_{1_{3}1_{3}}^{E}+P_{2_{3}2_{3}}^{E} & D^{1_{3}}⊕D^{2_{3}} & ↘\omega^{2_{3}} \end{array}$$

Global *D*_3_⊃*C*_2_ relabeling and/or splitting is by Equation (78). Now *D*_3_ singlets have different labels 0_2_ and 1_2_.

(104) $$\begin{array}{llll} D_{3}⊃C_{2} & P^{\alpha }relabel/split & D^{\alpha }relabel/reduce & \omega^{\alpha }relabel/split\\ A_{1} & P^{A_{1}}=P^{A_{1}}P^{0_{2}}=P_{0_{2}0_{2}}^{A_{1}} & \Rightarrow D^{A_{1}}↓C_{2}\sim D^{0_{2}} & \Rightarrow \omega^{A_{1}}\to \omega^{0_{2}}\\ A_{2} & P^{A_{2}}=P^{A_{2}}P^{1_{2}}=P_{1_{2}1_{2}}^{A_{2}} & \Rightarrow D^{A_{2}}↓C_{2}\sim D^{1_{2}} & \Rightarrow \omega^{A_{2}}\to \omega^{1_{2}}\\ E & P^{E}=P^{E}P^{0_{2}}+P^{E}P^{1_{2}} & \Rightarrow D^{E}↓C_{2}\sim & \Rightarrow \omega^{E}\to \omega^{0_{2}}\\ & =P_{0_{2}0_{2}}^{E}+P_{1_{2}1_{2}}^{E} & D^{0_{2}}⊕D^{1_{2}} & ↘\omega^{1_{2}} \end{array}$$

Center portions of splitting relations in Equations (103) and (104) use *subduction* symbols (↓) to denote how each *D*_3_ irrep-*D^α^* reduces to subgroup *C*_3_ or *C*_2_ irreps under their respective global symmetry breaking. Earlier studies [34] have referred to these multiple subgroup splittings as multiple frameworks. Each *α*-row of Equations (103) and (104) corresponds to the row *α*=*A*_1_, *A*_2_, or *E*, of correlation array *D*_3_⊃*C*_3_ or *D*_3_⊃*C*_2_, respectively, in Equation (102).

#### 6.7.2. Local “Spontaneous” Symmetry Reduction, Induction, and Level Clustering

Opposite to global *G*⊃*K* symmetry irrep subduction *D^α^*(*G*)↓*K*=...⊕*d^a^*(*K*)⊕*d^b^*(*K*)⊕... that predicts level-splitting is the reverse relation of local *K*⊂*G* symmetry irrep *induction d^a^*(*K*)↑*G*=...⊕*D^α^*(*G*)⊕*D^β^*(*G*)⊕... that predicts “unsplitting” or level-clustering. In the former, an *ℓ^α^*-dimensional irrep *D^α^*(**k**) of global *G*-symmetry is reducible to smaller (*ℓ^a^* ≤ *ℓ^α^*) block-diagonal irreps *d^a^*(**k**) of a subgroup *K*. In the latter, a *K* irrep *d^a^* is induced (actually *projected*) kaleidoscope-like onto coset bases of a larger induced representation *d^a^*↑*G* of *G* that is generally reducible to *G* irreps *D^α^*.

Base states |*k* ↑*_j_^α^* 〉 of induced representation *d^k^*↑*G* are each made by a *G*-projector **P***_jk_^α^* acting on local *d^k^*-symmetry base state |*k*〉=**P***^k^*|*k*〉 defined by local *K*-projector **P***^k^*. *G*-projection is simpler if **P***_jk_^α^* is also based on *K*-projection. (It helps to stick with one framework through this!)

Of all *D*_3_⊃*C*_2_-projectors **P***_j_*__2__*_k_*__2__*^α^* based on Equation (78), only **P**_0_2_0_2__*^A^*^_1_^, **P**_0_2_0_2__*^E^*, and **P**_1_2_0_2__*^E^* have right index *k*_2_ = 0_2_. Only these can project induced states |0_2_ ↑*_j_*__2__*^α^* 〉 from local base state |0_2_〉 corresponding to the 0_2_-column of *D*_3_ ⊃ *C*_2_ array in Equation (102) having *A*_1_ and *E*. Similarly, *A*_2_ and *E* in the 1_2_-column of Equation (102) correspond to **P**_1_2_1_2__*^A^*^_2_^, **P**_0_2_1_2__*^E^*, and **P**_1_2_1_2__*^E^* projecting states |1_2_ ↑*_j_*__2__*^α^* 〉 from a local |1_2_〉 state. Each projector **P***_j_*__2__*_k_*__2__*^α^* in Equation (104) has a *C*_2_-subgroup projector **P***^k^*^_2_^ “right-guarding” the side facing each local *ℓ*_2_-ket |*ℓ*_2_〉 = **P***^ℓ^*^_2_^|*ℓ*_2_〉 that is similarly “guarded” by its own defining projector **P***^ℓ^*^_2_^. *C*_2_-subgroup projector orthogonality then allows only *k*_2_=*ℓ*_2_, giving the projection selection rules just described.

(105) $$P_{j_{2}k_{2}}^{\alpha }|\ell_{2}\rangle =P_{j_{2}k_{2}}^{\alpha }P^{k_{2}}P^{\ell_{2}}|\ell_{2}\rangle =\delta^{k_{2}\ell_{2}}P_{j_{2}\ell_{2}}^{\alpha }|\ell_{2}\rangle =\delta^{k_{2}\ell_{2}}|\ell_{2}{↑}_{j_{2}}^{\alpha }\rangle$$

Each “right guard” projector **P***^k^* of **P***_jk_^α^* is part of a *G*⊃*K* subgroup splitting or subduction splitting *D^α^*(*G*)↓*K*=...⊕*d_k_*(*K*)⊕... as shown by *D*_3_↓*C*_2_ examples in Equation (104). (These go back to the original *D*_3_⊃*C*_2_ subgroup chain resolution in Equation (78).) In Equation (105) each **P***^k^* selects which *α*-type induced bases |*k* ↑*_j_^α^* 〉 and block-diagonal *α*-irreps can appear in a *k*-induced representation *d^k^*(*K*)↑*G*=...⊕*D^α^*(*G*)⊕..., and it implies a duality between induced (↑) level-clustering and subduced (↓) level-splitting as stated by the following *Frobenius reciprocity relation*.

(106) $$\text{Number of }D^{\alpha }\;\text{in }d^{k}(K)↑G=\text{Number of }d^{k}\;\text{in }D^{\alpha }(G)↓K$$

The numbers on the left-hand side of Equation (106) would reside in the *k^th^*-column of a *G*⊃*K*-correlation array such as in Equation (102) while the numbers on the right-hand side of Equation (106) would reside in the *α^th^*-row of the *same* array. The examples in Equation (102) have only ones {1} and zeros {·}. A deeper correlation *D*_3_⊃*C*_1_ to *C*_1_ symmetry, *i.e*., to *no* symmetry is a conflation of either the array *D*_3_⊃*C*_2_ or the array *D*_3_⊃*C*_3_ in Equation (102) since *C*_1_=*C*_2_∩*C*_3_ is the intersection of *C*_2_ and *C*_3_.

(107) $$\begin{array}{cc} D_{3}⊃C_{1} & 0_{1}=1_{1}\\ A_{1} & 1\\ A_{2} & 1\\ E & 2 \end{array}$$

*C*_1_ local symmetry base |0_1_〉=|1_1_〉 is the |**1**〉 in Figure 18 that contains scalar *A*_1_, pseudo-scalar *A*_2_, and two *E* wave states in Figure 19 consistent with a single column of *D*_3_⊃*C*_1_ correlation array in Equation (107). This column describes *induced* representation *D*^0_1_^ (*C*_1_) ↑ *D*_3_, also known as a *regular* representation of *D*_3_.

Reciprocity in Equation (106) also holds for non-Abelian subgroup irreps *d^k^*. *D*_3_ is the smallest non-Abelian group so it has no such subgroups, but octahedral symmetry has non-Abelian *D*_3_ and *D*_4_ subgroups that figure in its splitting and clustering that are described in later Section 7.

#### 6.7.3. Coset Structure and Factored Eigensolutions

Three pairs of kets in Figure 17 relate to *left cosets* [**1***C*_2_ = (**1***,***i**_3_), **r***C*_2_ = (**r**^1^*,***i**_2_), **r**^2^*C*_2_ = (**r**^2^*,***i**_1_)] one at each site.

(108) $$\left[(|1\rangle ,|i_{3}\rangle ),\;\;\;(|r^{1}\rangle ,|i_{2}\rangle )=r^{1}(|1\rangle ,|i_{3}\rangle ),\;\;\;(|r^{2}\rangle ,|i_{1}\rangle )=r^{2}(|1\rangle ,|i_{3}\rangle )\right]$$

Conjugate bras 〈**g**|=〈**1**|**g**^†^relate to *right cosets* [*C*_2_=(**1***,***i**_3_), *C*_2_**r**^2^=(**r**^2^*,***i**_2_), *C*_2_**r**=(**r***,***i**_1_)], again, one per *C*_2_-well site.

(109) $$\left[(\langle 1|,\langle i_{3}|),\;\;\;(\langle r^{1}|,\langle i_{2}|)=(\langle 1|,\langle i_{3}|)r^{2},\;\;\;(\langle r^{2}|,\langle i_{1}|)=(\langle 1|,\langle i_{3}|)r^{1}\right]$$

*C*_2_ projectors **P**^0_2_^=_2_^1^ (**1**+**i**_3_)=**P***^x^* and **P**^1_2_^=_2_^1^ (**1**-**i**_3_)=**P***^y^* split bra 〈**g**| into ±-sum of bras mapped by left coset **g**^†^*C*_2_.

(110) $$\left[\langle 1|P^{m_{2}}{=}_{2}^{1}(\langle 1|\pm \langle i_{3}),\;\langle r^{1}|P^{m_{2}}{=}_{2}^{1}(\langle r^{1}|\pm \langle i_{2}|),\;\langle r^{2}|P^{m_{2}}{=}_{2}^{1}(\langle r^{2}|\pm \langle i_{1}|)\right]$$

The same projectors split ket |**g**〉 into bases **P***^m^*^_2_^ |**g**〉 that are ±-sum of kets mapped by right coset *C*_2_**g**.

(111) $$\left[P^{m_{2}}|1\rangle {=}_{2}^{1}(|1\rangle \pm |i_{3}\rangle ),\;P^{m_{2}}|r^{1}\rangle {=}_{2}^{1}(|r^{1}\rangle \pm |i_{2}\rangle ),\;P^{m_{2}}|r^{2}\rangle {=}_{2}^{1}(|r^{2}\rangle \pm |i_{1}\rangle )\right]$$

*g*-coefficients in *H*-submatrix Equation (93) track *C*_2_ cosets. Row-(bra)-*x* terms in *H_x,_*_·_*^E^* line up in (+)-right-coset **1g**+**i**_3_**g** order ...(*r*_1_+*i*_1_)*,* (*r*_2_+*i*_2_). Row-(bra)-*y* terms in *H_y,_*_·_*^E^* line up in (−)-right-coset **1g**-**i**_3_**g** order (*r*_1_-*i*_1_)*,* (*r*_2_-*i*_2_). Column-(ket) (±)-forms *H*_·_*_,x_^E^* and *H*_·_*_,y_^E^* line up in *left*-coset order ...(*r*_1_±*i*_2_)*,* (*r*_2_±*i*_1_). Either ordering gives the same matrix. Off-diagonal components *H_x,y_^E^* and *H_y,x_^E^* have *x vs. y* symmetry conflicts so coset parameters (*r*^0^ ± *i*_3_) vanish.

(112) $$\begin{array}{l} \left(\begin{array}{cc} H_{[x]x}^{E} & H_{[x]y}^{E}\\ H_{[y]x}^{E} & H_{[y]y}^{E} \end{array}\right)={\left(\begin{array}{cc} \left(r_{0}+i_{3})-\frac{1}{2}(r_{1}+i_{1})-\frac{1}{2}(r_{2}+i_{2}\right) & 0\cdot (r_{0}+i_{3})-\frac{\sqrt{3}}{2}(r_{1}+i_{1})+\frac{\sqrt{3}}{2}(r_{2}+i_{2})\\ 0\cdot (r_{0}-i_{3})+\frac{\sqrt{3}}{2}(r_{1}-i_{1})-\frac{\sqrt{3}}{2}(r_{2}-i_{2}) & \left(r_{0}-i_{3})-\frac{1}{2}(r_{1}-i_{1})-\frac{1}{2}(r_{2}-i_{2}\right) \end{array}\right)}_{bra}\\ \left(\begin{array}{cc} H_{x[x]}^{E} & H_{x[y]}^{E}\\ H_{y[x]}^{E} & H_{y[y]}^{E} \end{array}\right)={\left(\begin{array}{cc} \left(r_{0}+i_{3})-\frac{1}{2}(r_{1}+i_{2})-\frac{1}{2}(r_{2}+i_{1}\right) & 0\cdot (r_{0}+i_{3})-\frac{\sqrt{3}}{2}(r_{1}-i_{2})+\frac{\sqrt{3}}{2}(r_{2}-i_{1})\\ 0\cdot (r_{0}+i_{3})+\frac{\sqrt{3}}{2}(r_{1}+i_{2})-\frac{\sqrt{3}}{2}(r_{2}+i_{1}) & \left(r_{0}-i_{3})-\frac{1}{2}(r_{1}-i_{2})-\frac{1}{2}(r_{2}-i_{1}\right) \end{array}\right)}_{ket} \end{array}$$

**Figure 21:**
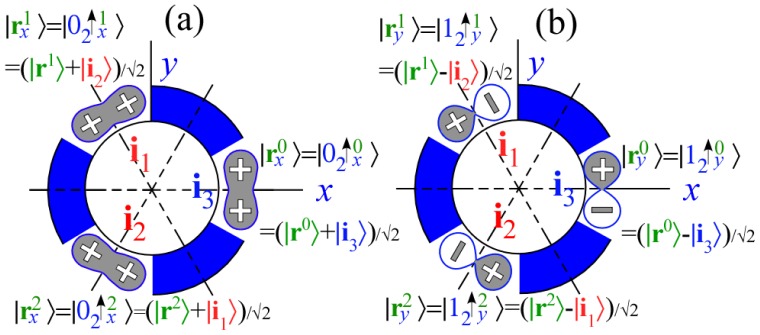
Induced representation *C*_2_↑*D*_3_ base wave states at vertex points *p* = 0*,* 1*,* and 2. (**a**) 0_2_↑*D*_3_ bases $P^{x}|r^{p}\rangle \sqrt{2}$ (**b**) 1_2_↑*D*_3_ bases $P^{y}|r^{p}\rangle \sqrt{2}$.

Kets **P***^x^*|**r***^p^*〉=[**P***^x^*|**1**〉, **P***^x^*|**r**^1^〉, **P***^x^*|**r**^2^〉 span induced representation *d^x^*(*C*_2_)↑*D*_3_, and **P***^y^*|**r***^p^*〉 span *d^y^*(*C*_2_)↑*D*_3_. Normalized states $P^{x}|r^{p}\rangle \sqrt{2}$ and $P^{y}|r^{p}\rangle \sqrt{2}$ correspond to *σ*-type and *π*-type orbitals at vertex positions *p*=0, 1*,* or 2 in Figure 21. *D*_3_ table in Equation (68) is reordered in Equation (113) below to display *C*_2_(**i**_3_) body-basis right-coset representation bra-defined by 〈**g**|=〈**1**| **ḡ** or ket-defined by **ḡ**^†^ |**1**〉=|**g**〉. The resulting *H*-matrix in Equation (68) is Equation (71) reordered for cosets of *C*_2_ instead of *C*_3_.

(113) $$\begin{array}{l} \begin{array}{ccccccc} \begin{array}{l} D_{3}\;body\\ gg^{†}form \end{array} & |1\rangle & |i_{3}\rangle ={\bar{i}}_{3}|1\rangle & |r^{1}\rangle ={\bar{r}}^{2}|1\rangle & |i_{2}\rangle ={\bar{i}}_{3}{\bar{r}}^{2}|1\rangle & |r^{2}\rangle ={\bar{r}}^{1}|1\rangle & |i_{1}\rangle ={\bar{i}}_{3}{\bar{r}}^{1}|1\rangle \\ \langle 1| & 1 & {\bar{i}}_{3} & {\bar{r}}^{2} & {\bar{i}}_{2} & {\bar{r}}^{1} & {\bar{i}}_{1}\\ \langle i_{3}|=\langle 1|{\bar{i}}_{3} & {\bar{i}}_{3} & 1 & {\bar{i}}_{2} & {\bar{r}}^{2} & {\bar{i}}_{1} & {\bar{r}}^{1}\\ \langle r^{1}|=\langle 1|{\bar{r}}^{1} & {\bar{r}}^{1} & {\bar{i}}_{2} & 1 & {\bar{i}}_{1} & {\bar{r}}^{2} & {\bar{i}}_{3}\\ \langle i_{2}|=\langle 1|{\bar{r}}^{1}{\bar{i}}_{3} & {\bar{i}}_{2} & {\bar{r}}^{1} & {\bar{i}}_{1} & 1 & {\bar{i}}_{3} & {\bar{r}}^{2}\\ \langle r^{2}|=\langle 1|{\bar{r}}^{2} & {\bar{r}}^{2} & {\bar{i}}_{1} & {\bar{r}}^{1} & {\bar{i}}_{3} & 1 & {\bar{i}}_{2}\\ \langle i_{1}|=\langle 1|{\bar{r}}^{2}{\bar{i}}_{3} & {\bar{i}}_{1} & {\bar{r}}^{2} & {\bar{i}}_{3} & {\bar{r}}^{1} & {\bar{i}}_{2} & 1 \end{array}\\ \Rightarrow \langle H\rangle =\begin{array}{ccccccc} & |1\rangle & |i_{3}\rangle & |r^{1}\rangle & |i_{2}\rangle & |r^{2}\rangle & |i_{1}\rangle \\ \langle 1| & r_{0} & i_{3} & r_{2} & i_{2} & r_{1} & i_{1}\\ \langle i_{3}| & i_{3} & r_{0} & i_{2} & r_{2} & i_{1} & r_{1}\\ \langle r^{1}| & r_{1} & i_{2} & r_{0} & i_{1} & r_{2} & i_{3}\\ \langle i_{2}| & i_{2} & r_{1} & i_{1} & r_{0} & i_{3} & r_{2}\\ \langle r^{2}| & r_{2} & i_{1} & r_{1} & i_{3} & r_{0} & i_{2}\\ \langle i_{1}| & i_{1} & r_{2} & i_{3} & r_{1} & i_{2} & r_{0} \end{array} \end{array}$$

*C*_2_ ordered products in Equation (113) help reduce *H*-matrix in Equation (71) to a direct sum of *C*_2_ induced reps (*d*^0_2_^⊕*d*^1_2_^)↑*D*_3_ in Equation (114). Upper (0_2_)-array in Equation (114) uses *σ*-orbital bases |**r***_x_^p^*〉 in Figure 21a while *π*-orbital bases |**r***_y_^p^* 〉 in Figure 21b span the (1_2_)-array.

(114) $$\langle H\rangle =\begin{array}{ccccccc} & |0_{2}{↑}_{x}^{0}\rangle & |0_{2}{↑}_{x}^{1}\rangle & |0_{2}{↑}_{x}^{2}\rangle & |1_{2}{↑}_{y}^{0}\rangle & |1_{2}{↑}_{y}^{1}\rangle & |1_{2}{↑}_{y}^{2}\rangle \\ \langle_{x}^{0}| & r_{0}+i_{3} & r_{2}+i_{2} & r_{1}+i_{1} & \cdot & \cdot & \cdot \\ \langle_{x}^{1}| & r_{1}+i_{2} & r_{0}+i_{1} & r_{2}+i_{3} & \cdot & \cdot & \cdot \\ \langle_{x}^{2}| & r_{2}+i_{1} & r_{1}+i_{3} & r_{0}+i_{2} & \cdot & \cdot & \cdot \\ \langle_{y}^{0}| & \cdot & \cdot & \cdot & r_{0}-i_{3} & r_{2}-i_{2} & r_{1}-i_{1}\\ \langle_{y}^{1}| & \cdot & \cdot & \cdot & r_{1}-i_{2} & r_{0}-i_{1} & r_{2}-i_{3}\\ \langle_{y}^{2}| & \cdot & \cdot & \cdot & r_{2}-i_{1} & r_{1}-i_{3} & r_{0}-i_{2} \end{array}$$

Any group component of Equation (114) or combination thereof is a possible tunneling matrix. Submatrices *d*^0_2_^(**g**)↑*D*_3_ shown for **g**=**r^1^**, **i_1_**, and **i_3_** reflect the effect of these operators on states in Figure 21a and similarly for *d*^1_2_^(**g**)↑*D*_3_ in Figure 21b.

(115) $$\begin{array}{l} \langle r_{1}{\bar{r}}^{1}\rangle =r_{1}\begin{array}{cccccc} \cdot & \cdot & 1 & \cdot & \cdot & \cdot \\ 1 & \cdot & \cdot & \cdot & \cdot & \cdot \\ \cdot & 1 & \cdot & \cdot & \cdot & \cdot \\ \cdot & \cdot & \cdot & \cdot & \cdot & 1\\ \cdot & \cdot & \cdot & 1 & \cdot & \cdot \\ \cdot & \cdot & \cdot & \cdot & 1 & \cdot \end{array},\;\;\;\langle i_{1}{\bar{i}}_{1}\rangle =i_{1}\begin{array}{cccccc} \cdot & \cdot & 1 & \cdot & \cdot & \cdot \\ \cdot & 1 & \cdot & \cdot & \cdot & \cdot \\ 1 & \cdot & \cdot & \cdot & \cdot & \cdot \\ \cdot & \cdot & \cdot & \cdot & \cdot & -1\\ \cdot & \cdot & \cdot & \cdot & -1 & \cdot \\ \cdot & \cdot & \cdot & -1 & \cdot & \cdot \end{array},\\ \langle i_{3}{\bar{i}}_{3}\rangle =i_{3}\begin{array}{cccccc} 1 & \cdot & \cdot & \cdot & \cdot & \cdot \\ \cdot & \cdot & 1 & \cdot & \cdot & \cdot \\ \cdot & 1 & \cdot & \cdot & \cdot & \cdot \\ \cdot & \cdot & \cdot & -1 & \cdot & \cdot \\ \cdot & \cdot & \cdot & \cdot & \cdot & -1\\ \cdot & \cdot & \cdot & \cdot & -1 & \cdot \end{array} \end{array}$$

The 0_2_ correlation in Equation (102) implies *d*^0_2_^↑*D*_3_ reduces further to *D*_3_ irreps *A*_1_⊕*E* that label the lower band of Figure 19. Meanwhile *d*^1_2_^↑*D*_3_ reduces to irreps *A*_2_⊕*E* that label the upper band of Figure 19. Equation (91) shows *A*_1_⊕*A*_2_⊕*E*⊕*E*.

## 7. Octahedral Symmetry Analysis

**Figure 22:**
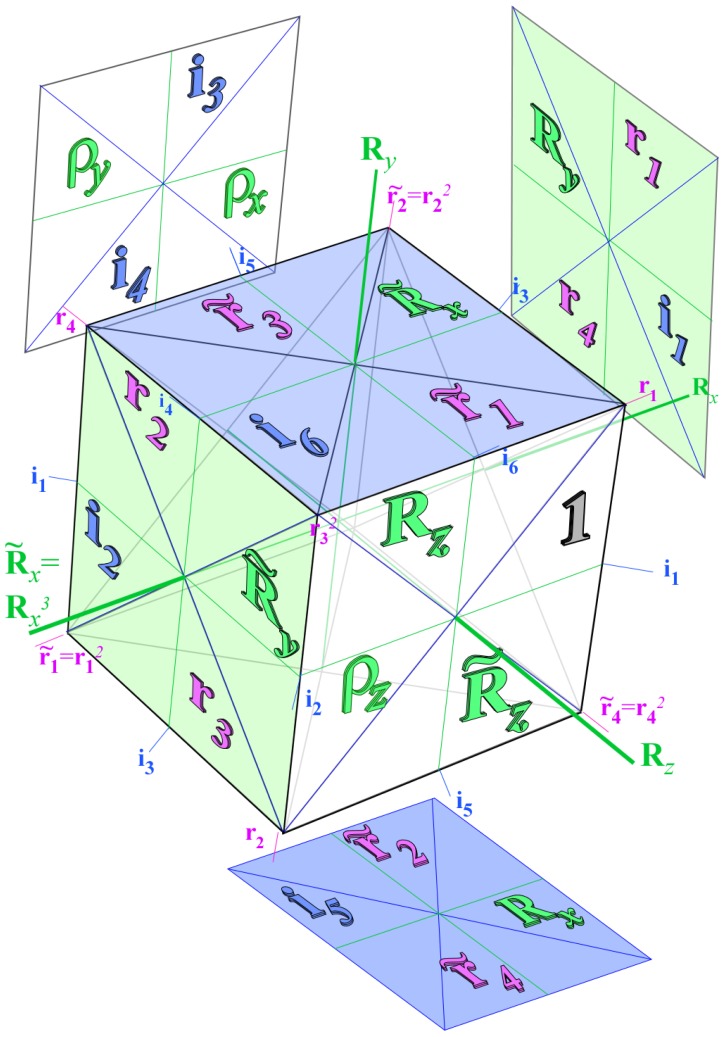
*O* operators distributed in cosets of *C*_4_ ⊃ *C*_2_.

**Figure 23:**
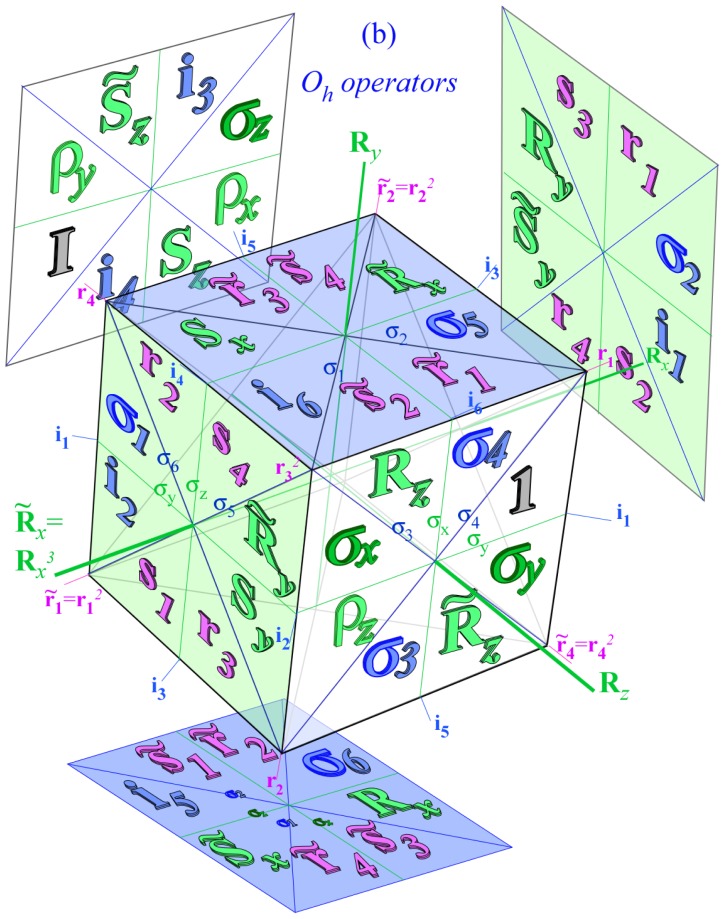
*O_h_* operators distributed in cosets of *C*_4_*_v_* ⊃ *C*_2_*_v_*.

Octahedral-cubic rotational symmetry *O* operations are modeled in Figure 22. Rotation inversion symmetry *O_h_*=*O*×*C_i_* operations are modeled in Figure 23. In each case the larger **g**-symbols (such as **r̃_1_** on top of Figure 22) label position ket states (such as |**r̃_1_**〉=**r̃_1_**|**1**〉) while smaller **g**-symbols label axes of rotation in *O* (such as *i*_6_ on top facing edge of Figure 22 labeling that 180° rotation) or planes of reflection in *O_h_* (such as the *σ_x_* just above the *z*-axis on facing plane of Figure 23 labeling the *x*-plane reflection).

Figure 22 is an “*O*-group slide-rule” since product **i_6_** · **r̃_1_** can be viewed as operator *i*_6_ flipping a wave in position |**r̃_1_**〉 onto position |**R_z_**〉, that is, *i*_6_|**r̃_1_**〉=|**R_z_**〉 giving product **i_6_** · **r̃_1_**=**R***_z_*. Figure 23 is an “*O_h_*-group slide-rule” (that does *O* products, too) and just as easily gives product *σ***_x_** · **r̃_1_**=**s̃_2_** all without knowing what **r̃_1_** or **s̃_2_** do. (As explained below, **r_1_** is 120° rotation about [111] axis and **r̃_1_** is its inverse located on the [1̄ 1̄ 1̄]-axis while **r̃_2_** is on the [111̄] axis. **s̃_2_** is **r̃_2_** multiplied by inversion **I**·[111]=[1̄ 1̄ 1̄].)

Note *i*_6_-transform of *state* |**r_1_**〉 (example: *i*_6_|**r_1_**〉=|**R̃_y_**〉) differs from an *i*_6_-transform of *operator***r_1_** (example: **i_6_**·**r_1_**·**i_6_**^−1^=**r_3_**^2^). The latter is divined easily by “slide-rule” as *i*_6_ flips **r_1_**’s axis onto **r_3_**^2^’s.

Three Cartesian *C*_4_ axes of anti-clockwise 90° rotations **R***_x_*, **R***_y_*, and **R***_z_* define directions [100], [010], and [001], respectively. Their inverses **R̃_x_**=**R***_x_*^3^, **R̃_y_**=**R***_y_*^3^*,* and **R̃_z_**=**R***_z_*^3^ are also 90° rotations but around negative axes [1̄00], [01̄0], and [001̄]. A shorthand notation for 180° Cartesian rotations is *ρ_x_*=**R***_x_*^2^, *ρ_y_*=**R***_y_*^2^*,* and *ρ_z_*=**R***_z_*^2^. Trigonal *C*_3_ axes of anti-clockwise 120° rotations **r**_1_, **r**_2_, **r**_3_, and **r**_4_ lie along [111], [1̄1̄1], [1̄11], and [1̄11̄], respectively, while axes of inverses **r̃**_1_=**r**_1_^2^, **r̃**_2_=**r**_2_^2^, **r̃**_3_=**r**_3_^2^, and **r̃**_4_=**r**_4_^2^ lie along the opposite directions [1̄ 1̄ 1̄], [111̄], [11̄1̄], and [11̄1], respectively.

There are six *C*_2_ axes of 180° rotations **i**_1_, **i**_2_, **i**_3_, **i**_4_, **i**_5_, and **i**_6_ located along [101], [1̄01], [110], [1̄10], [011], and [01̄1], respectively. This completes the five classes of *O*: [**1**], [**r**_1..4_*,***r̃**_1..4_], [*ρ_xyz_*], [**R***_xyz_,***R̃***_xyz_*], and [**i**_1..6_]. Including the rotations with inversion **I** yields five more classes of *O_h_*: [**I**], [**s**_1..4_*,***s̃**_1..4_], [*ρ_xyz_*], [**S***_xyz_,***S̃***_xyz_*], and [*σ*_1..6_] where **s**_1..4_=**I** · **r**_1..4_, [*σ_xyz_*]=[**I** · *ρ_xyz_*], [**S***_xyz_*]=[**I** · **R***_xyz_*], and [*σ*_1..6_]=[**I** · **i**_1..6_]. *σ*’s are mirror-plane reflections in Figure 23.

The “slide-rules” in Figures 22, 23 also help evaluate class products and construct left and right *cosets* of local symmetry subgroups. Three of the largest cyclic subgroups of *O* are tetragonal *C*_4_ such as *C*_4_=[**1***,***R***_z_,***R***_z_*^2^ =*ρ_z_,***R***_z_*^3^ =**R̃***_z_*] displayed on the **R***_z_*-face of the cube in Figure 22. In Figure 23 the same face displays local symmetry *C*_4_*_v_*=[**1***, ρ_z_,***R***_z_,***R̃***_z_, σ*_4_*, σ_x_, σ*_3_*, σ_y_*] that contains *C*_4_ plus pairs of diagonal mirror reflections [*σ*_4_=**I**·**i**_4_, *σ*_3_=**I**·**i**_3_] and Cartesian mirror reflections [*σ_x_*=**I**·*ρ_x_*, *σ_y_*=**I**·*ρ_y_*]. Each pair [*σ_x_, σ_y_*] and [*σ*_3_*, σ*_4_] is a *C*_4_*_v_* class as is rotation pair [**R***_z_,***R̃***_z_*] or, singly, **1** and *ρ_z_*. The other five cube faces display cosets of the tetragonal subgroups *C*_4_*_v_*⊃*C*_4_ of *O_h_*⊃*O*.

Figure 22 shows six *O*-cosets **g**·*C*_4_ of *C*_4_=[**1***,***R***_z_, ρ_z_,***R̃***_z_*]. Opposite *ρ_x_*-face has coset *ρ_x_*·*C*_4_=[*ρ_x_,***i**_4_*, ρ_y_,***i**_3_] in that order. The **r**_1_-face shows coset **r**_1_·*C*_4_=[**r**_1_*,***i**_1_*,***r**_4_*,***R***_y_*] in upper right of Figure 22, and the opposite **r**_2_-face has coset **r**_2_·*C*_4_=[**r**_2_*,***i**_2_*,***r**_3_*,***R̃***_y_*]. Top and bottom faces have cosets **r̃**_1_·*C*_4_=[**r̃**_1_*,***R̃***_x_,***r̃**_3_*,***i**_6_] and **r̃**_2_·*C*_4_=[**r̃**_2_*,***R***_x_,***r̃**_4_*,***i**_5_].

Each **g**·*C*_4_-coset element **g**·**R***_z_^p^* (*p* = 0..3) transforms the **1**-face to the same **g**-face and orients it according to a *C*_4_ element **R***_z_^p^* as it permutes the list of its elements accordingly. Each face may be labeled by any element **g**·**R***_z_^p^* in its coset. An **i**-class labeling by **1**, **i**_3_(or **i**_4_), **i**_1_, **i**_2_, **i**_6_, and **i**_5_ of *C*_4_ cosets in Figure 22 is as good as any other.

**Figure 24:**
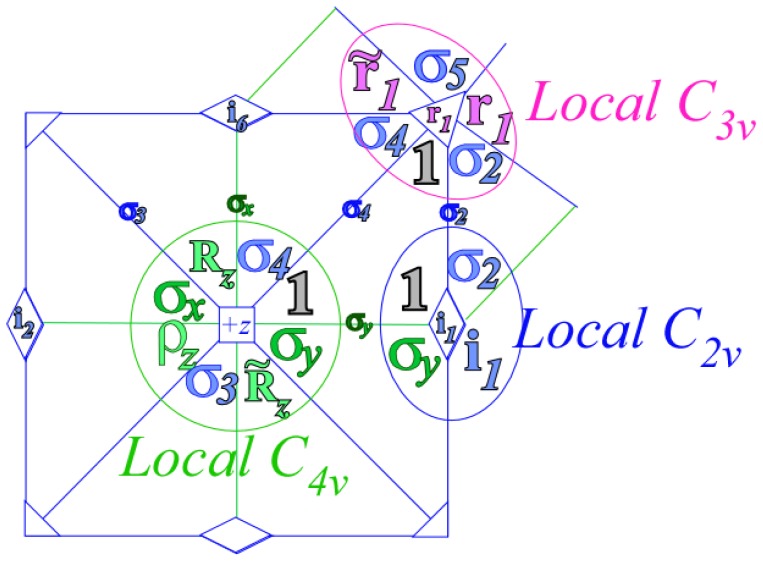
*O_h_* local symmetry (**a**) *C*_4_*_v_*; (**b**) *C*_3_*_v_*; (**c**) *C*_2_*_v_*.

Figure 23 shows six *O_h_*-cosets of *C*_4_*_v_* (counting *C*_4_*_v_* itself) in a geometric display that also shows eight trigonal cosets of *C*_3_*_v_*⊃*C*_3_-[111] and twelve dihedral cosets of *C*_2_*_v_*⊃*C*_2_-[101]. Figure 24 shows three symmetry points of Figure 23 forming a triangular cell with sides that are on reflection planes.

An order-8 axial symmetry *C*_4_*_v_* lies on the tetragonal-*z*-[001]-axis of a cube face or octahedral vertex. An order-6 *C*_3_*_v_* lies on the trigonal-[111]-axis of a cube vertex or octahedral face. Finally, there is a dihedral-*C*_2_*_v_* [110]-axis of a cube or octahedral edge. Lines between the axes have bilateral local reflection symmetry *C_v_*(*y*)=[**1***, σ_y_*], *C_v_*(2)=[**1***, σ*_2_], or *C_v_*(4)=[**1***, σ*_4_], fundamental symmetry operations whose products generate all others. Figure 24 is like a reduced Brillouin Zone of the *O_h_* lattice.

Each subgroup spawns a coset space and a set of induced representations of full *O_h_* symmetry that generalize the *C*_3_*_v_* induced representations in Equation (115) and base kets sketched in Figure 21. Correlation tables between *O* or *O_h_* and its subgroups *L*⊂*G* tell which *O* or *O_h_* irreps, states, and energy levels arise from each coset space. As local symmetry reduces and its order °*L* decreases, the coset dimension *d*=°*G*/°*L* grows proportionally with a corresponding increase in number of irreps and levels in *L*↑*G*-induced representation cluster spaces. Examples are given below for *G*=*O* and in Section 8 for *G*=*O_h_*.

### 7.1. Octahedral Characters and Subgroup Correlations

Spectral class resolution of *O* generalizes that of *D*_3_ in Equation (75) to give character array Equation (116).

(116) $$\begin{array}{cccccc} \begin{array}{c} O\;group\\ \chi_{\kappa_{g}}^{\alpha } \end{array} & g=1 & \begin{array}{l} r_{1-4}\\ {\tilde{r}}_{1-4} \end{array} & \rho_{xyz} & \begin{array}{l} R_{xyz}\\ {\tilde{R}}_{xyz} \end{array} & i_{1-6}\\ \alpha =A_{1} & 1 & 1 & 1 & 1 & 1\\ A_{2} & 1 & 1 & 1 & -1 & -1\\ E & 2 & -1 & 2 & 0 & 0\\ T_{1} & 3 & 0 & -1 & 1 & -1\\ T_{2} & 3 & 0 & -1 & -1 & 1 \end{array}$$

Cyclic subgroup *C*_4_(**R***_z_^p^)*, *C*_3_(**r**_1_*^p^*), and *C*_2_ characters correlate to *O* according to arrays in Equation (117).

(117) $$\begin{array}{c} \begin{array}{ccccc} O⊃C_{4} & 0_{4} & 1_{4} & 2_{4} & 3_{4}\\ A_{1}↓C_{4} & 1 & \cdot & \cdot & \cdot \\ A_{2}↓C_{4} & \cdot & \cdot & 1 & \cdot \\ E↓C_{4} & 1 & \cdot & 1 & \cdot \\ T_{1}↓C_{4} & 1 & 1 & \cdot & 1\\ T_{2}↓C_{4} & \cdot & 1 & 1 & 1 \end{array}\;\;\;\begin{array}{cccc} O⊃C_{3} & 0_{3} & 1_{3} & 2_{3}\\ A_{1}↓C_{3} & 1 & \cdot & \cdot \\ A_{2}↓C_{3} & 1 & \cdot & \cdot \\ E↓C_{3} & \cdot & 1 & 1\\ T_{1}↓C_{3} & 1 & 1 & 1\\ T_{2}↓C_{3} & 1 & 1 & 1 \end{array}\\ \begin{array}{ccc} O⊃C_{2}(i_{1}) & 0_{2} & 1_{2}\\ A_{1}↓C_{2} & 1 & \cdot \\ A_{2}↓C_{2} & \cdot & 1\\ E↓C_{2} & 1 & 1\\ T_{1}↓C_{2} & 1 & 2\\ T_{2}↓C_{2} & 2 & 1 \end{array}\;\;\;\begin{array}{ccc} O⊃C_{2}(\rho_{z}) & 0_{2} & 1_{2}\\ A_{1}↓C_{2} & 1 & \cdot \\ A_{2}↓C_{2} & 1 & \cdot \\ E↓C_{2} & 2 & \cdot \\ T_{1}↓C_{2} & 1 & 2\\ T_{2}↓C_{2} & 1 & 2 \end{array} \end{array}$$

Equivalent subgroup correlations *O*⊃*H* and *O*⊃**g***H***g**^−1^ share elements in the same *O*-classes and have one correlation array. Thus all three *C*_4_ local symmetries have one correlation table in Equation (117), as do all four *C*_3_ subgroups. However, *O*⊃*C*_2_(*ρ_z_*) and *O*⊃*C*_2_(**i**_1_) correlations differ since **i**_1_ and *ρ_z_* have different *O*-class and characters in Equation (116).

Projectors **P***_jk_^α^* and irreps *D_jk_^α^* of *O* depend on choice of local symmetry just as *D*_3_ projector splitting in Equation (78) or (79) depends on choice of correlation *D*_3_⊃*C*_2_ in Equation (104) or *D*_3_⊃*C*_3_ in Equation (103), respectively. Sub-labels (*j, k*) range over *C*_2_ values [0_2_*,* 1_2_] or else *C*_3_ values [0_3_*,* 1_3_*,* 2_3_] while a tetragonal correlation *O*⊃*C*_4_ will use sub-labels (*j, k*)= [0_4_*,* 1_4_*,* 2_4_*,* 3_4_].

The *m*_4_ or else *m*_3_ unambiguously defines all *O* states since no *O*⊃*C*_4_ or *O*⊃*C*_3_ correlation numbers in Equation (117) exceed unity. However, *O*⊃*C*_2_(**i**_1_) correlations cannot distinguish all three sub-levels of *T*_1_ or *T*_2_ wherever a number 2 appears, and the *O*⊃*C*_2_(*ρ_z_*) correlation leaves the *E* sub-levels unresolved, as well. A full *O_h_* labeling resolves the first ambiguity as shown below, but we consider the unambiguous *O*⊃*C*_4_ case first. (*C*_4_ resolves *C*_2_(*ρ_z_*) ambiguities.)

#### 7.1.1. Resolving Commuting *O*⊃*C*_4_ Local Symmetry Subalgebra: Rank = *ρ*(*O*) = 10

The *C*_4_ correlation table in Equation (117) shows how invariant class projectors **P***^α^* (expanded below in terms of *O* characters *χ_κ_*_*_g_*_^α^ in table shown in Equation (116)) will split into irrep projectors **P***_m_*__4__*_m_*__4__*^α^* when hit by *C*_4_ local symmetry projectors **p***_m_*__4__. The latter **p***_m_* are expanded in terms of *C*_4_ operators **R***_z_^p^* weighted by character eigenvalues *φ_p_^m^*^_4_^ = (*χ_p_^m^*^_4_^)^*^ using Equations (57) and (59).

(118) $$\begin{array}{ccccc} 1\cdot P^{\alpha }= & (p_{0_{4}} & +p_{1_{4}} & +p_{2_{4}} & +p_{3_{4}})\cdot P^{\alpha }\\ 1\cdot P^{A_{1}}= & P_{0_{4}0_{4}}^{A_{1}} & +0 & +0 & +0\\ 1\cdot P^{A_{2}}= & 0 & +0 & +P_{2_{4}2_{4}}^{A_{2}} & +0\\ 1\cdot P^{E}= & P_{0_{4}0_{4}}^{E} & +0 & +P_{2_{4}2_{4}}^{E} & +0\\ 1\cdot P^{T_{1}}= & P_{0_{4}0_{4}}^{T_{1}} & +P_{1_{4}1_{4}}^{T_{1}} & +0 & +P_{3_{4}3_{4}}^{T_{1}}\\ 1\cdot P^{T_{2}}= & 0 & +P_{1_{4}1_{4}}^{T_{2}} & +P_{2_{4}2_{4}}^{T_{2}} & +P_{3_{4}3_{4}}^{T_{2}} \end{array}$$

The five class projectors **P***^α^* are *O*-invariant and commute with all twenty-four *O*-operators (**1***,***r**_1_*,***r**_2_*, ...***i**_5_*,***i**_6_). So do the five class operators (*κ*_0_*, κ_r_*_*_k_*_, κ*_ρ_*_*_k_*_, κ*_R_*_*_k_*_, κ*_i_*_*_k_*_) in which each **P***^α^* is expanded as follows. (Recall *D*_3_ classes in Equation (75).)

(119) $$\begin{array}{l} P^{\alpha }=\frac{\ell^{\alpha }}{{}^{\circ}O}\sum_{k=0}^{5}\chi_{k}^{\alpha }\kappa_{k}=\;\;\;\text{where}:\alpha =A_{1},A_{2},E,T_{1},\;\text{or }T_{2}\\ =\frac{\ell^{\alpha }}{24}\left[\chi_{0}^{\alpha }1+\chi_{\kappa_{r}}^{\alpha }(r_{1}+r_{2}+\ldots +{\tilde{r}}_{4})+\chi_{\kappa_{\rho }}^{\alpha }(\rho_{x}+\rho_{y}+\rho_{z})+\chi_{\kappa_{R}}^{\alpha }(R_{x}+R_{y}+\ldots +{\tilde{R}}_{z})+\chi_{\kappa_{i}}^{\alpha }(i_{1}+i_{2}+\ldots +i_{6})\right] \end{array}$$

Each of the *ℓ^α^* irrep projectors **P***_n_*__4__*_n_*__4__*^α^* is obtained from its invariant **P***^α^* by product **P***^α^***p***_n_*__4__=**p***_n_*__4__**P***^α^* following Equation (118) with each of four *C*_4_ local symmetry projector **p***_m_*__4__.

(120) $$p_{m_{4}}=\sum_{p=0}^{3}\frac{e^{2\pi im\cdot p/4}}{4}R_{z}^{p}=\{\begin{array}{c} p_{0_{4}}=(1+R_{z}+\rho_{z}+{\tilde{R}}_{z})/4\\ p_{1_{4}}=(1+iR_{z}-\rho_{z}-i{\tilde{R}}_{z})/4\\ p_{2_{4}}=(1-R_{z}+\rho_{z}-{\tilde{R}}_{z})/4\\ p_{3_{4}}=(1-iR_{z}-\rho_{z}+i{\tilde{R}}_{z})/4 \end{array}$$

As the five (*O*-*centrum*=5) projectors **P***^α^* split into ten (*O*-*rank*=10) sub-projectors **P***_n_*__4__*_n_*__4__*^α^*, the five *O* class sums *κ_g_* split into ten *C*_4_-*invariant sub*-*class sums***c***_k_*(*k*=1..10).

(121) $$\begin{array}{r} \frac{{}^{\circ}O}{\ell^{\alpha }}\cdot P_{n_{4}n_{4}}^{\alpha }=\sum_{k=0}^{10}D_{n_{4}n_{4}}^{\alpha^{*}}(g_{k})c_{k}\\ \text{where}:\;\;\;D_{n_{4}n_{4}}^{\alpha }(g_{k})=D_{n_{4}n_{4}}^{\alpha }(R_{z}^{p†}g_{k}R_{z}^{p}) \end{array}$$

The resulting ten products $\frac{{}^{\circ}O}{\ell^{\alpha }}P_{n_{4}n_{4}}^{\alpha }$ are listed in Equation (122) of diagonal irrep coefficients *D_n_*__4__*_n_*__4__*^α^* (*g_k_*) in terms of twenty-four group elements *g_k_* that have been sorted into ten *sub*-*classes* that have *C*_4_(*z*) local symmetry. The ten irrep projectors **P***_n_*__4__*_n_*__4__*^α^* are *C*_4_ local-invariant, that is, they commute with four *C*_4_-operators (**1***,***R***_z_,***R***_z_*^2^ = *ρ_z_,***R***_z_*^3^ = *R̃_z_*) but not the whole *O* group like the **P***^α^* do. The ten sub-class-sum operators **c***_k_*, into which each **P***_n_*__4__*_n_*__4__*^α^* is expanded in Equation (122), are each individually invariant to **R***_z_^p^,* that is **R***_z_^p^***c***_k_*=**c***_k_***R***_z_^p^,* and *D_n_*__4__*_n_*__4__*^α^* (*g_k_*) is the same for all *g_k_* in sub-class *c_k_*. Note that a sum of *ℓ^α^* rows belonging to **P***_n_*__4__*_n_*__4__*^α^* between horizontal lines in Equation (122) yields corresponding character values *χ_k_^α^* =*trace D^α^*(*g_k_*) in *O*-character array Equation (116) and effectively “unsplits” the sub-classes.

(122) $$\begin{array}{ccccccccccc} P_{n_{4}n_{4}}^{\left(\alpha \right)}(O⊃C_{4}) & 1 & r_{1}r_{2}{\tilde{r}}_{3}{\tilde{r}}_{4} & {\tilde{r}}_{1}{\tilde{r}}_{2}r_{3}r_{4} & \rho_{x}\rho_{y} & \rho_{z} & R_{x}{\tilde{R}}_{x}R_{y}{\tilde{R}}_{y} & R_{z} & {\tilde{R}}_{z} & i_{1}i_{2}i_{5}i_{6} & i_{3}i_{4}\\ 24\cdot P_{0_{4}0_{4}}^{A_{1}} & 1 & 1 & 1 & 1 & 1 & 1 & 1 & 1 & 1 & 1\\ 24\cdot P_{2_{4}2_{4}}^{A_{2}} & 1 & 1 & 1 & 1 & 1 & -1 & -1 & -1 & -1 & -1\\ 12\cdot P_{0_{4}0_{4}}^{E} & 1 & -\frac{1}{2} & -\frac{1}{2} & 1 & 1 & -\frac{1}{2} & 1 & 1 & -\frac{1}{2} & 1\\ 12\cdot P_{2_{4}2_{4}}^{E} & 1 & -\frac{1}{2} & -\frac{1}{2} & 1 & 1 & +\frac{1}{2} & -1 & -1 & +\frac{1}{2} & -1\\ 8\cdot P_{1_{4}1_{4}}^{T_{1}} & 1 & -\frac{i}{2} & -\frac{i}{2} & 0 & -1 & +\frac{1}{2} & -i & +i & -\frac{1}{2} & 0\\ 8\cdot P_{3_{4}3_{4}}^{T_{1}} & 1 & +\frac{i}{2} & -\frac{i}{2} & 0 & -1 & +\frac{1}{2} & +i & -i & -\frac{1}{2} & 0\\ 8\cdot P_{0_{4}0_{4}}^{T_{1}} & 1 & 0 & 0 & -1 & 1 & 0 & 1 & 1 & 0 & -1\\ 8\cdot P_{1_{4}1_{4}}^{T_{2}} & 1 & +\frac{i}{2} & -\frac{i}{2} & 0 & -1 & -\frac{1}{2} & -i & +i & +\frac{1}{2} & 0\\ 8\cdot P_{3_{4}3_{4}}^{T_{2}} & 1 & -\frac{i}{2} & +\frac{i}{2} & 0 & -1 & -\frac{1}{2} & +i & -i & +\frac{1}{2} & 0\\ 8\cdot P_{2_{4}2_{4}}^{T_{2}} & 1 & 0 & 0 & -1 & 1 & 0 & -1 & -1 & 0 & 1 \end{array}$$

Without evaluating Equation (122), one may find ten *O*⊃*C*_4_ sub-classes by simply inspecting Figure 22 for operations in each *O*-class that transform into each other by *C*_4_ operations **R***_z_^p^only*. The *O*-class of eight 120° rotations **r***_k_* split into two sub-classes, one [*r*_1_*, r*_2_*, r̃*_3_*, r̃*_4_] whose axes intersect four corners of the +*z* front square, and the other [*r̃*_1_*, r̃*_2_*, r*_3_*, r*_4_] whose axes similarly frame the −*z* back square. The class of six diagonal 180° rotations **i***_k_* split into a sub-class [*i*_1_*, i*_2_*, i*_5_*, i*_6_] whose two-sided axes bisect edges of the ?*z* squares, and sub-class [*i*_3_*, i*_4_] whose axes are perpendicular to *z*-axis and bisect edges of ?*xy* side squares. The 180° rotational class [*ρ_x_, ρ_y_, ρ_z_*] splits similarly into sub-classes [*ρ_x_, ρ_y_*] and [*ρ_z_*] with axes perpendicular and along, respectively, the **R***_z_* axis. The 90° class splits, as indicated in the top row of Equation (122), into a sub-class of four perpendicular *xy*-axial rotations and separate sub-classes for *R_z_* and *R̃_z_*.

The inverse to Equation (121) expresses the ten subclasses in terms of the ten diagonal irrep projectors using the same (albeit, conjugated) array of *D_n_*__4__*_n_*__4__*^α^* (*g_k_*). However, column and row labels must switch and acquire different coefficients.

(123) $$\frac{c_{k}}{{}^{\circ}c_{k}}=\sum_{k=0}^{10}D_{n_{4}n_{4}}^{\alpha }(g_{k})P_{n_{4}n_{4}}^{\alpha }=\sum_{k=0}^{10}\frac{D_{n_{4}n_{4}}^{\alpha }(c_{k})}{{}^{\circ}c_{k}}P_{n_{4}n_{4}}^{\alpha }$$

#### 7.1.2. Resolving D-matrices with *C*_4_ Local Symmetry

Off-diagonal *D_m_*__4__*_n_*__4__*^α^* (*g_k_*) matrices derive from products of diagonal irrep projectors in Equation (122) using Equation (82b) repeated here.

(124) $$P_{j,j}^{\alpha }\cdot g\cdot P_{k,k}^{\alpha }=D_{j,k}^{\alpha }(g)P_{j,k}^{\alpha }$$

Scalar *A*_1_ and pseudo-scalar *A*_2_ are given first then *E*, *T*_1_, and *T*_2_ irrep matrices for the fundamental **i***_k_*-class of *O*.

(125) $$\begin{array}{l} D_{0_{4}0_{4}}^{A_{1}}(i_{k}i_{\text{k}})=i_{1}+i_{2}+i_{3}+i_{4}+i_{5}+i_{6}\\ D_{2_{4}2_{4}}^{A_{2}}(i_{k}i_{\text{k}})=-(i_{1}+i_{2}+i_{3}+i_{4}+i_{5}+i_{6}) \end{array}$$

(126) $$D^{E}(i_{k}i_{\text{k}})=\begin{array}{ccc} & 0_{4} & 2_{4}\\ 0_{4} & -\frac{1}{2}(i_{1}+i_{2}+i_{5}+i_{6})+i_{3}+i_{4} & \frac{\sqrt{3}}{2}(i_{1}+i_{2}-i_{5}-i_{6})\\ 2_{4} & h.c. & \frac{1}{2}(i_{1}+i_{2}+i_{5}+i_{6})-i_{3}-i_{4} \end{array}$$

(127) $$\begin{array}{l} \begin{array}{cccc} D^{T_{1}^{*}}(i_{k}i_{\text{k}}) & 1_{4} & 3_{4} & 0_{4}\\ 1_{4} & -\frac{1}{2}(i_{1}+i_{2}+i_{5}+i_{6}) & -\frac{1}{2}(i_{1}+i_{2}-i_{5}-i_{6})-i(i_{3}-i_{4}) & -\frac{1}{\sqrt{2}}(i_{1}-i_{2})+\frac{i}{\sqrt{2}}(i_{5}-i_{6})\\ 3_{4} & h.c. & -\frac{1}{2}(i_{1}+i_{2}+i_{5}+i_{6}) & +\frac{1}{\sqrt{2}}(i_{1}-i_{2})+\frac{i}{\sqrt{2}}(i_{5}-i_{6})\\ 0_{4} & h.c. & h.c. & -(i_{3}+i_{4}) \end{array}\\ \begin{array}{cccc} D^{T_{2}^{*}}(i_{k}i_{\text{k}}) & 1_{4} & 3_{4} & 2_{4}\\ 1_{4} & +\frac{1}{2}(i_{1}+i_{2}+i_{5}+i_{6}) & +\frac{1}{2}(i_{1}+i_{2}-i_{5}-i_{6})-i(i_{3}-i_{4}) & +\frac{1}{\sqrt{2}}(i_{1}-i_{2})+\frac{i}{\sqrt{2}}(i_{5}-i_{6})\\ 3_{4} & h.c. & +\frac{1}{2}(i_{1}+i_{2}+i_{5}+i_{6}) & -\frac{1}{\sqrt{2}}(i_{1}-i_{2})+\frac{i}{\sqrt{2}}(i_{5}-i_{6})\\ 0_{4} & h.c. & h.c. & +(i_{3}+i_{4}) \end{array} \end{array}$$

Symmetry of *C*_4_⊂*O* subclass [*i*_1_*, i*_2_*, i*_5_*, i*_6_] and [*i*_3_*, i*_4_] would demand equality of parameters for each.

(128) $$i_{1}=i_{2}=i_{5}=i_{6}\equiv i_{1256}\equiv i_{\text{I}},\;\;\;and,\;\;\;i_{3}=i_{4}\equiv i_{34}\equiv i_{\text{II}}$$

Setting each parameter to the inverse of its sub-class order (*i_k_*=1/(°*c_i_*_*_k_*_)) reduces each matrix to diagonal form and gives the diagonal *D_n_*__4__*_n_*__4__*^α^* (*g_k_*) given in Equation (122). Classes *r*, *ρ*, *R* behave similarly.

#### 7.1.3. Resolving Hamiltonians with *C*_4_ Local Symmetry

**Figure 25:**
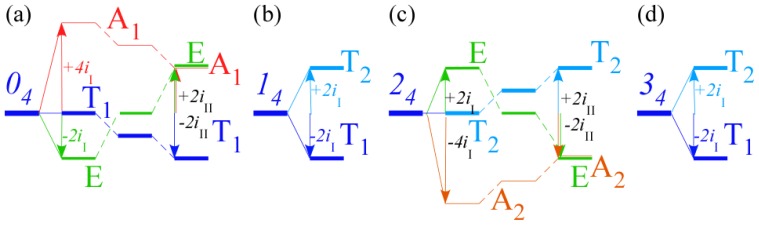
*O i*-class level clusters of *C*_4_ local symmetry (**a**) 0_4_; (**b**) 1_4_; (**c**) 2_4_; (**d**) 3_4_.

An octahedral Hamiltonian **H** = ∑*_k_*_=1_^24^*g_k_***ḡ***_k_* with local *C*_4_(*z*) symmetry is resolved by sorting *g_k_* into its *C*_4_(*z*) sub-classes *c_k_* and then into **P***_n_*__4__*_n_*__4__*^α^* whose coefficients are the desired **H** eigenvalues *ε_n_*__4__*^α^*. Zero off-diagonal *H_m_*__4__*_n_*__4__*^α^* = 0 and *C*_4_-*local symmetry conditions* shown in Equation (128) arise from Equation (122) consistent with Figure 22. Tunneling parameter *i*_1256_=*i*_I_ from +*z*-axis to its 1*^st^*-neighbor ±*x* or ±*y* axes may dominate flip-tunneling *i*_34_ = *i*_II_ to 2*^nd^* neighbor-*z*-axis. The *i*-columns of Equation (122) (or matrix diagonals in Equations (125)–(127)) give *i*_I_ and *i*_II_ contributions to eigenvalues *ε_n_*__4__*^α^* listed in the *i_n_*-column of Table 11. Clusters (*ε*_0_4__*^A^*^_1_^, *ε*_0_4__*^T^*^_1_^, *ε*_0_4__*^E^*) through (*ε*_3_4__*^T^*^_2_^, *ε*_3_4__*^T^*^_1_^) are plotted in Figure 25 for select values of parameters *i*_I_ = *i*_1256_ and *i*_II_ = *i*_34_.

**Figure 26:**
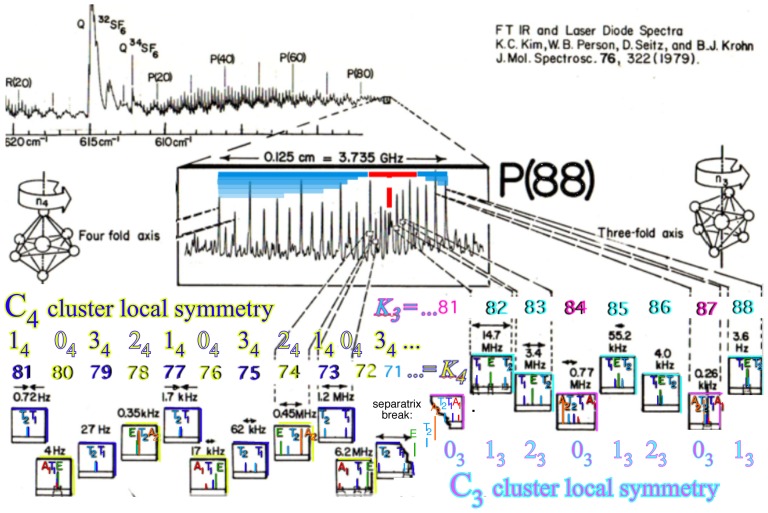
Excerpts of *SF*_6_*ν*_4_*P*(88) superfine spectral cluster structure in 16*μm* region (Missing: *K*_4_=82...88).

One expects the parameter *i*_II_ for 2*^nd^*-neighbor tunneling to be exponentially smaller than *i*_I_ for adjacent tunneling so the (*i*_II_ = 0)-cases are drawn first in Figure 25. While the *i*-class operations are most fundamental (all operations are generated by products of **i***_k_*) other operations also generate 1*^st^*-neighbor transformation. Three class parameters *R_xy_*(90°), *r*_I_(120°), and *i*_I_(180°) label 1*^st^*-neighbor inter-*C*_4_ axial tunneling paths that have the same *i*_I_-level patterns and splitting ratios as (*i*_II_=0)-cases in Figure 25 but with differing sign. (Signs differ since each sub-class eigenvalue set must be orthogonal to all others as shown below.) Level patterns in Figure 25 are reflected in *spectral* patterns of Figure 26 if both ground and excited vibe-rotor states have similar RES-shape. However, only *C*_4_*_z_* sub-class *i*_I_(180°) patterns (with *i*_I_*<* 0) exhibit spectral ordering (*A*_1_*T*_1_*E*)(*T*_2_*T*_1_)(*ET*_2_*A*_2_)(*T*_2_*T*_1_) on the left hand side of Figure 26 that is maintained even as levels re-cluster into patterns (*T*_1_*ET*_2_)(*T*_1_*ET*_2_)(*A*_2_*T*_2_*T*_1_*A*_1_) of *C*_3[111]_ local symmetry across the separatrix break on the right-hand side of Figure 26 as analyzed below [8,37]. *O*-crystal-field wavefunctions for either case tend to follow a Bohr-orbital progression *s*(*A*_1_)*, p*(*T*_1_)*, d*(*E, T*_2_)*, f*(*T*_1_*, A*_2_*, T*_2_)*, g*(*E, T*_1_*, T*_2_*, A*_1_)*, ..*. In general, ordering is sensitive to RES-shape and tensor rank as discussed later.

For an isolated three-level (*ATE*)-cluster of local symmetry 0_4_ or else 2_4_ the splitting pattern requires only two parameters. This could be either the 180°(*i*_I_, *i*_II_) or the 90°(*R_xy_*, *R_z_*) class pair in Table 11. The 120°-class, lacking 180° flips, has just one real parameter *r*_I_. Parameters *i*_I_, *R_xy_*, and *r*_I_ each split (*ATE*) by 2:1 ratio but differ in sign.

Local symmetry 1_4_ and 3_4_ each have two-level (*TT*) clusters that require just one splitting parameter, say *i*_I_, or else *R_xy_*. Complex parameters *R_z_* and *I_z_* of the 90° *R_n_*-class and the *ρ_n_*(180°)-class in Table 11 may play minor roles in most *C*_4_ clusters but are necessary in order that the whole set be orthonormal and complete.

#### 7.1.4. Orthogonality-Completeness of Local Symmetry Parameters

Equation (122) expands **P***_nn_*^(^*^α^*^)^ by Equation (83) in group operators (**1***,***r**_1_*,***r**_2_*, ...***i**_6_). It acts on |**1**〉 to give |*_n_*__4__*_n_*__4__^(^*^α^*^)^ 〉 eigenkets in Equation (129).

(129) $$\begin{array}{l} {|}_{nn}^{\left(\alpha \right)}\rangle =P_{nn}^{\left(\alpha \right)}|1\rangle \sqrt{\frac{{}^{\circ}G}{\ell^{\alpha }}}=\sqrt{\frac{\ell^{\alpha }}{{}^{\circ}G}}\sum_{b=1}^{{}^{\circ}G}D_{nn}^{(\alpha )*}(g_{b})g_{b}|1\rangle \\ =\sqrt{\frac{\ell^{\alpha }}{{}^{\circ}G}}\sum_{b=1}^{{}^{\circ}G}D_{nn}^{(\alpha )*}(g_{b})|g_{b}\rangle \end{array}$$

An *O*-symmetric **H** matrix is a sum of *dual* operators (**1̄***,***r̄**_1_*,***r̄**_2_*, ...***r̄**_6_) with coefficients *g_a_*=*ε*_0_*, r*_1_*, r*_2_*, ..., i*_6_. Local symmetry *C*_4_ or *C*_3_ reduces the sum to *ρ_G_*=10 sub-class terms **c̄***_a_*=**ḡ***_a_*+**ḡ**′*_a_* +... each sharing a coefficient *g_a_*=*g*′*_a_* ...

(130) $$H=\sum_{a=1}^{{}^{\circ}G}g_{a}{\bar{g}}_{a}=\sum_{a=1}^{\rho G}g_{a}{\bar{c}}_{a}$$

From these arise expansions like Table 11 of **H** eigenvalues *ε_n_*__4__^α^ in terms of its coefficients *g_a_*. Dual commutation **g***_j_***ḡ***_k_*=**ḡ***_k_***g***_j_* makes **P***_nn_*^(^*^α^*^)^ and **H** commute. Duality relation in Equation (94) leads to a D^α*^-weighted sum of *g_a_* analogous to sum in Equation (129) of |*g_a_*〉.

(131) $$\begin{array}{c} {ɛ}_{n}^{\alpha }=\langle_{nn}^{\left(\alpha \right)}{|H|}_{nn}^{\left(\alpha \right)}\rangle =\langle 1|P_{nn}^{\left(\alpha \right)}HP_{nn}^{\left(\alpha \right)}|1\rangle \frac{{}^{\circ}G}{\ell^{\alpha }}=\langle 1|HP_{nn}^{\left(\alpha \right)}|1\rangle \frac{{}^{\circ}G}{\ell^{\alpha }}=\langle 1|\sum_{a=0}^{{}^{\circ}G}g_{a}{\bar{g}}_{a}\sum_{b=0}^{{}^{\circ}G}D_{nn}^{(\alpha )*}(g_{b})g_{b}|1\rangle \\ =\langle 1|\sum_{a=0}^{{}^{\circ}G}g_{a}\sum_{b=0}^{{}^{\circ}G}D_{nn}^{(\alpha )*}(g_{b})g_{b}g_{a}^{-1}|1\rangle =\sum_{a=0}^{{}^{\circ}G}g_{a}D_{nn}^{(\alpha )*}(g_{a})=\sum_{a=1}^{\rho_{G}}D_{nn}^{(\alpha )*}(g_{a}){}^{\circ}c_{a}g_{a} \end{array}$$

Each *C*_4_ sub-class of order °*c_a_* has °*c_a_* equal terms *g_a_D_nn_*^(^*^α^*^)*^ (*g_a_*) = *g*′*_a_D_nn_*^(^*^α^*^)*^ (*g*′*_a_*) =. . . expanding eigenvalue *ε_n_*__4__*^α^*. Rank-of-group *ρ_G_* = 10 is the number of eigenvalues and of expansion terms °*c_a_g_a_D_nn_*^(^*^α^*^)*^ (*g_a_*) in Equation (131) or Table 11. Each of ten eigenvalues *ε_n_*__4__*^α^*=(*ε^A^*^_1_^*, ε^A^*^_2_^*, ..., ε*_3_4__*^T^*^_2_^ ) expand to ten *C*_4_-local tunneling parameters *g_a_*=(*ε*_0_*, r*_I_*, r*_II_*, ..., i*_II_) and *vice-versa*.

(132) $$\begin{array}{l} g_{a}=\langle 1|H|g_{a}\rangle =\langle 1|Hg_{a}|1\rangle =\sum_{\alpha }\sum_{j}^{\ell^{\alpha }}\sum_{k}^{\ell^{\alpha }}D_{jk}^{\left(\alpha \right)}(g_{a})\;\langle 1|HP_{jk}^{\alpha }|1\rangle \\ =\sum_{\alpha }\sum_{n}^{\ell^{\alpha }}D_{nn}^{\left(\alpha \right)}(g_{a})\;\langle 1|HP_{nn}^{\alpha }|1\rangle =\sum_{\alpha }\sum_{n}^{\ell^{\alpha }}D_{nn}^{\left(\alpha \right)}(g_{a})\frac{\ell^{\alpha }}{{}^{\circ}G}{ɛ}_{n}^{\alpha } \end{array}$$

One might count twelve real parameters in Table 11 since both pairs (*r*_I_,*r̃*_I_) and (*R_z_*, *R̃_z_*) are complex, unlike *R*_I_ = *R̃*_I_, which are real. If *H* is a Hermitian array (*H* = *H*^†^) it should only require ten, the rank of *O*, for its ten distinct real eigenvalues and the parameter pairs must be complex conjugates.

**Figure 27:**
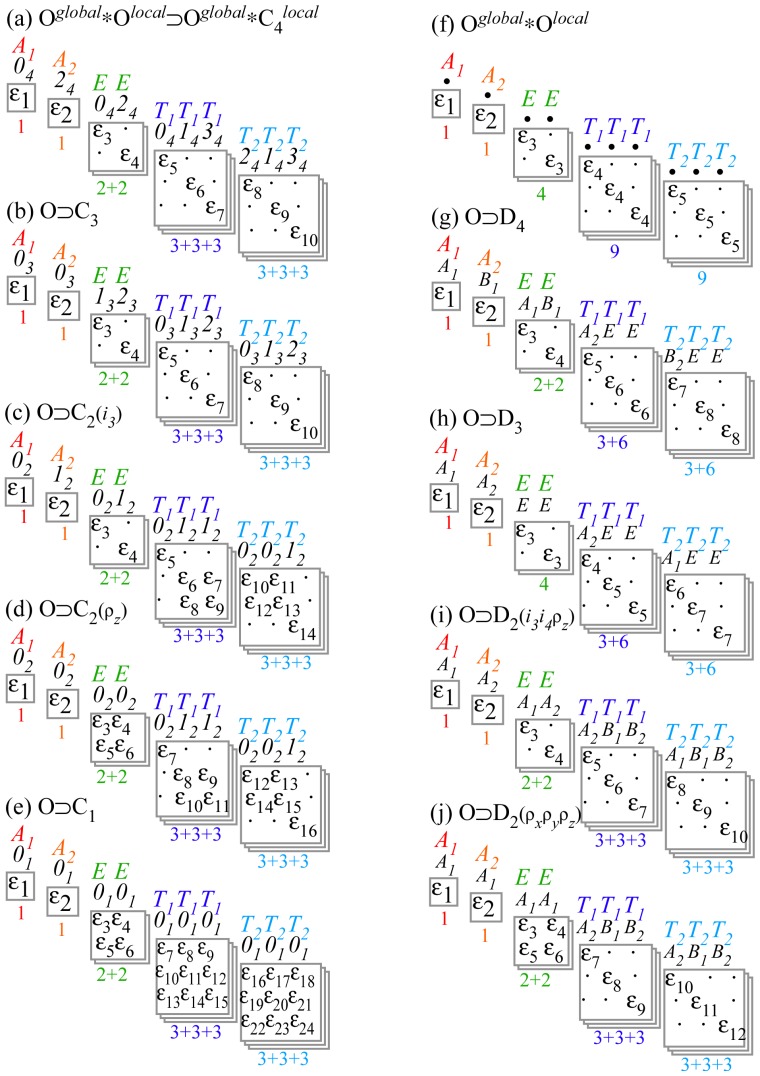
*O*⊃*L*-local symmetry eigenmatrix parameters (a–e) *L*=*C*_4_,...,*C*_1_ (f-j) *L*=*O*,*D*_4_,...,*D*_2_.

With no conjugation symmetry, such as for a *unitary O* ⊃ *C*_4_-symmetric matrix, the *R* and *r* parameters may be complex and unrelated to *R̃* and *r̃*, and resulting extra real parameters are then needed. Symmetry parameter dimension matches eigensolution dimension for each local symmetry as shown in Figure 27.

#### 7.1.5. Resolving Hamiltonians with *C*_3_ Local Symmetry

**Figure 28:**
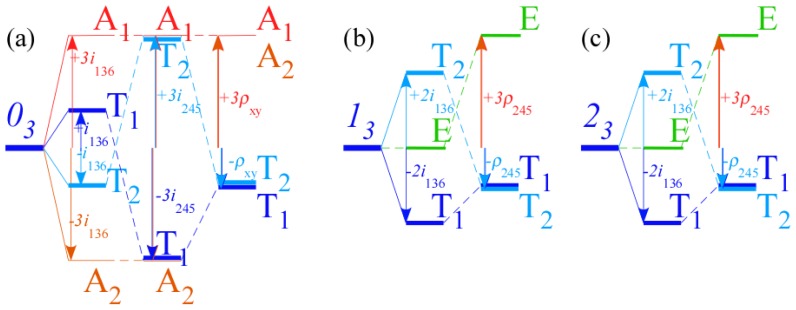
*O i*-class and *ρ*-class level clusters of *C*_3_ local symmetry given different tunneling parameters.

The previous two sections have detailed of symmetry-based level clustering and cluster splitting for *C*_4_. In Figure 26 these are the lower energy clusters of *SF*_6_ for *ν*_4_*P*(88). Given the previous two sections, it is possible to find the splittings of the *C*_3_ sub-group quickly. Starting with Equation (117) and Equation (118) one can build the irreducible representations necessary to create the **P***_n_*__3__*_n_*__3__*^α^* for the new sub-group. At this point, one can create a table analogous to Table 11. Such a table for *C*_3_ is shown in Table 12. The *C*_3_ clustering fits patterns of (*A*_1_*, A*_2_*, T*_2_*, T*_2_) and two of (*E, T*_1_*, T*_2_), each with a total degeneracy of 8. As before in Figure 25, the splittings in *C*_3_ make different patterns depending on which tunneling parameters are active. This is demonstrated in Figure 28.

#### 7.1.6. Octahedral Splitting for a Range of Local Symmetry *C*_1_⊂*C*_2_...⊂*O*

As the order °*L* of local symmetry *L*⊂*G* decreases there are proportionally fewer types of local symmetry irrep *d^λ^*(*L*) and hence fewer types of energy level cluster since each cluster is defined by its induced representation *d^λ^*(*L*)↑*G*. There is a proportional increase in total number *ℓ^λ↑G^*=(*ℓ^λ^*)°*G*/° *L* of levels in each eigenvalue cluster. However, *G*-symmetry degeneracy limits the total number of *distinct* eigenvalues from all clusters to be global rank *ρ*(*G*) or less, no matter what local symmetry is in effect. Octahedral rank is *ρ*(*O*)=10=*ℓ^A^*^_1_^+*ℓ^A^*^_2_^+*ℓ^E^*+*ℓ^T^*^_1_^+*ℓ^T^*^_2_^ where *ℓ^α^* gives both the global degeneracy of each level type and the number of times it appears.

The number of *H*-matrix parameters equals the number of distinct eigenvalues as long as all eigen*vectors* are determined by global-local symmetry, that is, each entry is 0 or 1 in the *G*⊃*L* correlation array. Diagonal eigenmatrix forms are shown in Figure 27a,b for *C*_4_⊂*O* and *C*_3_⊂*O* for which all bases states are distinctly labeled. Multiple correlation (≥ 2) occurs if *L*-symmetry is too small to determine some of the °*G* eigenbases. Then the *H*-matrix must have extra parameters that fix vectors through diagonalization.

This happens for the *C*_2_(**i**_1_) ⊂*O* symmetry whose correlation array in Equation (117) assigns the same *C*_2_ label to two bases of *T*_1_ and of *T*_2_. (Two *C*_2_ symmetries 0_2_ and 1_2_ cannot distinctly label three bases.) Figure 22 shows *C*_2_(**i**_1_) splits *O* into fourteen sub-classes: (**1**), (**r**_1_**r̃**_4_), (**r**_2_**r̃**_2_), (**r**_3_**r̃**_3_), (**r**_4_**r̃**_1_), (*ρ_x_ρ_z_*), (*ρ_y_*), (**R***_x_***R***_z_*), (**R̃***_x_***R̃***_z_*), (**R***_y_***R̃***_y_*),(**i**_1_), (**i**_2_), (**i**_3_**i**_5_), (**i**_4_**i**_6_). The *C*_2_⊂*O* sub-classes form a non-commutative algebra and cannot be resolved so easily as *C*_3_⊂*O* or *C*_4_⊂*O* into commuting idempotent combinations like Equation (123).

Spectral resolution of fourteen *C*_2_(**i**_1_)⊂*O* sub-classes requires more than rank number *ρ*(*O*)=10 of diagonal commuting *O* idempotents **P***_nn_^α^*. To fully determine *C*_2_ basis, two off-diagonal pairs **P***_ab_^T^*^_1_^=**P***_ba_^T^*^_1_†^ and **P***_ab_^T^*^_2_^=**P***_ba_^T^*^_2_†^ of non-commuting nilpotent projectors are needed to finish *C*_2_-labeling of *T*-triplets. Adding these four gives fourteen projectors with their fourteen parameter coefficients *ε_ℓ_* shown in Figure 27c to fully define general *C*_2_(**i**_1_)⊂*O H*-operators. (However, only twelve of the fourteen parameters are independent for Hermitian *H_a,b_*=H_b,a_^*^.)

The other class of *C*_2_ symmetry has similar problems. Local *C*_2_(*ρ_z_*)⊂*O* symmetry requires projector pairs **P***_ab_^T^*^_1_^=**P***_ba_^T^*^_1_†^ and **P***_ab_^T^*^_2_^=**P***_ba_^T^*^_2_†^, too, but then another nilpotent pair **P***_ab_^E^*=**P***_ba_^E^*^†^ must be added to label repeated *E* bases in array Equation (117). This gives sixteen *C*_2_(*ρ_z_*) sub-classes to resolve and sixteen parameters sketched in Figure 27d. (Hermitian *H*=*H*^†^ matrices for *C*_2_(*ρ_z_*)⊂*O* have thirteen free parameters.)

For the lowest local symmetry *C*_1_=[**1**] (*i.e*., no local symmetry) sub-classes are completely split since every *O*-operator is invariant to **1** as *C*_1_ provides no distinguishing labeling, and all twenty-four *O*-projectors (∑*_α_*(*ℓ^α^*)^2^=24) are active in its resolution. The 24-parameter*H*-matrix resolution is sketched in Figure 27e. Each parameter *ε_a_* for *a*=1*, ...,* 24 is a combination of 24 products D_j,k_^α^^*^(*g_p_*)*g_p_* (*p*=1*, ...,* 24) of irrep and group element coefficient *g_p_* as given in Equation (90) or (131). (If *H* is Hermitian the number of free parameters reduces to ∑*_α_ℓ^α^*(*ℓ^α^*+1)=17.)

For *O*’s highest local symmetry, namely *O* itself, there is no splitting of the ∑*_α_*( *ℓ^α^*)^0^=5 invariant idempotents **P***^α^* that resolve the five *O* classes. Then *H* has five independent parameters and five eigenvalues of degeneracy (*ℓ^α^*)^2^. This 5-parameter resolution is sketched in Figure 27f. Total level degeneracy for sub-matrix eigenvalues are listed below each one, and show less splitting than Abelian cases listed in Figure 27a–e.

Any non-Abelian local symmetry such as *L* = *D*_4_ also fails to split **P***^α^* into a full number *ℓ^α^* of components **P***_nn_^α^* if *O* irrep-(*α*) correlates with multi-dimensional *L*-irreps. By splitting out less than the full rank number *ρ*(*O*)=10 of idempotent projectors **P***_nn_^α^*, the resulting number of independent *H* matrix parameters reduces accordingly. The 8-parameter resolution for an *H*-matrix with *D*_4_⊂*O* is sketched in Figure 27g and similarly for *D*_3_⊂*O* in Figure 27h. Two kinds of *D*_2_⊂*O* in Figure 27i,j share degeneracy sums with the Abelian cases.

Each matrix display lists *exact* degeneracy *ℓ^α^* due to *global* symmetry *O* but not the cluster *quasi*-degeneracy *ℓ^λ^*^↑^*^G^* due to *local* symmetry induced representation *d^λ^*(*L*)↑*G*. The latter is found by summing global degeneracy *ℓ^α^* of all states |*_a,λ_^α^*〉 with the same local symmetry *λ* as per Frobenius reciprocity in Equation (106). The result is integer *ℓ^λ^*^↑^*^G^*=(*ℓ^λ^*)°*G*/° *L* mentioned above.

## 8. Spectral Resolution of full *O_h_* Symmetry

Including inversion **I** and reflection operations *σ_n_* allows parity correlations between even-*g* (*gerade*) and odd-*u* (*ungerade*) states. Two classes of *C*_2_ subgroups lie in *O* and appear in separate *C*_2_-correlations in Equation (117). In the following *O_h_* correlations Equation (133), the two types of *C*_2_*_v_* subgroups have separate tables. The first subgroup *C*_2_*_v_^i^*=[**1***, σ_y_,***i**_1_*, σ*_2_] is the one of the three local symmetries shown in Figure 12 while the second *C*_2_*_v_^z^*=[**1***, ρ_z_, σ_y_, σ_x_*] is just a subgroup of local symmetry *C*_4_*_v_* as would be *C*_2_*_v_*^34^=[**1***, ρ_z_, σ*_3_*, σ*_4_].

(133) $$\begin{array}{c} \begin{array}{cccccc} O_{h}↓C_{4v} & A^{\prime } & B^{\prime } & A^{\prime \prime } & B^{\prime \prime } & E\\ A_{1g}↓C_{4v} & 1 & \cdot & \cdot & \cdot & \cdot \\ A_{2g}↓C_{4v} & \cdot & 1 & \cdot & \cdot & \cdot \\ E_{g}↓C_{4v} & 1 & 1 & \cdot & \cdot & \cdot \\ T_{1g}↓C_{4v} & \cdot & \cdot & 1 & \cdot & 1\\ T_{2g}↓C_{4v} & \cdot & \cdot & \cdot & 1 & 1\\ A_{1u}↓C_{4v} & \cdot & \cdot & 1 & \cdot & \cdot \\ A_{2u}↓C_{4v} & \cdot & \cdot & \cdot & 1 & \cdot \\ E_{u}↓C_{4v} & \cdot & \cdot & 1 & 1 & \cdot \\ T_{1u}↓C_{4v} & 1 & \cdot & \cdot & \cdot & 1\\ T_{2u}↓C_{4v} & \cdot & 1 & \cdot & \cdot & 1 \end{array},\;\;\;\begin{array}{cccc} C_{3v} & A^{\prime } & A^{\prime \prime } & E\\ A_{1g} & 1 & \cdot & \cdot \\ A_{2g} & \cdot & 1 & \cdot \\ E_{g} & \cdot & \cdot & 1\\ T_{1g} & \cdot & 1 & 1\\ T_{2g} & 1 & \cdot & 1\\ A_{1u} & \cdot & 1 & \cdot \\ A_{2u} & 1 & \cdot & \cdot \\ E_{u} & \cdot & \cdot & 1\\ T_{1u} & 1 & \cdot & 1\\ T_{2u} & \cdot & 1 & 1 \end{array}\\ \begin{array}{ccccc} C_{2v}^{i} & A^{\prime } & B^{\prime } & A^{\prime \prime } & B^{\prime \prime }\\ A_{1g} & 1 & \cdot & \cdot & \cdot \\ A_{2g} & \cdot & 1 & \cdot & \cdot \\ E_{g} & 1 & 1 & \cdot & \cdot \\ T_{1g} & \cdot & 1 & 1 & 1\\ T_{2g} & 1 & \cdot & 1 & 1\\ A_{1u} & \cdot & \cdot & 1 & \cdot \\ A_{2u} & \cdot & \cdot & \cdot & 1\\ E_{u} & \cdot & \cdot & 1 & 1\\ T_{1u} & 1 & 1 & \cdot & 1\\ T_{2u} & 1 & 1 & 1 & \cdot \end{array},\;\;\;\begin{array}{ccccc} C_{2v}^{z} & A^{\prime } & B^{\prime } & A^{\prime \prime } & B^{\prime \prime }\\ A_{1g} & 1 & \cdot & \cdot & \cdot \\ A_{2g} & 1 & \cdot & \cdot & \cdot \\ E_{g} & 2 & \cdot & \cdot & \cdot \\ T_{1g} & \cdot & 1 & 1 & 1\\ T_{2g} & \cdot & 1 & 1 & 1\\ A_{1u} & \cdot & \cdot & 1 & \cdot \\ A_{2u} & \cdot & \cdot & 1 & \cdot \\ E_{u} & \cdot & \cdot & 2 & \cdot \\ T_{1u} & 1 & 1 & \cdot & 1\\ T_{2u} & 1 & 1 & \cdot & 1 \end{array} \end{array}$$

The local symmetry *C*_2_*_v_^i^*⊂*O_h_* unambiguously defines all states in its correlation array while the other *C*_2_*_v_* symmetries fail to split the *E_g_* and *E_u_* sub-species. The former lead to complete eigenvalue formulae. The latter may not.

### 8.1. Resolving Hamiltonians with C_2v_ Local Symmetry

As the order of the local sub-group symmetry goes down, the degeneracy and complexity of the rotational cluster must increase. *O_h_*⊃*C*_2_*_v_* clusters are 12 fold degenerate and come in 4 cluster species. Matrices describing this system are larger, but *O*⊃*C*_2_ will show many of the same effects. To actually resolve the doubled *T*_1_ or *T*_2_ triplets of *O*⊃*C*_2_ requires distinguishing the *u* and *g* versions of each. The *C*_2_ clusters are 12 fold degenerate, but they are also easily displayed.

As noted earlier, *O*⊃*D*_3_⊃*C*_2_ and *O*⊃*D*_4_⊃*C*_2_ local symmetries give identical cluster degeneracies and groupings, but with cluster splittings and structure dependent on the sub-group chain. Though it neglects inversion, Figure 27 indicates that there are several different types of *O*⊃*C*_2_ (and, thus *O_h_*⊃*C*_2_*_v_* local sub-group symmetries). Examples given here involve the *O* ⊃*D*_4_⊃*C*_2_(*i*_4_) sub-group chain.

Compared with *O*⊃*C*_4_ and *O*⊃*C*_3_, the splittings of *O*⊃*C*_2_ are relatively simple to calculate since the terms in Equation (131) will be real. Creating splitting tables for *C*_2_ is done in the same way as for Tables 11 and 12. It is shown in Table 13.

#### 8.1.1. Local Sub-Group Tunneling Matrices and Their Inverse

Table 13 can be further broken apart to demonstrate how one can create an automated process to evaluate the tunneling splittings for *O*⊃*C*_2_ local-symmetry structures. What will result is a transformation between cluster-splitting energy and tunneling parameters. The inverse of this transformation is also easily defined.

Equation (131) produces Table 13, but even after combining splittings from each subclass, repetition exists. We show the two steps to convert Table 13 into the transformation matrix just described. First we assume that only *n_m_* levels may interact with themselves, e.g., that a 1_2_ cluster may not interact with a 0_2_ cluster. Second we recognize that only half of the subclasses are needed to fully define the possible splittings, the others simply repeat the same information. Table 13 shows this for the 0_2_ cluster. Looking at the *A*_1_ level in the 0_2_ cluster, one can see that the subclasses **1***, r_n_, ρ_n_* make a vector {1*,* 4*,* 4*,* 2*,* 1} while the *R_n_, i_n_* subclasses make a vector {4*,* 2*,* 4*,* 1*,* 1}. These vectors are reordered versions of each other. Thus only one is needed. The *A*_2_ level in the 1_2_ cluster shows the same similarity, but the *R_n_, i_n_* now contain a negative sign.

By using only the minimum number of splitting parameters and including only a single cluster gives a condensed version of Table 13 that acts as a transformation that inputs symmetry-based tunneling values and outputs energy levels. Such a table is shown in Table 14. A simple inverse of the matrix in Table 14 will produce the transformation giving tunneling parameters for a given set of cluster energy splittings, as shown in Table 15.

There are multiple ways to use Tables 14 and 15. Among the most useful is to use the columns of Table 14 as a predictor of possible splitting patterns. Using the inverse matrix to find spectroscopic tunneling parameters from cluster splittings may also become a useful and automated process.

An example demonstrates this process for a model (4*,* 6)-octahedral-Hecht spherical-top Hamiltonian Equation (134) with varying spectroscopic parameters. The terms *T*^[4]^ and *T*^[6]^ model rotational distortions written in an octahedral basis of fourth and sixth order respectively in *J*. The parameter *θ* is varied to explore the different relative contributions of *T*^[4]^ and *T*^[6]^ while keeping them normalized. Because *T*^[4]^ and *T*^[6]^ each have octahedral symmetry, Equation (134) represents all possible octahedral pure rotational Hamiltonians up to sixth order.

(134) $$H=BJ^{2}+\text{cos}(\theta )T^{\left[4\right]}+\text{sin}(\theta )T^{\left[6\right]}$$

**Figure 29:**
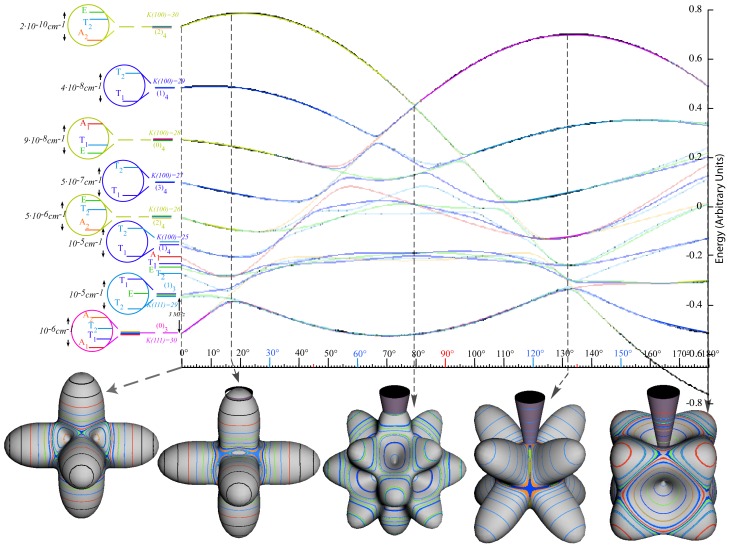
*J*=30 Energy levels and RES plots for *T*^[4^*^,^*^6]^*vs.* [4,6] mix-angle *θ* with *T*^[4]^ levels above *φ*=0° (extreme left), *T*^[6]^ levels at *θ*=90° (center), and −*T*^[4]^ levels at *θ*=180° (extreme right). *C*_4_ local symmetry and 6-fold level clusters dominate at *θ*=17° while *C*_3_ type 8-fold level clusters dominate at *θ*=132°. In between these extremes are *C*_2_ type 12-fold level clusters particularly around *θ*=80° where a *C*_3_ – *C*_4_ level-cluster-crossing of the top 14 levels occurs.

As noted in Section 3 cluster structure location and the RES shape will change significantly as the Hamiltonian parameters change in Equation (134) as Figure 29 (a copy of Figure 6) shows by plotting rotational energy levels of Equation (134) for changing *θ* with corresponding RES at points along the *θ* axis. RES plots in the figure demonstrate how the phase-space changes as *θ* varies.

**Figure 30:**
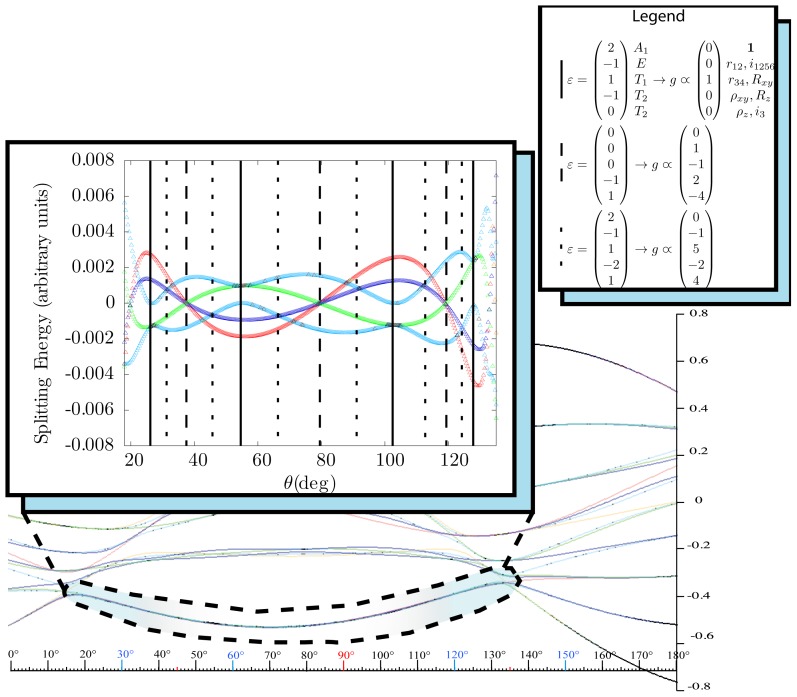
The plot focuses on the lowest 0_2_(*C*_2_)↑*O* cluster in the previous energy plot (Figure 29) of the *T*^[4^*^,^*^6]^ Hamiltonian for *J* = 30. The inside plot has been magnified 100 times. The inside diagram also centers the levels around their center-of-energy, showing only the splittings and ignoring the shifts of the cluster. Symmetry species are colored as before: *A*_1_: red, *A*_2_: orange, *E*_2_ : green, *T*_1_: dark blue, and *T*_2_: light blue. The vertical lines on inside plot draw attention to specific clustering patterns described in the text. 1_2_(*C*_2_)↑*O* clusters have similar superfine structure but with *A*_2_ replacing *A*_1_ and *T*_1_ switched with *T*_2_.

RES diagrams in Figure 29 along with the cluster degeneracy indicate where in the parameter-space *C*_2_ clusters exist. The lowest 0_2_(*C*_2_)↑*O* cluster in Figure 29 for *θ* between 18° and 132° labels a kaleidoscope of 12 waves each with *C*_2_ local symmetry. Its superfine levels are magnified about 100 times in the central inside plot of Figure 30 which has been adjusted to show level splittings but not whole cluster shifting. (The *θ*-dependent cluster center-of-energy is subtracted.) The locally antisymmetric 1_2_(*C*_2_)↑*O* clusters contain quite similar superfine structure but with *A*_2_ replacing *A*_1_ and *T*_1_ switched with *T*_2_.

At certain *θ*-points in Figure 30 levels of different symmetry cross and one of three distinctive splitting patterns emerge. These points occur periodically as indicated by vertical lines that are (starting form left side) solid, dotted, dashed, dotted, solid, dotted, dashed, solid, and so forth across the plot. The three distinctive *ε^α^*-energy level patterns for species *α*=(*A*_1_*, E, T*_1_*, T*_2_*, T*_2_) are given by vectors *ε_dash_*=(0*,* 0*,* 0*,* 1*,*−1), *ε_dot_*=(2*,* −1*,* 1*,* 1*,* −1) and *ε_solid_*=(2*,* −1*,* 1*,* 0*,* −1), respectively. These repetitious patterns seem to persist even outside of the marked-off sections to the very ends of the *C*_2_ cluster region at *θ*≃18° and *θ*≃132° where they grow slightly but maintain their respective superfine ratio patterns and degeneracy. The matrix in Table 15 transforms each of the three *ε^α^*-vectors in Figure 30 into a vector of *O*-defined sub-class tunneling amplitudes *g_r_*. These are evaluated for clusters at several values of parameter *θ* used in *T*^[4^*^,^*^6]^ Hamiltonian Equation (134). Proportioned values of the tunneling amplitudes *g_r_* for the three distinctive cases are listed in the inset legend of Figure 30 based on Tables 14 and 15.

Dotted-line and solid-line curve patterns appear alternately flipped in sign. Dotted-line patterns have a crossing (*T*_1_,*T*_2_) pair while the solid-line patterns have a crossing (*T*_2_,*E*) pair.

Solid-line patterns appear to be centered on quasi-hyperbolic avoided-level-crossing episodes involving the pair of repeated *T*_2_ tensor species of *O*. The ordering (*A*_1_*, T*_1_*, T*_2_*, E, T*_2_) of solid-line superfine level patterns reflects Bohr-like orbital ordering (*s, p, d, f, ..*) of orbital momentum and occurs only when there is just one non-zero sub-class of tunneling parameter, namely that of sub-class (*r*_34_ or equivalent *R_xy_*) that affects tunneling between nearest-neighbor *C*_2_ valleys.

Dashed-line pattern level curve *slopes* appear to alternate (+) and (−) signs and exhibit maximum separation of repeated *T*_2_-species surrounding a degenerate (*A*_1_*, T*_1_*, E*)-sextet crossing midway in between. Such triple-point crossings are quite remarkable. They appear repeatedly in Figure 30 and persist even at low-*J* as seen for *J*=4 in Figure 8c. Higher 1_2_(*C*_2_)↑*O* clusters show similar triple points made of (*A*_2_*, T*_2_*, E*)-sextets.

Such crossings are quite ironic if we recall that it was (*A*_1_*T*_1_*E*), (*A*_2_*T*_2_*E*), and (*T*_1_*T*_2_) clusters noted by Lea, Leask, and Wolf [20] and later Dorney and Watson [21] that led to a theory involving induced representations *K*_4_(*C*_4_)↑*O* including 0_4_(*C*_4_)↑*O*=*A*_1_⊕*T*_1_⊕*E*, 2_4_(*C*_4_)↑*O*=*A*_2_⊕*T*_2_⊕*E*, and ±1_4_(*C*_4_)↑*O*=*T*_1_⊕*T*_2_. (Recall *C*_4_ columns of Equation (117) and reciprocity Equation (106)). This theory uses an inter-*C*_4_-axial tunneling model [22,23] with a single *ad.hoc.* tunneling parameter that predicts a 2:1-splitting ratio for (*ATE*) clusters. *C*_4_-axial tunneling cluster splitting dies exponentially as body momentum-*K* approaches *J* (Recall Figure 26) and thus *C*_4_(*ATE*) levels never actually cross.

However, the *C*_2_(*ATE*) levels in Figure 30 clearly do so and with quite the opposite 1:2-spltting ratio. It is ironic that the more elegant ortho-complete multi-path tunneling models, while useful in exposing these crossings, seem at a loss to explain them, particularly given that they were first noted by Lea, Leask, and Wolf so very long ago!

It would be easy to write off such (*ATE*) triple-crossings and particularly the (*T*_1_*T*_2_) or (*ET*) double-crossings as “accidental” degeneracy. Indeed, all but the latter occur for special values of a complete set of sub-class parameters. However, Figure 30 clearly shows that each type of crossing belong to a periodic structure that is unlikely to be just an accident.

Clearly there is still much to learn about multi-path tunneling models in general and the octahedral ones in particular. Here we can only offer a potentially elegant way to treat these kinds of high-symmetry cases.

## 9. Examples of Rovibronic Energy Eigenvalue Surfaces (REES) and J-Clusters

Semiclassical treatment of rovibronic or rovibrational states provides some insight into the transition between lab-coupled and body-coupled vibronic momentum that are related in Equation (8) through Equation (10b) of Sections 1 and 2. The first semiclassical analysis of fundamental coupling in high-*J* octahedral molecules was done for *ν*_2_*E* [38] and *ν*_3_*T*_1_ [39] bands in 1978 and for overtone *ν*_2_ + *ν*_3_ “hot-bands” in 1979 [40].

These methods are similar in philosophy to those described in Section 2 that approximate tensor eigenvalues with Legendre formulas and thereby construct rotational energy based on a semiclassical **J**-vector. However, the more general approach differs in that it builds an *N*-by-*N* matrix of such formulas that takes account of quantum rovibronic coupling between *N* vibronic (or vibrational) states, that is, a 2-by-2 matrix for the *ν*_2_*E* system, a 3-by-3 matrix for the *ν*_3_*T*_1_ system, and a 5-by-5 matrix for the *ν*_2_ + *ν*_3_ system.

The resulting *N* eigenvalues provide points on *N* nested Rovibronic Energy Eigenvalue Surfaces (REES) for each direction of the semiclassical **J**-vector. Visualization of *P*, *Q*, and *R* state mixing in *ν*_3_*T*_1_ bands by 3-sheet REES was done using the high-resolution 3D-graphics at Los Alamos in 1987 and reported in 1988 [25]. Interesting features of the *ν*_3_*T*_1_ REES include conical intersections that occur for zero scalar Coriolis coupling. These are analogous to well known conical intersections of Jahn–Teller PES that lend insight into BOA breakdown of single adiabatic surfaces. The following contains two examples of REES models. The first is a simplified internal rotation model involving a 2-sheet REES, and the second is an excerpt of a recent study of the *ν*_3_*/*2*ν*_4_ dyad of *CF*_4_ that involves a 9-sheet REES.

### 9.1. Rotor-With-Gyro Model of Internal Rotation

A first application by Ortigosa and Hougen [17] of REES to visualize molecules with internal rotation is related to a simple rotor-with-gyro model [25,41] based on the three lowest rank tensors possible, namely the scalar (rank-0), the vector (rank-1), and the tensor (rank-2). The prolate symmetric top RES in Figure 1 is an example of a scalar-tensor combination. A vector RES lacks **J**-inversion symmetry, that is, time reversal symmetry, so it is forbidden for normal molecules that have no intrinsic dynamic chirality such as embedded spin **S**. We consider how to include an **S** in a way that preserves overall *T* symmetry.

Total momentum **J**=**R**+**S** is the sum of rotor momentum **R** and gyro spin **S. J** is conserved in lab frame but **R** and **S** are not. If gryo is body-frame-fixed by frictionless bearing then rotor gyro-coupling does no work and is an ignorably constant *H_RS_*. **S** and |**J**| are conserved in body frame but **J** and **R** are not.

(135) $$H_{R+S(bod-fixed)}=AR_{x}^{2}+BR_{y}^{2}+CR_{z}^{2}+H_{S}+H_{RS}$$

Replacing bare-rotor momentum **R**=**J**-**S** gives the following with a new constant spin energy *H*′*_RS_*.

(136) $$\begin{array}{c} H_{R,S(bod-fixed)}=A\;{\left(J_{x}-S_{x}\right)}^{2}+B\;{\left(J_{y}-S_{y}\right)}^{2}+C\;{\left(J_{z}-S_{z}\right)}^{2}+H_{RS}\\ =AJ_{x}^{2}+BJ_{y}^{2}+CJ_{z}^{2}-2AJ_{x}S_{x}-2BJ_{y}S_{y}-2CJ_{z}S_{z}+{H^{\prime }}_{RS} \end{array}$$

**Figure 31:**
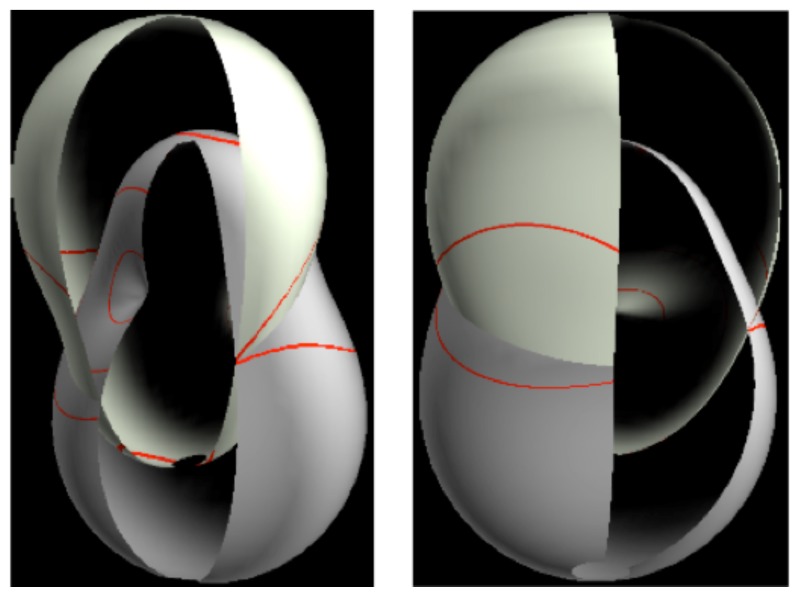
Views of classical rotor-gyro RES for spin +**S** (yellow) and −**S** (gray).

The simplest classical theory of the rotor-**R**-gyro-**S** momentum dynamics involves superimposed RES plots, one for +**S** and one for -**S** in Figure 31; A composite RES with *T* symmetry. If **J** and +**S** align (anti-align) then |**R**|=|**J**-**S**|, rotor energy Equation (135), and rotor-gyro relative velocity are minimized (maximized) (Thus, gyro-compass alignment with Earth rotation is seen to be relativistic quantum effect!).

A quantum theory of multiple RES involves mixing extreme cases |**J** ± **S**|. An elementary quantum gyro-spin is a two-state spin-1/2 with a 2-by-2 Hamiltonian matrix found by inserting quantum spin **S**=*σ/***2** matrices into Equation (136) to give Equation (137). Gyro-rotor dynamics involves REES obtained from eigensolutions of the following 2-by-2 matrix for each body-based **J**-vector Euler orientation (*β, γ* ).

(137) $$\begin{array}{l} H_{R,S(quantized)}=AJ_{x}^{2}+BJ_{y}^{2}+CJ_{z}^{2}-AJ_{x}\sigma_{x}-BJ_{y}\sigma_{y}-CJ_{z}\sigma_{z}+const.\\ =\left(\begin{array}{cc} {\text{RE}}_{\text{rotor}}-JC\;\text{cos }\beta & -AJ\;\text{cos }\gamma \;\text{sin }\beta -iBJ\;\text{sin }\gamma \;\text{sin }\beta \\ -AJ\;\text{cos }\gamma \;\text{sin }\beta +iBJ\;\text{sin }\gamma \;\text{sin }\beta & {\text{RE}}_{\text{rotor}}+JC\;\text{cos }\beta \end{array}\right)\\ \text{where}:{\text{RE}}_{\text{rotor}}=J^{2}(A{\text{cos}}^{2}\gamma {\text{sin}}^{2}\beta +B{\text{sin}}^{2}\gamma {\text{sin}}^{2}\beta +C{\text{cos}}^{2}\beta ) \end{array}$$

**Figure 32:**
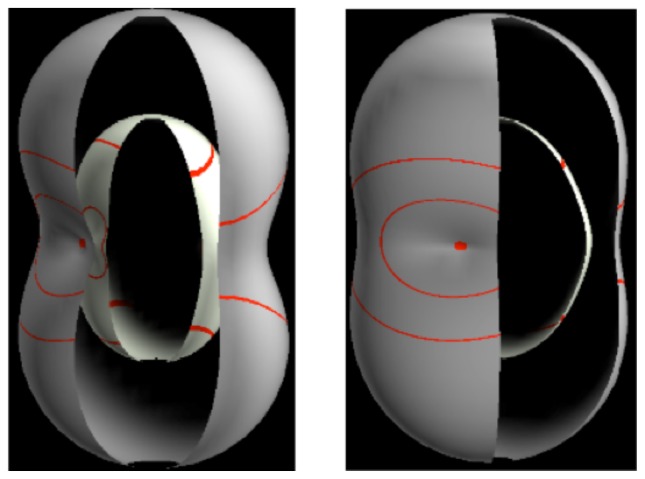
Same views of quantum REES for rotor with gyro spin operator **S**=*σ/***2**.

Eigensolutions of matrix form Equation (137) transform classical RES Figure 31 into quantum REES Figure 32 that has conical intersections or avoided crossing points replacing lines of classical surface intersections in the former Figure 31. Also, individual sheets of REES have **J**-inversion symmetry (or *T* symmetry) that individual RES lack. Where the RES of Figure 31 are well separated their shape is not so different from that of REES in Figure 32. Differences show up near the intersection lines where the two RES approach resonance. In this resonance region the REES is deformed extremely from rank-1 or rank-2 tensor shape of the separate RES, and there arises greater mixing of the extreme |±**S**| base-states.

### 9.2. REES of CF_4_ in ν_3_/2ν_4_ Dyad

**Figure 33:**
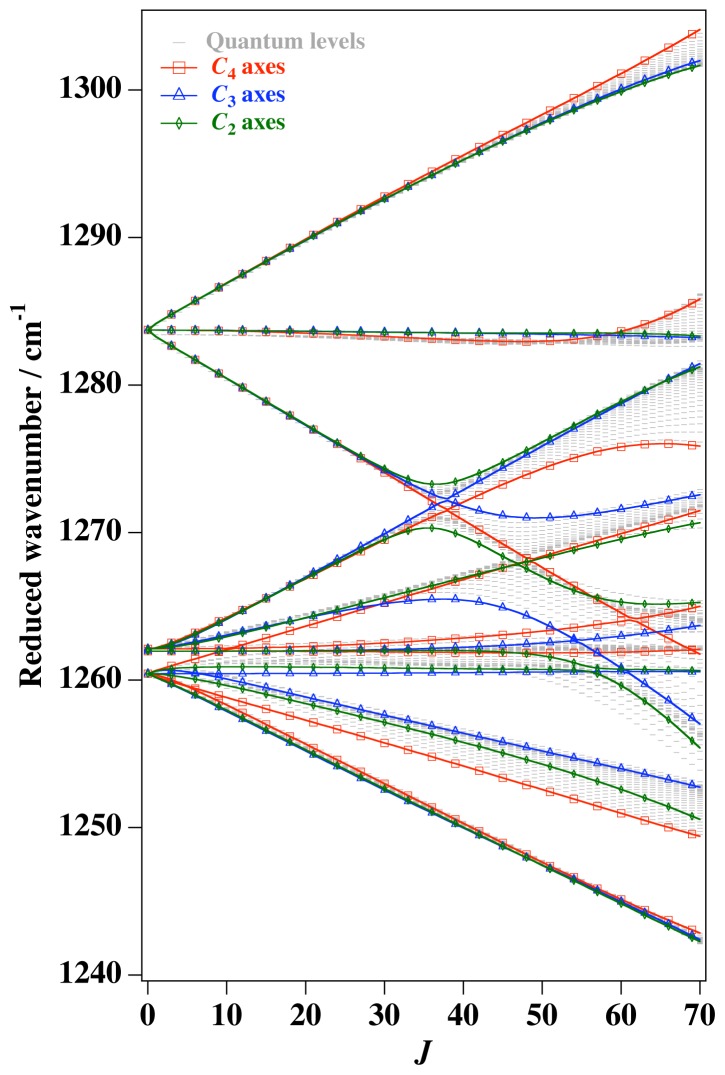
(After Boudon *et.al.* [2].) (*J*≤70) rotational levels of *ν*_3_*/*2*ν*_4_.

The first practical REES application includes 9-sheet displays of the *ν*_3_*/*2*ν*_4_ dyad of *CF*_4_ recently shown by Boudon *etal.* [2]. This large scale numerical analysis may be summarized by a revealing plot of dyad eigenlevels as a function of *J* = 0 to 70 in Figure 33. This includes colored lines representing the REES values for **J** located on *C*_4_ axes (shaded red), *C*_3_ axes (shaded blue), or *C*_2_ axes (shaded green).

Each of the symmetry axes may take turns as central loci for clusters of their type of local symmetry *C*_2_, *C*_3_, or *C*_4_, or else, they may sit on a REES separatrix or saddle point between two or more different types of clusters. A third option involves *C*_1_ clusters that have no rotation axis point but are likely to belong to vertical *xyz*-plane reflection symmetry *C_v_* = [1*, ρ_z_*] or diagonal-plane reflection symmetry *C_d_* = [1*, i*_3_]. These label clusters of 24 levels associated with 24 equivalent REES hills or valleys.

A final option involves true-*C*_1_ clusters with no local symmetry whatsoever and 48 REES hills or valleys. So far this extreme type has not been identified, but one may speculate that it may actually become most common at extremely high *J*.

**Figure 34:**
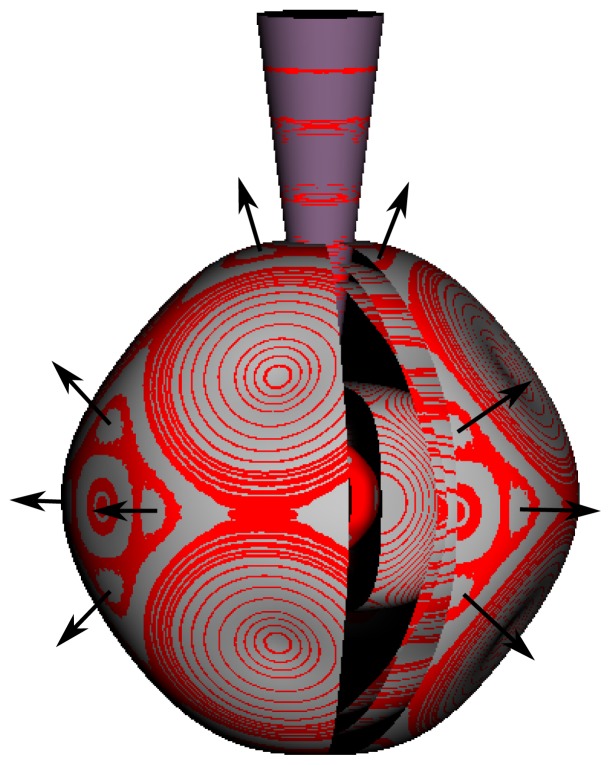
(After Boudon *et.al.* [2].) A rare (*J*=57)1_2_(*C*_2_)↑*O* structure on fifth REES.

A common ordering noted before on the left hand side of Figure 29 (pure *T*^[4]^) and in Figure 26 (16*μ* region of *SF*_6_) is (*C*_3_-valley→*C*_2_-saddle→*C*_4_-hill). It is present in the lowest REES band of Figure 33. An inverted version of the common ordering appears clearly in the 2*^nd^* band whose REES is cubic in Figure 34.

A cutaway view at *J* = 57 of the first five REES sheets shows glimpses of the first two REES deep inside of Figure 34. The second sheet has cubic topography similar to the inverted *T*^[4]^ RES on the right hand side of Figure 29 (pure (−)*T*^[4]^). However, the first and lowest REES for *J* = 57 is practically spherical with all 2*J* + 1=115 levels and clusters crushed in Figure 33 into near degeneracy!

After the first two REES sheets the cluster topography become more complicated with multiple conical intersections and avoided crossing points.

On the 5*^th^* sheet of the (*J* = 57)*ν*_3_*/*2*ν*_4_ REES are found examples of *C*_1_-local symmetry valleys as shown in Figure 34. (The upper four sheets are made invisible.) Each *C*_1_ loop occupies an area that is comparable to the minimum uncertainty (*J* = *K*)-cone shown on vertical *C*_4_ axis of the figure and a nascent 24-level cluster of type 1_2_(*C*_2_)↑*O* should be present in the level spectrum.

The symmetry details in this rovibrational spectra and the potential richness of quantum dynamics it represents should be quite evident from the few examples glimpsed here. We seem to be just scratching the surface of quantum systems of a great but potentially comprehensible complexity.

## 10. Summary and Conclusions

Semiclassical methods for visualizing and analyzing rovibrational dynamics of symmetric polyatomic molecules have been reviewed. This includes improved understanding of RES and REES phase spaces and development of more powerful symmetry methods to calculate tunneling dynamics of symmetric molecules that are highly resonant. A group-table-matrix analysis of intrinsic *vs.* extrinsic symmetry duality (The “Mock-Mach-Principles” Equation (95) and Equation (94) of wave relativity.) leads to generalizing character relations between group classes and irreducible representation into sub-character relations between sub-classes and induced representations Equation (131) and (132). These provide ortho-complete parameter relations (Tables 11, 12, 13, 14 and 15) for complex tunneling path lattices that determine molecular fine, superfine, and hyperfine spectra. The methods may be extensible to fluxional atomic and molecular systems.

## Figures and Tables

**Figure 35:**
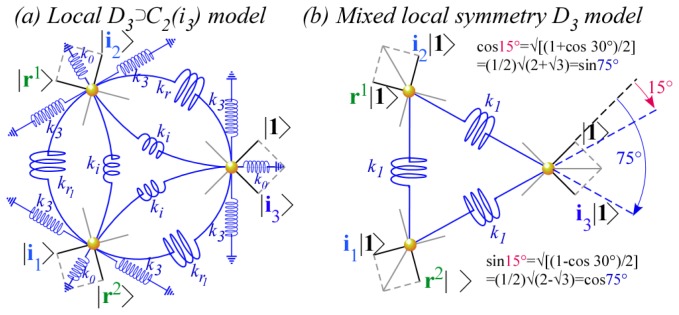
*X*_3_ spring models with local symmetry: (**a**) *D*_3_⊃*C*_2_(*i*_3_); (**b**) Mixed.

**Figure 36:**
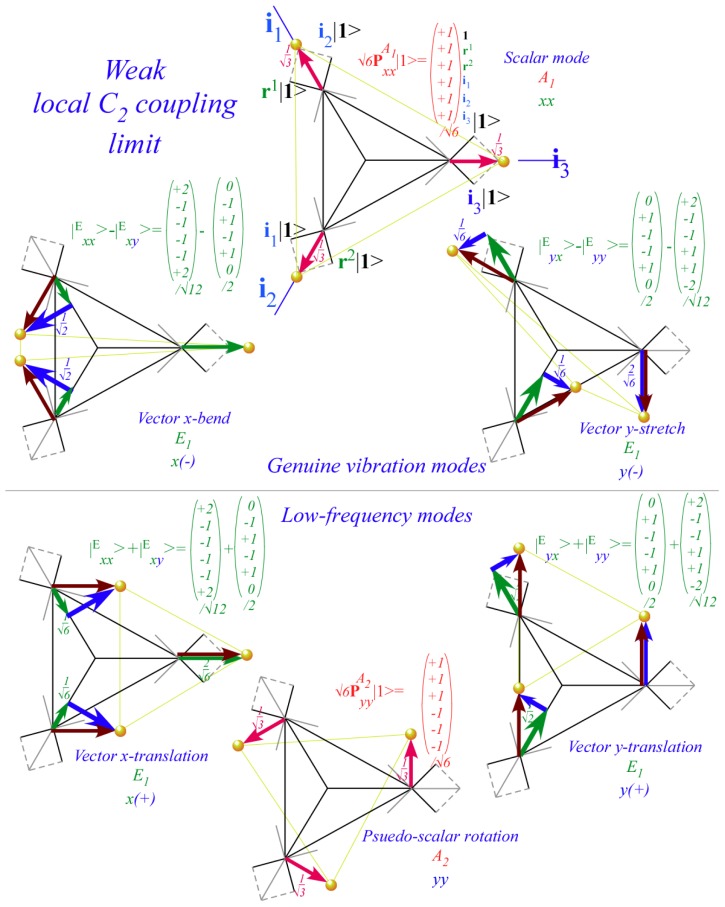
Mixed-local symmetry modes of direct-*k*_1_-coupled *X*_3_ model in Figure 35b.

**Figure 37:**
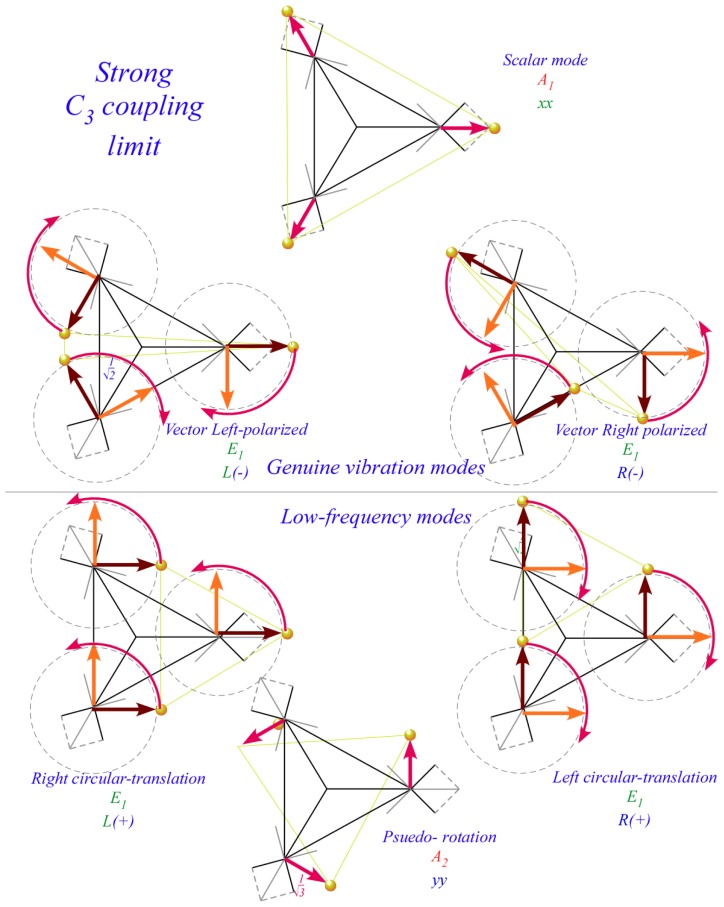
*D*_3_⊃*C*_3_-local symmetry modes of *X*_3_ molecule.

**Table 1:** Tabulated **v***_q_^k^* values for *J* = 1.

$\begin{array}{c} {\langle v_{2}^{2}\rangle }^{J=1}=\\ \left(\begin{array}{ccc} \cdot & \cdot & \cdot \\ \cdot & \cdot & \cdot \\ 1 & \cdot & \cdot \end{array}\right) \end{array}$	$\begin{array}{c} {\langle v_{1}^{2}\rangle }^{J=1}=\\ \left(\begin{array}{ccc} \cdot & \cdot & \cdot \\ 1 & \cdot & \cdot \\ \cdot & -1 & \cdot \end{array}\right)\begin{array}{c} \frac{1}{\sqrt{2}} \end{array} \end{array}$	$\begin{array}{c} {\langle v_{0}^{2}\rangle }^{J=1}=\\ \left(\begin{array}{ccc} 1 & \cdot & \cdot \\ \cdot & -2 & \cdot \\ \cdot & \cdot & 1 \end{array}\right)\begin{array}{c} \frac{1}{\sqrt{6}} \end{array} \end{array}$	$\begin{array}{c} {\langle v_{-1}^{2}\rangle }^{J=1}=\\ \left(\begin{array}{ccc} \cdot & -1 & \cdot \\ \cdot & \cdot & 1\\ \cdot & \cdot & \cdot \end{array}\right)\begin{array}{c} \frac{1}{2} \end{array} \end{array}$	$\begin{array}{c} {\langle v_{-2}^{2}\rangle }^{J=1}=\\ \left(\begin{array}{ccc} \cdot & \cdot & 1\\ \cdot & \cdot & \cdot \\ \cdot & \cdot & \cdot \end{array}\right) \end{array}$
	$\begin{array}{c} {\langle v_{1}^{1}\rangle }^{J=1}=\\ \left(\begin{array}{ccc} \cdot & \cdot & \cdot \\ 1 & \cdot & \cdot \\ \cdot & 1 & \cdot \end{array}\right)\begin{array}{c} \frac{1}{\sqrt{2}} \end{array} \end{array}$	$\begin{array}{c} {\langle v_{0}^{1}\rangle }^{J=1}=\\ \left(\begin{array}{ccc} 1 & \cdot & \cdot \\ \cdot & 0 & \cdot \\ \cdot & \cdot & -1 \end{array}\right)\begin{array}{c} \frac{1}{\sqrt{2}} \end{array} \end{array}$	$\begin{array}{c} {\langle v_{-1}^{1}\rangle }^{J=1}=\\ \left(\begin{array}{ccc} \cdot & -1 & \cdot \\ \cdot & \cdot & -1\\ \cdot & \cdot & \cdot \end{array}\right)\begin{array}{c} \frac{1}{\sqrt{2}} \end{array} \end{array}$	
		$\begin{array}{c} {\langle v_{0}^{0}\rangle }^{J=1}=\\ \left(\begin{array}{ccc} 1 & \cdot & \cdot \\ \cdot & 1 & \cdot \\ \cdot & \cdot & 1 \end{array}\right)\begin{array}{c} \frac{1}{\sqrt{3}} \end{array} \end{array}$		
		$\begin{array}{c} {\langle v_{q=-2\ldots 2}^{2}\rangle }^{J=1}=\\ \left(\begin{array}{ccc} 1 & -1 & 1\\ 1 & -2 & 1\\ 1 & -1 & 1 \end{array}\right)|\begin{array}{c} 1\\ \frac{1}{\sqrt{2}}\\ \frac{1}{\sqrt{6}} \end{array} \end{array}$		
		$\begin{array}{c} {\langle v_{q=-1\ldots 1}^{1}\rangle }^{J=1}=\\ \left(\begin{array}{ccc} 1 & -1 & \cdot \\ 1 & 0 & -1\\ \cdot & 1 & -1 \end{array}\right)|\begin{array}{c} \cdot \\ \frac{1}{\sqrt{3}}\\ \frac{1}{\sqrt{2}} \end{array} \end{array}$		
		$\begin{array}{c} {\langle v_{0}^{0}\rangle }^{J=1}=\\ \left(\begin{array}{ccc} 1 & \cdot & \cdot \\ \cdot & 1 & \cdot \\ \cdot & \cdot & 1 \end{array}\right)|\begin{array}{c} \cdot \\ \cdot \\ \frac{1}{\sqrt{3}} \end{array} \end{array}$		

**Table 2:** Unit tensor representations.

$\begin{array}{c} {\langle v_{q=-1\ldots 1}^{1}\rangle }^{J=1}=\\ \begin{array}{ccc} 1 & 1 & \cdot \\ 1 & 0 & -1\\ \cdot & 1 & -1 \end{array}|\begin{array}{c} \cdot \\ \frac{1}{\sqrt{3}}\\ \frac{1}{\sqrt{2}} \end{array} \end{array}$	$\begin{array}{c} {\langle v_{q=-1\ldots 1}^{1}\rangle }^{J=2}=\\ \begin{array}{ccccc} 2 & -\sqrt{2} & \cdot & \cdot & \cdot \\ \sqrt{2} & 1 & -\sqrt{3} & \cdot & \cdot \\ \cdot & \sqrt{3} & 0 & -\sqrt{3} & \cdot \\ \cdot & \cdot & \sqrt{3} & -1 & -\sqrt{2}\\ \cdot & \cdot & \cdot & \sqrt{2} & -2 \end{array}|\begin{array}{c} \cdot \\ \cdot \\ \cdot \\ \frac{1}{\sqrt{10}}\\ \frac{1}{\sqrt{10}} \end{array} \end{array}$	$\begin{array}{c} {\langle v_{q=-1\ldots 1}^{1}\rangle }^{J=3}=\\ \begin{array}{ccccccc} 3 & -\sqrt{3} & \cdot & \cdot & \cdot & \cdot & \cdot \\ \sqrt{3} & 2 & -\sqrt{5} & \cdot & \cdot & \cdot & \cdot \\ \cdot & \sqrt{5} & 1 & -\sqrt{6} & \cdot & \cdot & \cdot \\ \cdot & \cdot & \cdot & \sqrt{6} & -1 & -\sqrt{5} & \cdot \\ \cdot & \cdot & \cdot & \cdot & \sqrt{5} & -2 & -\sqrt{3}\\ \cdot & \cdot & \cdot & \cdot & \cdot & \sqrt{3} & -3 \end{array}|\begin{array}{c} \cdot \\ \cdot \\ \cdot \\ \cdot \\ \cdot \\ \frac{1}{\sqrt{28}}\\ \frac{1}{\sqrt{28}} \end{array} \end{array}$
$\begin{array}{c} {\langle v_{q=-2\ldots 2}^{2}\rangle }^{J=1}=\\ \begin{array}{ccc} 1 & -1 & 1\\ 1 & -2 & 1\\ 1 & -1 & 1 \end{array}|\begin{array}{c} 1\\ \frac{1}{\sqrt{2}}\\ \frac{1}{\sqrt{6}} \end{array} \end{array}$	$\begin{array}{c} {\langle v_{q=-2\ldots 2}^{2}\rangle }^{J=2}=\\ \begin{array}{ccccc} 2 & -\sqrt{6} & \sqrt{2} & \cdot & \cdot \\ \sqrt{6} & -1 & -1 & \sqrt{3} & \cdot \\ \sqrt{2} & 1 & -2 & 1 & \sqrt{6}\\ \cdot & \cdot & \sqrt{2} & -\sqrt{6} & 2 \end{array}|\begin{array}{c} \cdot \\ \cdot \\ \frac{1}{\sqrt{7}}\\ \frac{1}{\sqrt{14}}\\ \frac{1}{\sqrt{14}} \end{array} \end{array}$	$\begin{array}{c} {\langle v_{q=-2\ldots 2}^{2}\rangle }^{J=3}=\\ \begin{array}{ccccccc} 5 & -5 & \sqrt{5} & \cdot & \cdot & \cdot & \cdot \\ 5 & 0 & -\sqrt{15} & \sqrt{10} & \cdot & \cdot & \cdot \\ \sqrt{5} & \sqrt{15} & -3 & -\sqrt{2} & \sqrt{12} & \cdot & \cdot \\ . & \sqrt{10} & \sqrt{2} & -4 & \sqrt{2} & \sqrt{10} & .\\ . & . & \sqrt{12} & -\sqrt{2} & -3 & \sqrt{15} & \sqrt{5}\\ \cdot & \cdot & \cdot & \sqrt{10} & -\sqrt{15} & 0 & 5\\ \cdot & \cdot & \cdot & \cdot & \sqrt{5} & -5 & 5 \end{array}|\begin{array}{c} \cdot \\ \cdot \\ \cdot \\ \cdot \\ \frac{1}{\sqrt{42}}\\ \frac{1}{\sqrt{84}}\\ \frac{1}{\sqrt{84}} \end{array} \end{array}$
	$\begin{array}{c} {\langle v_{q=-3\ldots 3}^{3}\rangle }^{J=2}=\\ \begin{array}{ccccc} 1 & \sqrt{3} & 1 & -1 & \cdot \\ \sqrt{3} & -2 & \sqrt{2} & 0 & -1\\ 1 & -\sqrt{2} & 0 & \sqrt{2} & -1\\ 1 & 0 & -\sqrt{2} & 2 & -\sqrt{3}\\ \cdot & 1 & -1 & \sqrt{3} & -1 \end{array}|\begin{array}{c} \cdot \\ \frac{1}{\sqrt{2}}\\ \frac{1}{\sqrt{2}}\\ \frac{1}{\sqrt{10}}\\ \frac{1}{\sqrt{10}} \end{array} \end{array}$	$\begin{array}{c} {\langle v_{q=-3\ldots 3}^{3}\rangle }^{J=3}=\\ \begin{array}{ccccccc} 1 & -\sqrt{2} & \sqrt{2} & -1 & \cdot & \cdot & \cdot \\ \sqrt{2} & -1 & 0 & 1 & -\sqrt{2} & \cdot & \cdot \\ 1 & 1 & -1 & 0 & 1 & -1 & -1\\ \cdot & \sqrt{2} & 0 & -1 & 1 & 0 & -\sqrt{2}\\ \cdot & \cdot & \sqrt{2} & -1 & 0 & 1 & -\sqrt{2}\\ \cdot & \cdot & \cdot & 1 & -\sqrt{2} & \sqrt{2} & -1 \end{array}|\begin{array}{c} \cdot \\ \cdot \\ \cdot \\ \frac{1}{\sqrt{6}}\\ \frac{1}{\sqrt{6}}\\ \frac{1}{\sqrt{6}}\\ \frac{1}{\sqrt{6}} \end{array} \end{array}$
	$\begin{array}{c} {\langle v_{q=-4\ldots 4}^{4}\rangle }^{J=2}=\\ \begin{array}{ccccc} 1 & -1 & \sqrt{3} & -1 & 1\\ 1 & -4 & \sqrt{6} & -\sqrt{8} & 1\\ \sqrt{3} & -\sqrt{6} & 6 & -\sqrt{6} & \sqrt{3}\\ 1 & -\sqrt{8} & \sqrt{6} & -4 & 1\\ 1 & -1 & \sqrt{3} & -1 & 1 \end{array}|\begin{array}{c} 1\\ \frac{1}{\sqrt{2}}\\ \frac{1}{\sqrt{14}}\\ \frac{1}{\sqrt{14}}\\ \frac{1}{\sqrt{70}} \end{array} \end{array}$	$\begin{array}{c} {\langle v_{q=-4\ldots 4}^{4}\rangle }^{J=3}=\\ \begin{array}{ccccccc} 3 & -\sqrt{30} & \sqrt{54} & -3 & \sqrt{3} & \cdot & \cdot \\ \sqrt{30} & -7 & \sqrt{32} & -\sqrt{3} & -\sqrt{2} & \sqrt{5} & \cdot \\ \sqrt{54} & -\sqrt{32} & 1 & \sqrt{15} & -\sqrt{40} & \sqrt{2} & \sqrt{3}\\ 3 & -\sqrt{3} & -\sqrt{15} & 6 & -\sqrt{15} & -\sqrt{3} & 3\\ \sqrt{3} & \sqrt{2} & -\sqrt{40} & \sqrt{15} & 1 & -\sqrt{32} & \sqrt{54}\\ \sqrt{5} & -\sqrt{2} & -\sqrt{3} & \sqrt{32} & -7 & \sqrt{30} & \\ \cdot & \cdot & \sqrt{3} & -3 & \sqrt{54} & -\sqrt{30} & 3 \end{array}|\begin{array}{c} \cdot \\ \frac{1}{\sqrt{2}}\\ \frac{1}{\sqrt{2}}\\ \frac{1}{\sqrt{6}}\\ \frac{1}{\sqrt{6}}\\ \frac{1}{\sqrt{84}}\\ \frac{1}{\sqrt{84}} \end{array} \end{array}$
		$\begin{array}{c} {\langle v_{q=-5\ldots 5}^{5}\rangle }^{J=3}=\\ \begin{array}{ccccccc} 1 & -\sqrt{5} & 1 & -\sqrt{2} & 1 & -1 & \cdot \\ \sqrt{5} & -4 & \sqrt{27} & -\sqrt{2} & 1 & 0 & -1\\ 1 & -\sqrt{27} & 5 & -\sqrt{10} & 0 & 1 & -1\\ \sqrt{2} & -\sqrt{2} & \sqrt{10} & 0 & -\sqrt{10} & \sqrt{2} & -\sqrt{2}\\ 1 & -1 & 0 & \sqrt{10} & -5 & \sqrt{27} & -1\\ 1 & 0 & -1 & \sqrt{2} & -\sqrt{27} & 4 & -\sqrt{5}\\ \cdot & 1 & -1 & \sqrt{2} & -1 & \sqrt{5} & -1 \end{array}|\begin{array}{c} \cdot \\ \frac{1}{\sqrt{2}}\\ \frac{1}{\sqrt{2}}\\ \frac{1}{\sqrt{6}}\\ \frac{1}{\sqrt{6}}\\ \frac{1}{\sqrt{84}}\\ \frac{1}{\sqrt{84}} \end{array} \end{array}$
		$\begin{array}{c} {\langle v_{q=-6\ldots 6}^{6}\rangle }^{J=3}=\\ \begin{array}{ccccccc} 1 & -\sqrt{2} & 1 & -\sqrt{2} & \sqrt{5} & -1 & 1\\ \sqrt{2} & -6 & \sqrt{30} & -\sqrt{8} & 3 & -\sqrt{12} & 1\\ 1 & -\sqrt{30} & 15 & -10 & \sqrt{15} & -3 & \sqrt{5}\\ \sqrt{2} & -\sqrt{8} & 10 & -20 & 10 & -\sqrt{8} & \sqrt{2}\\ \sqrt{5} & -3 & \sqrt{15} & -10 & 15 & -\sqrt{30} & 1\\ 1 & -\sqrt{12} & 3 & -\sqrt{8} & \sqrt{30} & -6 & \sqrt{2}\\ 1 & -1 & \sqrt{5} & -\sqrt{2} & 1 & -\sqrt{2} & 1 \end{array}|\begin{array}{c} 1\\ \frac{1}{\sqrt{2}}\\ \frac{1}{\sqrt{22}}\\ \frac{1}{\sqrt{22}}\\ \frac{1}{\sqrt{33}}\\ \frac{1}{\sqrt{264}}\\ \frac{1}{\sqrt{924}} \end{array} \end{array}$

**Table 3:** Tabulated **v***_q_^k^* values and relation to quaternions. (**a**) Tabulated **v***_q_^k^* values for *J* = 1*=*2; (**b**) Simple Conversion from **v** to *σ*; (**c**) Conventional quaternion-spinor relations.

(a) Tabulated **v***_q_^k^* values for J=1/2			

$\begin{array}{c} {\langle v_{-1}^{1}\rangle }^{J=1/2}=\\ \left(\begin{array}{cc} \cdot & \cdot \\ -1 & \cdot \end{array}\right) \end{array}$	$\begin{array}{c} {\langle v_{0}^{1}\rangle }^{J=1/2}=\\ -\left(\begin{array}{cc} 1 & \cdot \\ \cdot & -1 \end{array}\right)\frac{1}{\sqrt{2}} \end{array}$	$\begin{array}{c} {\langle v_{1}^{1}\rangle }^{J=1/2}=\\ \left(\begin{array}{cc} \cdot & 1\\ \cdot & \cdot \end{array}\right) \end{array}$	$\begin{array}{c} {\langle v_{-1\ldots 1}^{1}\rangle }^{J=1/2}=\\ \left(\begin{array}{cc} -1 & 1\\ -1 & 1 \end{array}\right)|\begin{array}{c} 1\\ \frac{1}{\sqrt{2}} \end{array} \end{array}$

	$\begin{array}{c} {\langle v_{0}^{0}\rangle }^{J=1/2}=\\ \left(\begin{array}{cc} -1 & \cdot \\ \cdot & -1 \end{array}\right)\frac{1}{\sqrt{2}} \end{array}$		$\begin{array}{c} {\langle v_{0}^{0}\rangle }^{J=1/2}=\\ -\left(\begin{array}{cc} 1 & \cdot \\ \cdot & 1 \end{array}\right)\begin{array}{c} \cdot \\ \frac{1}{\sqrt{2}} \end{array} \end{array}$
			
(b) Simple Conversion from **v** to *σ*			

**V**_−1_^1^ = −*σ*_−_	$v_{0}^{1}=-\frac{1}{\sqrt{2}}\sigma_{z}$	**V**_+1_^1^ = +*σ*_+_	**V**_0_^0^ = +*σ*_0_

$\begin{array}{c} \sigma_{x}=\sigma_{+}+\sigma_{-}\\ =\left(\begin{array}{cc} \cdot & 1\\ 1 & \cdot \end{array}\right) \end{array}$	$\begin{array}{c} \sigma_{z}=-\sqrt{2}v_{0}^{1}\\ =\left(\begin{array}{cc} +1 & \cdot \\ \cdot & -1 \end{array}\right) \end{array}$	$\begin{array}{c} \sigma_{y}=-i\sigma_{+}+i\sigma_{-}\\ =\left(\begin{array}{cc} \cdot & -i\\ i & \cdot \end{array}\right) \end{array}$	$\begin{array}{c} \sigma_{0}=-\sqrt{2}v_{0}^{0}\\ =\left(\begin{array}{cc} 1 & 0\\ 0 & 1 \end{array}\right) \end{array}$

(c) Conventional quaternion-spinor relations			

$\begin{array}{c} i=i\sigma_{x}\\ =\left(\begin{array}{cc} 0 & i\\ i & 0 \end{array}\right) \end{array}$	$\begin{array}{c} k=i\sigma_{z}\\ =\left(\begin{array}{cc} +i & 0\\ 0 & -i \end{array}\right) \end{array}$	$\begin{array}{c} j=i\sigma_{y}\\ =\left(\begin{array}{cc} 0 & 1\\ -1 & 0 \end{array}\right) \end{array}$	$\begin{array}{c} 1=\sigma_{0}\\ =\left(\begin{array}{cc} 1 & 0\\ 0 & 1 \end{array}\right) \end{array}$

**Table 4:** Forming 〈**v**_0_*^k^*〉 from powers of *J* and *m*.

${\langle v_{0}^{k}\rangle }_{m}^{J}=\langle \begin{array}{c} J\\ m \end{array}|v_{0}^{k}|\begin{array}{c} J\\ m \end{array}\rangle ={\left(-1\right)}^{J-m}\sqrt{\left[k\right]}\left(\begin{array}{ccc} k & J & J\\ 0 & m & -m \end{array}\right)={\left(-1\right)}^{k}\sqrt{\frac{\left[k\right]}{\left[J\right]}}C_{0mm}^{kJJ}$					
〈**v**_0_^0^〉*_m_^J^* =	$\frac{1}{\sqrt{2J+1}}$	[1]			
〈**v**_0_^1^〉*_m_^J^* =	$\frac{2\sqrt{3}}{\sqrt{2J+2:0}}$	[	*m*]		
〈**v**_0_^2^〉*_m_^J^* =	$\frac{2^{2}\sqrt{5}}{\sqrt{2J+3:-1}}$	$\left[-\frac{1}{2}J(J+1\right)$		$+\frac{3}{2}m^{2}]$	
〈**v**_0_^3^〉*_m_^J^* =	$\frac{2^{3}\sqrt{7}}{\sqrt{2J+4:-2}}$	[	$-\frac{3}{2}(J(J+1)-\frac{2}{3})m$		$+\frac{5}{2}m^{3}]$

**Table 5:** Forming 〈**v**_0_*^k^*〉 from powers of *J* and *m*, expanded.

*k*	*m*^0^	*m*^1^	*m*^2^	*m*^3^	*m*^4^	*m*^5^	*m*^6^	*m*^7^
0	1							
1		1						
2	$\frac{-1}{2}J(J+1)$		$\frac{3}{2}$					
3		$\frac{-3}{2}(J(J+1)-\frac{2}{3})$		$\frac{5}{2}$				
4	$\frac{3}{8}(J+2:-1)$		$\frac{-30}{8}(J(J+1)-\frac{5}{6})$		$\frac{35}{8}$			
5		$\frac{15}{8}\left((J+2:-1)-\frac{4}{3}J(J+1)-\frac{4}{5}\right)$		$\frac{-70}{8}\left(J(J+1)-\frac{3}{2}\right)$		$\frac{63}{8}$		
6	$\frac{-5}{16}(J+3:-2)$		$\frac{105}{16}\left((J+2:-1)-3J(J+1)+\frac{14}{5}\right)$		$\frac{-315}{16}\left(J(J+1)-\frac{7}{3}\right)$		$\frac{231}{16}$	
7		$\frac{-35}{16}\left((J+3:-2)-3(J+2:-1)+\frac{36}{5}J(J+1)-\frac{36}{7}\right)$		$\frac{315}{16}\left((J+2:-1)-5J(J+1)+\frac{61}{9}\right)$		$\frac{-693}{16}\left(J(J+1)-\frac{10}{3}\right)$		$\frac{420}{16}$
8	35(*J* + 4 : −3)		$\frac{-1260}{128}\left((J+3:-2)-\frac{13}{2}(J+2:-1)+\frac{332}{15}J(J+1)-\frac{761}{35}\right)$		$\frac{6930}{128}\left((J+2:-1)-\frac{22}{3}J(J+1)-\frac{1871}{1386}\right)$		$\frac{-12012}{128}\left(J(J+1)+\frac{9}{2}\right)$	$\frac{6435}{128}$

**Table 6:** Orthorhombic 4-group *D*_2_ = *C*_2_ × *C*_2_ character table construction.

								*D*_2_ = *C*_2_(*x*) × *C*_2_(*y*)	**1**	**R***_x_*	**R***_y_*	**R***_z_*
								
*C*_2_(*x*)	**1**	**R***_x_*		*C*_2_(*y*)	**1**	**R***_y_*		*A*_1_ = (0_2_0_2_)*_xy_*	**1**	**1**	**1**	**1**
								
*A* = (0_2_)*_x_*	1	1	×	1 = (0_2_)*_y_*	**1**	1	=	*A*_2_ = (0_2_1_2_)*_xy_*	**1**	**−1**	**1**	**−1**
								
*B* = (1_2_)*_x_*	1	−1		2 = (1_2_)*_y_*	**1**	−1		*B*_1_ = (1_2_0_2_)*_xy_*	**1**	**1**	**−1**	**−1**
								
								*B*_2_ = (1_2_1_2_)*_xy_*	**1**	**−1**	**−1**	**1**
								

**Table 7:** Group character tables for cyclic groups of symmetry order N. (**a**) N = 2; (**b**) N = 3: *ε* = *e*^2^*^π/^*^3^; (**c**) N = 4.

(a)		
*C*_2_	1	R*_x_*
(0_2_)	1	1
(1_2_)	1	−1

(b)			
*C*_3_	1	R^1^	R^2^
(0_3_)	1	1	1
(1_3_)	1	ε^*^	*ε*
(2_3_)	1	*ε*	ε^*^

(c)				
*C*_4_	1	R^1^	R^2^	R^3^
(0_4_)	1	1	1	1
(1_4_)	1	−*i*	−1	*i*
(2_4_)	1	−1	1	−1
(3)_4_	1	*i*	−1	−*i*

**Table 8:** Symmetry correlation table between species of *D*_2_ and its axial subgroups. (**a**) *C*_2_(*x*) subgroup; (**b**) *C*_2_(*y*) subgroup; (**c**) *C*_2_(*z*) subgroup.

(a)		
*D*_2_ ⊃ *C*_2_(*x*)	(0_2_)*_x_*	(1_2_)*_x_*
*A*_1_	1	·
*A*_2_	·	1
*B*_1_	1	·
*B*_2_	·	1

(b)		
*D*_2_ ⊃ *C*_2_(*y*)	(0_2_)*_y_*	(1_2_)*_y_*
*A*_1_	1	·
*A*_2_	1	·
*B*_1_	·	1
*B*_2_	·	1

(c)		
*D*_2_ ⊃ *C*_2_(*z*)	(0_2_)*_z_*	(1_2_)*_z_*
*A*_1_	1	·
*A*_2_	·	1
*B*_1_	·	1
*B*_2_	1	·

**Table 9:** Correlation tables between octahedral symmetric, *O* and various cyclic subgroups.

(a)				
*O* ⊃ *C*_4_	0_4_	1_4_	2_4_	3_4_
*A*_1_ ↓ *C*_4_	1	·	·	·
*A*_2_ ↓ *C*_4_	·	·	1	·
*E* ↓ *C*_4_	1	·	1	·
*T*_1_ ↓ *C*_4_	1	1	·	1
*T*_2_ ↓ *C*_4_	·	1	1	1

(b)			
*O* ⊃ *C*_3_	0_3_	1_3_	2_3_
*A*_1_ ↓ *C*_3_	1	·	·
*A*_2_ ↓ *C*_3_	1	·	·
*E* ↓ *C*_3_	·	1	1
*T*_1_ ↓ *C*_3_	1	1	1
*T*_2_ ↓ *C*_3_	1	1	1

(c)		
*O* ⊃ *C*_2_(**i**_1_)	0_2_	1_2_
*A*_1_ ↓ *C*_2_	1	·
*A*_2_ ↓ *C*_2_	·	1
*E* ↓ *C*_2_	1	1
*T*_1_ ↓ *C*_2_	1	2
*T*_2_ ↓ *C*_2_	2	1

(d)		
*O* ⊃ *C*_2_(*ρ_z_*)	0_2_	1_2_
*A*_1_ ↓ *C*_2_	1	·
*A*_2_ ↓ *C*_2_	1	·
*E* ↓ *C*_2_	2	·
*T*_1_ ↓ *C*_2_	1	2
*T*_2_ ↓ *C*_2_	1	2

**Table 10:** RES plots exploring the 2D parameter space.

	*θ* = 0	*θ* = *π/*4	*θ* = *π/*2	*θ* = 3*π/*4	*θ* = *π*
*φ* = 0	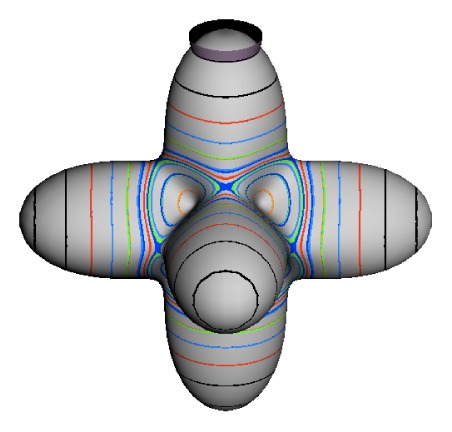	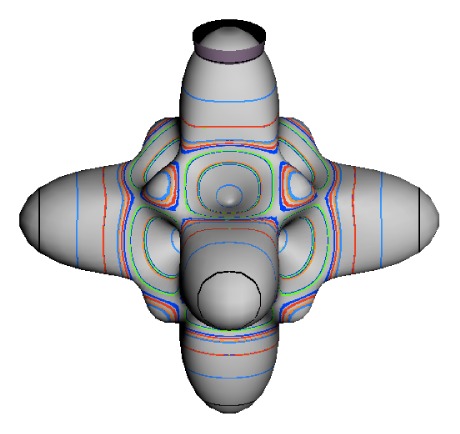	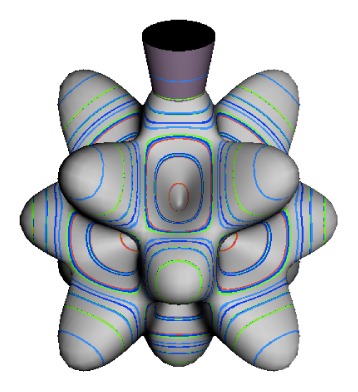	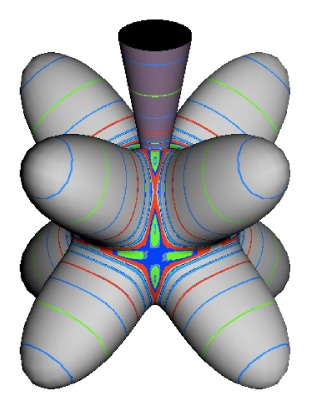	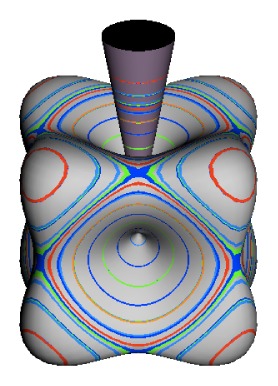
_$\varphi =\frac{\pi }{4}$_	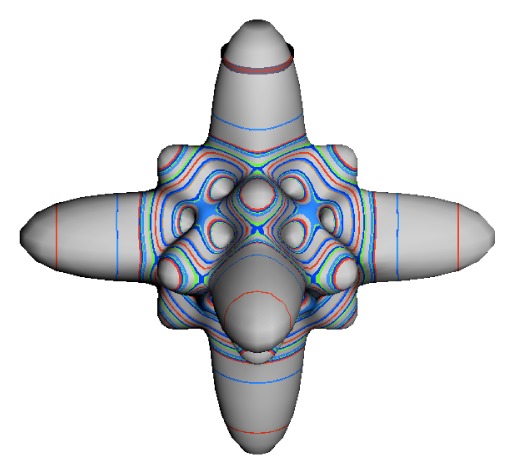	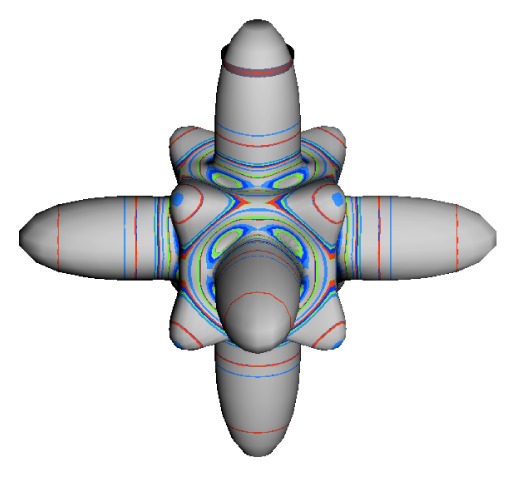	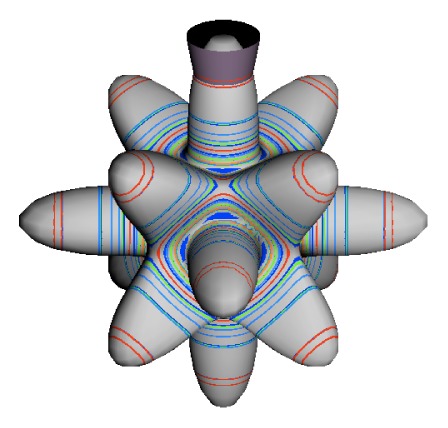	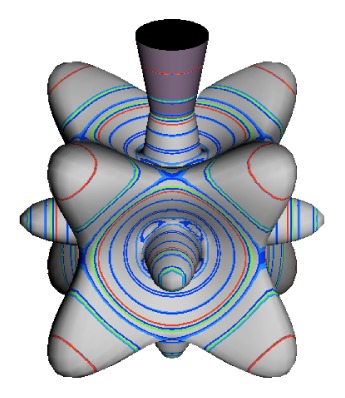	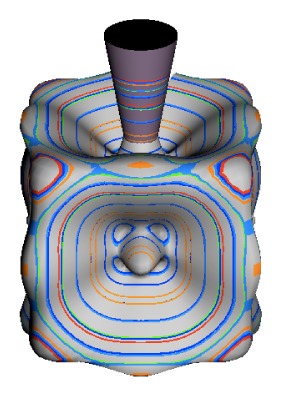
_$\varphi =\frac{\pi }{2}$_	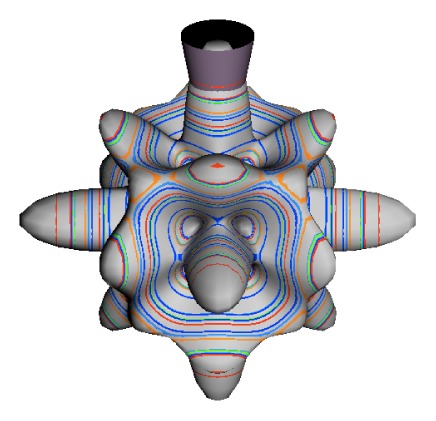	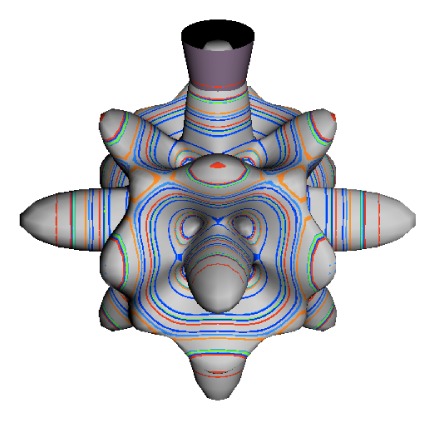	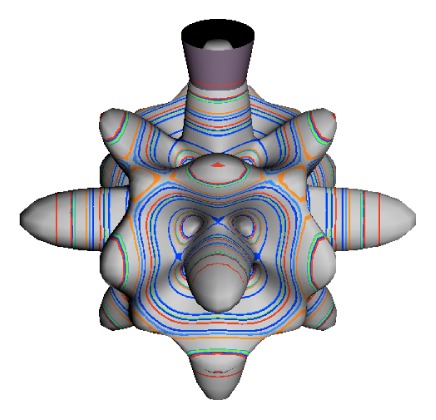	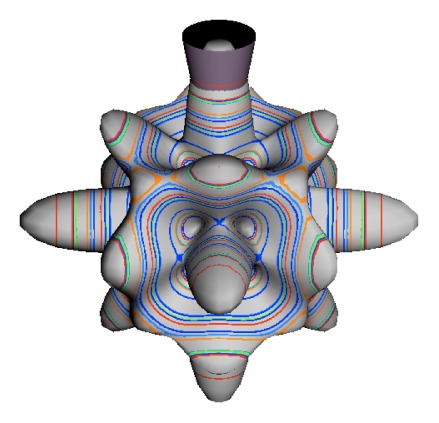	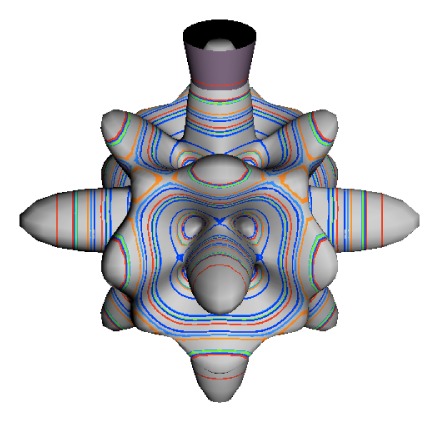
_$\varphi =\frac{3\pi }{4}$_	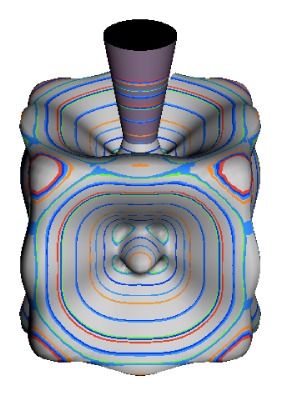	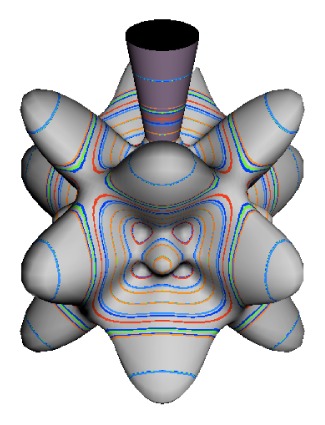	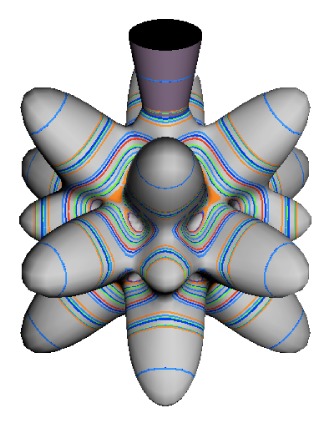	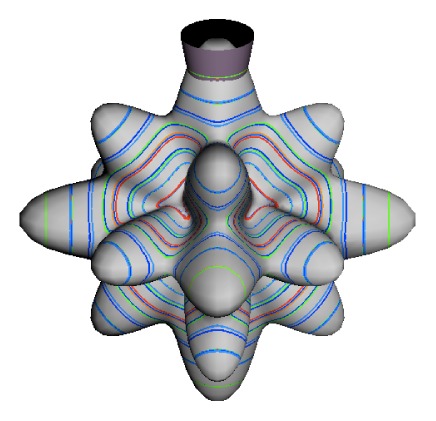	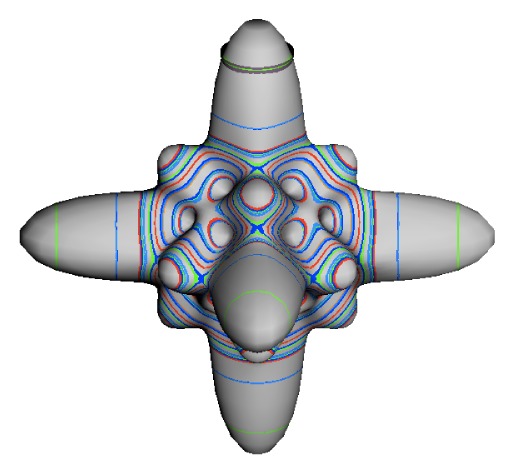
*φ* = *π*	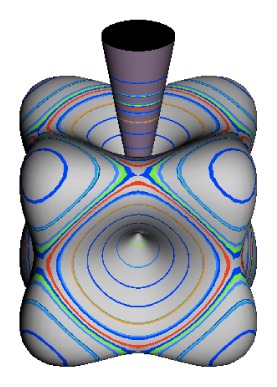	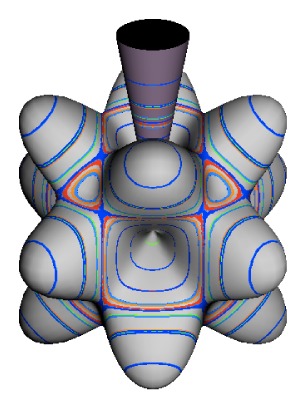	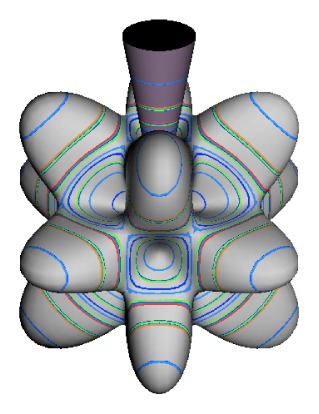	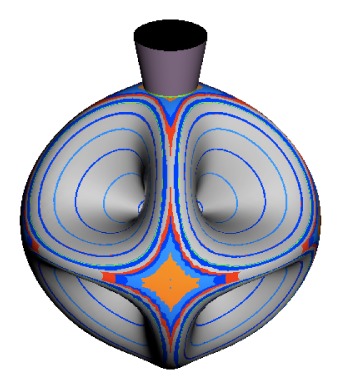	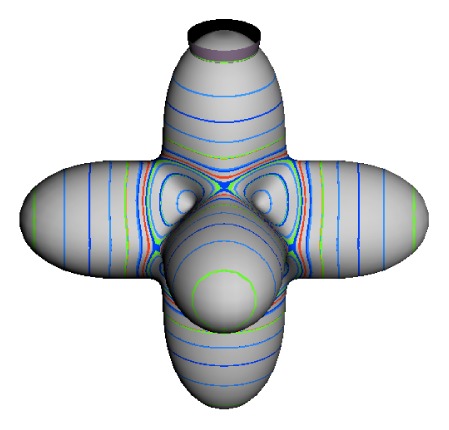

**Table 11:** Splittings of *O* ⊃ *C*_4_ given sub-class structure.

*O* ⊃ *C*_4_	0°	*r_n_*120°	*ρ_n_*180°	*R_n_*90°	*i_n_*180°

0_4_	·	*r*_I_ = Re *r*_1234_ *m*_I_ = Im *r*_1234_	·	*R_z_* = Re*R_z_* *I_z_* = Im*R_z_*	*i*_I_ = *i*_1256_ *i*_II_ = *i*_34_

*ε*_0_4__*^A^*^_1_^ =	*g*_0_	+8*r*_I_	+2*ρ_xy_* + *ρ_z_*	+4*R_xy_* + 2*R_z_*	+4*i*_I_ + 2*i*_II_
*ε*_0_4__*^T^*^_1_^	*g*_0_	0	−2*ρ_xy_* + *ρ_z_*	+2*R_z_*	−2*i*_II_
*ε*_0_4__*^E^*	*g*_0_	−2*r*_I_	+2*ρ_xy_* + *ρ_z_*	−2*R_xy_* − *R_z_*	−2*i*_I_ + 2*i*_II_

1_4_	·	·	·	·	·

*ε*_1_4__*^T^*^_2_^	*g*_0_	+2*m*_I_	−*ρ_z_*	−*R_xy_* − 2*I_z_*	+2*i*_I_
*ε*_1_4__*^T^*^_1_^	*g*_0_	−2*m*_I_	−*ρ_z_*	+*R_xy_* − 2*I_z_*	−2*i*_I_

2_4_	·	·	·	·	·

*ε*_2_4__*^E^*	*g*_0_	−2*r*_I_	+2*ρ_xy_* + *ρ_z_*	+2*R_xy_* − *R_z_*	+2*i*_I_ − 2*i*_II_
*ε*_2_4__*^T^*^_2_^	*g*_0_	0	−2*ρ_xy_* + *ρ_z_*	−2*R_z_*	+2*i*_II_
*ε*_2_4__*^A^*^_2_^	*g*_0_	+8*r*_I_	+2*ρ_xy_* + *ρ_z_*	−4*R_xy_* − 2*R_z_*	−4*i*_I_ − 2*i*_II_

3_4_	·	·	·	·	·

*ε*_3_4__*^T^*^_2_^	*g*_0_	−2*m*_I_	−*ρ_z_*	−*R_xy_* + 2*I_z_*	+2*i*_I_
*ε*_3_4__*^T^*^_1_^	*g*_0_	+2*m*_I_	−*ρ_z_*	+*R_xy_* + 2*I_z_*	−2*i*_I_

**Table 12:** Splittings of *O* ⊃ *C*_3_ given sub-class structure.

*O* ⊃ *C*_3_	0°	*r_n_*120°	*ρ_n_*180°	*R_n_*90°	*i_n_*180°

0_3_	·	*r*_I_ = *Re*(*r*_1_) *i*_I_ = *Im*(*r*_1_) *r*_II_ = *Re*(*r*_234_) *i*_I_ = *Im*(*r*_234_)	*ρ* = *ρ_xyz_*	*R_n_* = *Re*(*R_xyz_*) *I_n_* = *Im*(*R_xyz_*)	*i*_I_ = *i*_136_ *i*_II_ = *i*_245_

*ɛ*_0_3__*^A^*^_1_^	*g*_0_	2*r*_I_ + 6*r*_II_	3*ρ*	6*R_n_*	3*i*_I_ + 3*i*_I_
*ɛ*_0_3__*^A^*^_2_^	*g*_0_	2*r*_I_ + 6*r*_II_	3*ρ*	−6*R_n_*	−3*i*_I_ − 3*i*_II_
*ɛ*_0_3__*^T^*^_1_^	*g*_0_	2*r*_I_ − 2*r*_II_	−*ρ*	−2Rn	*i*_I_ − 3*i*_II_
*ɛ*_0_3__*^T^*^_2_^	*g*_0_	2*r*_I_ − 2*r*_II_	−*ρ*	−2*R_n_*	−*i*_I_ + 3*i*_II_

1_3_					

*ɛ*_1_3__*^E^*	*g*_0_	$-r_{\text{I}}+\sqrt{3}i_{\text{I}}-3r_{\text{II}}+3\sqrt{3}i_{\text{II}}$	3*ρ*	0	0
*ɛ*_1_3__*^T^*^_1_^	*g*_0_	$-r_{\text{I}}+\sqrt{3}i_{\text{I}}+r_{\text{II}}-\sqrt{3}i_{\text{II}}$	−*ρ*	$2R_{n}+2\sqrt{3}I_{n}$	−2*i*_I_
*ɛ*_1_3__*^T^*^_2_^	*g*_0_	$-r_{\text{I}}+\sqrt{3}i_{\text{I}}+r_{\text{II}}-\sqrt{3}i_{\text{II}}$	−*ρ*	$-2R_{n}-2\sqrt{3}I_{n}$	2*i*_I_

2_3_					

*ɛ*_2_3__*^E^*	*g*_0_	$-r_{\text{I}}-\sqrt{3}i_{\text{I}}-3r_{\text{II}}-3\sqrt{3}i_{\text{II}}$	3*ρ*	0	0
*ɛ*_2_3__*^T^*^_1_^	*g*_0_	$-r_{\text{I}}-\sqrt{3}i_{\text{I}}+r_{\text{II}}+\sqrt{3}i_{\text{II}}$	−*ρ*	$2R_{n}-2\sqrt{3}I_{n}$	−2*i*_I_
*ɛ*_2_3__*^T^*^_2_^	*g*_0_	$-r_{\text{I}}-\sqrt{3}i_{\text{I}}+r_{\text{II}}+\sqrt{3}i_{\text{II}})$	−*ρ*	$-2R_{n}+2\sqrt{3}I_{n}$	2*i*_I_

**Table 13:** Splittings of *O* ⊃ *C*_2_(*i*_4)_ given sub-class structure.

*O* ⊃ *D*_4_ ⊃*C*_2_(*i*_4_)	0°	*r_n_*120°	*ρ_n_*180°	*R_n_*90°	*i_n_*180°

0_2_					

*ɛ*_0_2__*^A^*^_1_^	*g*_0_	4*r*_12_ + 4*r*_34_	2*ρ_xy_* + *ρ_z_*	4*R_xy_* + 2*R_z_*	4*i*_1256_ + *i*_3_ + *i*_4_
*ɛ*_0_2__*^E^*	*g*_0_	−2*r*_12_ − 2*r*_34_	2*ρ_xy_* + *ρ_z_*	−2*R_xy_* + 2*R_z_*	−2*i*_1256_ + *i*_3_ + *i*_4_
*ɛ*_0_2__*^T^*^_1_^	*g*_0_	−2*r*_12_ + 2*r*_34_	−*ρ_z_* 2*R_xy_*	−2*i*_1256_ − *i*_3_ + *i*_4_	
*ɛ*_0_2__*^T^*^_2_E__^	*g*_0_	2*r*_12_ − 2*r*_34_	−*ρ_z_*	−2*R_xy_*	2*i*_1256_ − *i*_3_ + *i*_4_
*ɛ*_0_2__*^T^*^_2_A_1___^	*g*_0_	0	−2*ρ_xy_* + *ρ_z_*	−2*R_z_*	*i*_3_ + *i*_4_

1_2_					

*ɛ*_1_2__*^A^*^_2_^	*g*_0_	4*r*_12_ + 4*r*_34_	2*ρ_xy_* + *ρ_z_*	−4*R_xy_* − 2*R_z_*	−4*i*_1256_ − *i*_3_ − *i*_4_
*ɛ*_1_2__*^E^*	*g*_0_	−2*r*_12_ − 2*r*_34_	2*ρ_xy_* + *ρ_z_*	2*R_xy_* − 2*R_z_*	2*i*_1256_ − *i*_3_ − *i*_4_
*ɛ*_1_2__*^T^*^_1_E__^	*g*_0_	2*r*_12_ − 2*r*_34_	−*ρ_z_*	2*R_z_* −	2*i*_1256_ + *i*_3_ − *i*_4_
*ɛ*_1_2__*^T^*^_1_A_2___^	*g*_0_	0	−2*ρ_xy_* + *ρ_z_*	−2*R_z_*	−*i*_3_ − *i*_4_
*ɛ*_1_2__*^T^*^_2_E__^	*g*_0_	−2*r*_12_ + 2*r*_34_	−*ρ_z_*	−2*R_xy_*	2*i*_1256_ + *i*_3_ − *i*_4_

**Table 14:** Matrix that converts tunneling strengths to cluster splitting energies.

0_2_	**1**	*r*_12_*, i*_1256_	*r*_34_*, R_xy_*	*ρ_xy_, R_z_*	*ρ_z_, i*_3_
*ɛ*_0_2__*^A^*^_1_^	1	4	4	2	1
*ɛ*_0_2__*^E^*	1	−2	−2	2	1
*ɛ*_0_2__*^T^*^_1_^	1	−2	2	0	−1
*ɛ_E,_*_0_2__*^T^*^_2_^	1	2	−2	0	−1
*ɛ_A_*__1__*_,_*_0_2__*^T^*^_2_^	1	0	0	−2	1

**Table 15:** Matrix that converts cluster splitting energies to tunneling strengths.

0_2_	*ɛ*_0_2__*^A^*^_1_^	*ɛ*_0_2__*^E^*	*ɛ*_0_2__*^T^*^_1_^	*ɛ_E,_*_0_2__*^T^*^_2_^	*ɛ_A_*__1__*_,_*_0_2__*^T^*^_2_^
**1**	$\frac{1}{12}$	$\frac{1}{6}$	$\frac{1}{4}$	$\frac{1}{4}$	$\frac{1}{4}$
*r*_12_*, i*_1256_	$\frac{1}{12}$	$-\frac{1}{12}$	$-\frac{1}{8}$	$\frac{1}{8}$	0
*r*_34_*, R_xy_*	$\frac{1}{12}$	$-\frac{1}{12}$	$\frac{1}{8}$	$-\frac{1}{8}$	0
*ρ_xy_, R_z_*	$\frac{1}{12}$	$\frac{1}{6}$	0	0	$-\frac{1}{4}$
*ρ_z_, i*_3_	$\frac{1}{12}$	$\frac{1}{6}$	$-\frac{1}{4}$	$-\frac{1}{4}$	$\frac{1}{4}$

## Appendix

### A. Classical *D*_3_ Modes: Local *C*_2_ and *C*_3_ Symmetry Examples

Local symmetry theory applies to classical vibrational modes as well as to quantum tunneling. Examples of classical *D*_3_ modes given below help clarify global-*vs*-local symmetry and geometry of group projection. For example, *D*_3_ modes defined by local *C*_2_(*i*_3_) in Figure 20 are to be compared with quantum waves in Figure 19. Each mode ket |*_jk_^α^*〉 has the same coefficients *D_jk_^α*^* (**g**) for projections in Equation (78) as the waves do, but the mode shapes clearly display a vector geometry.

In particular, global *x*-vector modes |*_xx_^E^*^_1_^〉 and |*_xy_^E^*^_1_^〉 (left *E*_1_ column in figure) “point” along global *x*-direction while *y*-vector modes |*_yx_^E^*^_1_^〉 and |*_yy_^E^*^_1_^〉 (right *E*_1_ column) “point” along global *y*-direction. Each global pair [|*_xℓ_^E^*^_1_^〉*,* |*_yℓ_^E^*^_1_^〉](*ℓ* = *x, y*) is projected to be an **i**_3_-symmetric-antisymmetric pair like lab unit vectors [|*x*〉,|*y*〉] (Recall Equation (78)).

(138) $$|x\rangle =P^{0_{3}}|1\rangle \;\sqrt{2}=(|1\rangle +|i_{3}\rangle )/\sqrt{2},\;\;\;|y\rangle =P^{1_{3}}|1\rangle \;\sqrt{2}=(|1\rangle -|i_{3}\rangle )/\sqrt{2}$$

This exposes easy derivations of *E*-irrep *D_jk_^E^*^_1_^ (**g**)=〈*j*|**g**|*k*〉 in Equation (89). Irreps in Equation (87) such as *D_jk_^E^*^_1_^ (**r**) for 120°-rotation **r** simply contain direction cosines 〈*j*|**r**|*k*〉=**ê***_j_*●**ê***_r_*_·_*_k_* of rotated vectors [**r**|*x*〉,**r**|*y*〉] relative to original [|*x*〉,|*y*〉] (Note transpose of equation array to matrix array).

(139) $$\begin{array}{c} r|x\rangle =-\frac{1}{2}|x\rangle +\frac{\sqrt{3}}{2}|y\rangle \\ r|y\rangle =-\frac{\sqrt{3}}{2}|x\rangle -\frac{1}{2}|y\rangle \end{array}\}implies:\{D^{E}(r)=\left(\begin{array}{cc} \langle x|r|x\rangle & \langle x|r|y\rangle \\ \langle y|r|x\rangle & \langle y|r|y\rangle \end{array}\right)=\left(\begin{array}{cc} -\frac{1}{2} & -\frac{\sqrt{3}}{2}\\ +\frac{\sqrt{3}}{2} & -\frac{1}{2} \end{array}\right)$$

This also fixes local transformations. Local *x*-vector modes |*_xx_^E^*^_1_^〉 and |*_yx_^E^*^_1_^〉 (lower *E*_1_ row in figure) “point” along local *x*-axes that are local *radial* lines while local *y*-vector modes |*_xy_^E^*^_1_^〉 and |*_yy_^E^*^_1_^〉 (upper *E*_1_ row) “point” along local *y*-axes that are local *angular* lines. If global symmetry meets local anti-symmetry as in |*_xy_^E^*^_1_^〉 (or *vice*-*versa* in |*_yx_^E^*^_1_^〉), a zero appears on the **i**_3_-axis in Figure 20. Singlet modes |*_xx_^A^*^_1_^〉 and |*_yy_^A^*^_2_^〉 avoid such conflicts by being all one or the other.

For group-defined cases like Figure 20, symmetry arguments alone determine normal modes that usually require diagonalizing a *K*-matrix (below) just as tunneling states (Figure 19) usually require diagonalizing an *H*-matrix.

#### A.1. Comparing K-Matrix and H-Matrix Formulation

Classical modes are eigenvectors of force-field matrix *K* or operator **K** that is a linear function of spring constants (*k*_0_, *etc*. in Figure 35) for a harmonic approximate potential *V* (**x**) that is a quadratic *K*-form of coordinates *x_a_* based on six*D*_3_-labeled axes **x̂***_a_* or |*a*〉 shown in Figure 20. Each **K** component *K_ab_*=〈*a*|**K**|*b*〉 is a sum over spring *k*-constants that connect axis-**x***^a^* to axis-**x***^b^* multiplied by factor (**k̂***_â_***x̂***_a_*)(**k̂***_b̂_***x̂***_b_*) for projecting spring *k*’s end vectors **k̂***_a_* and **k̂***_b_* onto **x̂***_a_* and **x̂***_b_* at respective connections. (A straight-line spring has equal **k̂***_a_*=**k̂***_b_*. Curvilinear springs must only have **k̂**-ends with equal sense (→→) or (←←) of spring direction. Either direction gives the same *K_ab_*).

(140) $$\begin{array}{l} V(x)=\sum_{\left(k\right)}\frac{1}{2}\langle x|K|x\rangle \;\;\;\text{where}:\;\;\;|x\rangle =\sum_{a}x^{a}|a\rangle ,\;\;\;(a,b)=(1,r^{1},r^{2},i_{1},i_{2},i_{3})\\ =\frac{1}{2}\sum_{a,b}K_{ab}x_{a}x_{a}\;\;\;\text{where}:\;\;\;K_{ab}=\{\begin{array}{ll} \sum_{\left(k\right)}\frac{k}{2}{\left({\hat{k}}_{a}•{\hat{x}}^{a}\right)}^{2} & if:a=b\\ -\sum_{\left(k\right)}k({\hat{k}}_{a}•{\hat{x}}^{a})({\hat{k}}_{b}•{\hat{x}}^{b}) & if:a\neq b \end{array} \end{array}$$

This sum of harmonic Hooke (*kx*^2^*/*2)-potentials has diagonal *K_aa_* terms followed by off-diagonal terms (*K_ab_*= *K_ba_*).

(141) $$\begin{array}{l} V(x)=\sum_{\left(k\right)}\frac{k}{2}{\left(\Delta \ell_{k}\right)}^{2}=\sum_{\left(k\right)}\frac{k}{2}\sum_{a,b}{\left({\hat{k}}_{a}•x^{a}-{\hat{k}}_{b}•x^{b}\right)}^{2}\\ =\sum_{\left(k\right)}\frac{k}{2}\sum_{a}{\left({\hat{k}}_{a}•{\hat{x}}^{a}\right)}^{2}x_{a}^{2}-\sum_{\left(k\right)}k\sum_{a\neq b}({\hat{k}}_{a}•{\hat{x}}^{a})({\hat{k}}_{b}•{\hat{x}}^{b})x_{a}x_{b} \end{array}$$

The classical equation of coupled harmonic motion is a Newtonian **F**= **M**·**a** relation of a *n*-dimensional force vector **F**, acceleration vector **a**, and mass operator **M**. The latter is a unit-matrix-multiple *M*·**1** for the *D*_3_-symmetric case treated here. The driving force **F** is a (-)derivative of potential Equation (140) that becomes a **K**-matrix expression.

(142) $$-M\partial_{t}^{2}x^{a}=\frac{\partial V}{\partial x^{a}}=\sum_{b}K_{ab}x^{b}$$

It is instructive to compare this classical equation of motion to that of Schrodinger’s equation for quantum motion.

(143) $$i\hbar \partial_{t}\psi^{a}=\sum_{b}H_{ab}\psi^{b}$$

Squaring quantum time generator *iħ∂_t_*=**H** yields equations having classical form Equation (142) with *K* = *H*^2^ and *M*=*ħ*^2^.

(144) $$-\hbar^{2}\partial_{t}^{2}\psi^{a}=\sum_{b}K_{ab}\psi^{b}\;\text{where}:K=H^{2}$$

The (**H***/ħ*)-eigenvalues are quantum angular frequencies *ε_m_/ħ* =*ω_m_*. The (**K***/M*)-eigenvalues are classical *squared* angular frequencies *k_m_/M*=*ω_m_*^2^. The former is Planck’s oscillator frequency relation *ε*= *ħω*. The latter is Hooke’s relation *k/M*=*ω*^2^. Apart from normalization, eigenvectors of quantum *H* are identical to those of classical *K* and either eigenvalue set corresponds to the respective energy spectrum.

#### A.2. Comparing K-Matrix and H-Matrix Eigensolutions for Local *D*_3_⊃*C*_2_(*i*_3_)

The preceding relates eigensolutions Equations (92) and (93) of quantum Hamiltonian *H*-matrix in (71) with those of a classical *K*-matrix. In particular, eigenvectors of *H* found using *D*-matrices in Equation (89) or (139) also serve as mode-eigenkets in Figure 20 that diagonalize a *D*_3_⊃*C*_2_(*i*_3_)-locally-symmetric *K*-matrix. With this symmetry, *K* cannot couple radial (local-*x*) and angular (local-*y*) modes and is left with just four independent real group-based parameters *g_a_*=*r*_0_, *r*_1_, *i*_12_, and *i*_3_ allowed for *D*_3_⊃*C*_2_(*i*_3_)-symmetric *H* in Equation (93). These relate to four spring *k_h_*-constants in Figure 35a.

Only 1*^st^*-row parameters *g_b_*=〈**1**|**K**|**g***_b_*〉=*K*_1_*_b_* of the force matrix *K_ab_* are needed for the spring model in Figure 35a. That model includes *k_r_*(angular) and *k_i_*(radial) constants for internal connections between masses. The *k*_3_(angular) and *k*_0_(radial) constants represent external connections between each mass and an outside lab frame.

Generic group parameters *g_b_*=*H*_1_*_b_*, labeled [*r*_0_*, r*_1_*, r*_2_*, i*_1_*, i*_2_*, i*_3_] for the *H*-matrix in Equation (71), are now applied to *g_b_*=*K*_1_*_b_*. The *g_b_* are to be related to spring-constants *k_j_* using coordinate-spring projection cosine factors (**k̂**_1_ ● **x̂**_1_)( **k̂***_b_* ● **x̂***_b_*) in Equations (140) and (141). The usual harmonic limit assumes small vibrational amplitudes (*x_b_*≪1) for which direction of spring end vectors **k̂**_1_ or **k̂***_b_* do not vary to 1*^st^*-order, and so, for lab-fixed **x̂***_a_* the *K_ab_* are constants.

(145) $$\begin{array}{ccccccc} |g_{b}\rangle & |1\rangle & |r^{1}\rangle & |r^{2}\rangle & |i_{1}\rangle & |i_{2}\rangle & |i_{3}\rangle \\ & k_{i}/2 & k_{i}/2 & k_{i}/2 & k_{i}/2 & k_{i}/2 & k_{i}/2\\ \langle 1|K|g_{b}\rangle = & +k_{r} & -k_{r}/2 & -k_{r}/2 & +k_{r}/2 & +k_{r}/2 & -k_{r}\\ & +k_{3} & +0 & +0 & +0 & +0 & -k_{3}\\ & +k_{0}/2 & +0 & +0 & +0 & +0 & +k_{0}/2 \end{array}$$

One may visualize each −*K*_1_*_b_* as the acceleration of *x*_1_ due to setting a (tiny) unit *x_b_* in Equation (142). Diagonal -*K*_11_ must be negative or else *x*_1_ blows up. Higher order *anharmonic* terms are needed to describe effects of rotating **k̂***^b^* or **x̂***^b^* and such models are likely to suffer from classical stochastic (chaotic) motion.

Substitution of generic *g_a_* from Equation (145) into reduced *D*_3_⊃*C*_2_(*i*_3_)-symmetric *H*-matrix in Equation (92) or (93) gives *K*-matrix eigenvalues *K_ℓℓ_^α^* due to each spring *k_i_*, *k_r_*, *k*_3_, or *k*_0_ in Figure 35a separately or together. Modes in Figure 20 remain *eigen*modes for all values of four spring constants *k_i_*, *k_r_*, *k*_3_, and *k*_0_ since none can mix local *x*-and-*y*-symmetry.

(146) $$\begin{array}{ccc} K_{xx}^{A_{1}}= & r_{0}+r_{1}+r_{1}^{*}+i_{1}+i_{2}+i_{3} & =k_{0}+3k_{i}\\ k_{yy}^{A_{2}}= & r_{0}+r_{1}+r_{1}^{*}-i_{1}-i_{2}-i_{3} & =3k_{3}\\ \left(\begin{array}{cc} K_{xx}^{E} & K_{xy}^{E}\\ K_{yx}^{E} & K_{yy}^{E} \end{array}\right)= & \frac{1}{2}\left(\begin{array}{cc} 2r_{0}-r_{1}-r_{1}^{*}-i_{1}-i_{2}+2i_{3} & \sqrt{3}(-r_{1}+r_{1}^{*}-i_{1}+i_{2})\\ \sqrt{3}(-r_{1}^{*}+r_{1}-i_{1}+i_{2}) & 2r_{0}-r_{1}-r_{1}^{*}+i_{1}+i_{2}-2i_{3} \end{array}\right) & =\left(\begin{array}{cc} k_{0} & 0\\ 0 & k_{3}+2k_{r} \end{array}\right) \end{array}$$

Any set of four *K*-matrix eigenvalues *k^A^*^_1_^, *k^A^*^_2_^, *k_x_^E^*, and *k_y_^E^* is arithmetically possible by adjusting the four spring constants. However, their arrangement in Figure 20 (this was drawn to match tunneling states in Figure 19) is impossible without negative *k*-values that would give classical instability. As shown below, free ring molecules often have *A*_1_-stretching modes among the highest frequencies. In contrast, tunneling amplitudes are often negative so their *A*_1_ states lie low. As a rule, fewer quantum nodes imply lower energy.

#### A.3. K-Matrix Eigensolutions for Broken Local Symmetry

In some ways the direct-*k*_1_-connection spring model of Figure 35b is quite the opposite of the *D*_3_⊃*C*_2_(*i*_3_) model just treated since it involves maximal (50-50) mixing of *x* and *y* local symmetry. Below are recalculated generic *g_b_*=〈**1**|**K**|**g***_b_*〉 in terms of direct spring-constants *k*_1_ using (141) with projection cosines listed in Figure 35b.

(147) $$\begin{array}{lllllll} |g_{b}\rangle & |1\rangle & |r^{1}\rangle & |r^{2}\rangle & |i_{1}\rangle & |i_{2}\rangle & |i_{3}\rangle \\ & k_{1}({\text{cos}}^{2}\;75{}^{\circ} & k_{1}\;\text{cos }75{}^{\circ} & k_{1}\;\text{cos }15{}^{\circ} & k_{1}\;\text{cos }15{}^{\circ} & k_{1}\;\text{cos }75{}^{\circ} & k_{1}({\text{cos}}^{2}\;75{}^{\circ}\\ \langle 1|K|g_{b}\rangle = & +{\text{cos}}^{2}\;15{}^{\circ}) & \cdot \text{cos }15{}^{\circ} & \cdot \text{cos }75{}^{\circ} & \cdot \text{cos }15{}^{\circ} & \cdot \text{cos }75{}^{\circ} & -{\text{cos}}^{2}\;15{}^{\circ})\\ & =k_{1} & =\frac{k_{1}}{4} & =\frac{k_{1}}{4} & =\frac{k_{1}(2-\sqrt{3})}{4} & =\frac{k_{1}(2+\sqrt{3})}{4} & =\frac{k_{1}}{2} \end{array}$$

Again, a substitution of generic *g_a_* from Equation (147) into reduced *H*-matrix Equation (93) gives a reduced *K*-matrix like Equation (146), but now the *E*-symmetry submatrix is not diagonal.

(148) $$\begin{array}{cc} K_{xx}^{A_{1}}= & 3k_{1}\\ K_{yy}^{A_{2}}= & 0\\ \left(\begin{array}{cc} K_{xx}^{E} & K_{xy}^{E}\\ K_{yx}^{E} & K_{yy}^{E} \end{array}\right)= & \left(\begin{array}{cc} \frac{3k_{1}}{4} & \frac{3k_{1}}{4}\\ \frac{3k_{1}}{4} & \frac{3k_{1}}{4} \end{array}\right) \end{array}$$

Eigenvectors of the *E*-submatrix are symmetric (+) and antisymmetic (−) mixtures of *x* and *y* local symmetry states.

(149) $$\begin{array}{l} K|\begin{array}{c} E\\ g(-) \end{array}\rangle =K\left(|\begin{array}{c} E\\ gx \end{array}\rangle -|\begin{array}{c} E\\ gy \end{array}\rangle \right)\frac{1}{\sqrt{2}}=\frac{3k_{1}}{2}|\begin{array}{c} E\\ g(-) \end{array}\rangle ,\\ K|\begin{array}{c} E\\ g(+) \end{array}\rangle =K\left(|\begin{array}{c} E\\ gx \end{array}\rangle +|\begin{array}{c} E\\ gy \end{array}\rangle \right)\frac{1}{\sqrt{2}}=0|\begin{array}{c} E\\ g(+) \end{array}\rangle ,\;\;\;g=(x,y). \end{array}$$

Figure 36 shows (50-50 ±)-mixing due to *k*_1_. It distinguishes genuine vector modes (|*_x,_*_(−)_*^E^*〉 or |*_y,_*_(−)_*^E^*〉) and the scalar breathing mode (|*_x,x_^A^*^_1_^〉) from non-genuine (low or zero-frequency) vector modes of pure *x* or *y*-translation (|*_x,_*_(+)_*^E^* 〉 or |*_y,_*_(+)_*^E^* 〉) and rigid rotation (pseudo-scalar |*_y,y_^A^*^_2_^〉). The *i*_3_-local symmetry is wiped out by direct connection-*k*_1_.

In order to reestablish approximate *D*_3_⊃*C*_2_(*i*_3_)-local-symmetry there needs to be a *C*_2_(*i*_3_)-“*locale*” provided by lab-grounded potential springs such as those with constants *k*_3_ and *k*_0_ in Figure 35a. Adding these in the form of Equation (146) to Equation (148) causes a transition between the two extremes. If the difference (*k*_3_ + 2*k_r_* − *k*_0_) between eigenvalues Equation (146) begins to dominate the off-diagonal component (3*k*_1_*/*4) of Equation (148), then mixed *E*-modes of Figure 36 begin to recover *D*_3_⊃*C*_2_(*i*_3_) locality seen in Figure 20.

Meanwhile the constant *k*_3_ that determines eigenvalue *k_y,y_^A^*^_2_^ does not affect locality for either of the singlet *A*_1_ or *A*_2_ modes. Singlet eigenvectors are non-negotiable as long as master symmetry *D*_3_ holds.

#### A.4. K-Matrix Eigensolutions for *D*_3_⊃*C*_3_ Symmetry

Another choice for *D*_3_ local symmetry is the *C*_3_ subgroup of Equation (79) corresponding to a strong chiral perturbation by internal rotation, spin, or *B*-field. The *E*-submatrix in Equation (146) with zero generic reflection parameters (*i*_1_=*i*_2_=*i*_3_=0) may take a purely chiral *C*_3_ form if the generic rotation parameters *r*_1_ and *r*_2_=*r*_1_*^*^* are purely imaginary corresponding to velocity dependent force ( *r*_1_=*ir* and *r*_2_=−*ir*. Here *K* is assumed Hermitian self-conjugate as was *H*).

(150) $$\begin{array}{cc} K_{xx}^{A_{1}}= & r_{0}\\ K_{yy}^{A_{2}}= & r_{0}\\ \left(\begin{array}{cc} K_{xx}^{E} & K_{xy}^{E}\\ K_{yx}^{E} & K_{yy}^{E} \end{array}\right)= & {\left(\begin{array}{cc} r_{0} & -ir\sqrt{3}\\ +ir\sqrt{3} & r_{0} \end{array}\right)}_{\begin{array}{c} r_{1}=ir=-r_{2}^{*}\\ i_{1}=i_{2}=i_{3}=0 \end{array}} \end{array}$$

*C*_3_*E*-eigenvectors have local *x* ± *iy*=(±1)_3_ combinations that exhibit purely circular right *R*=(+1)_3_ and left *L*=(−1)_3_ polarization orbits of *C*_3_ symmetry shown in Figure 37 (Recall *C*_3_ splitting in Equation (79)).

(151) $$\begin{array}{l} K|\begin{array}{c} E\\ g{\left(+1\right)}_{3} \end{array}\rangle =K\left(|\begin{array}{c} E\\ gx \end{array}\rangle +i|\begin{array}{c} E\\ gy \end{array}\rangle \right)\frac{1}{\sqrt{2}}=+r\sqrt{3}|\begin{array}{c} E\\ g{\left(+1\right)}_{3} \end{array}\rangle ,\\ K|\begin{array}{c} E\\ g{\left(-1\right)}_{3} \end{array}\rangle =K\left(|\begin{array}{c} E\\ gx \end{array}\rangle -i|\begin{array}{c} E\\ gy \end{array}\rangle \right)\frac{1}{\sqrt{2}}=-r\sqrt{3}|\begin{array}{c} E\\ g{\left(-1\right)}_{3} \end{array}\rangle ,\;\;\;g=(x,y). \end{array}$$

Pure *C*_3_ symmetry is a normal subgroup and restricts *k_x,x_^A^*^_1_^ and *k_y,y_^A^*^_2_^ to become degenerate. Both the scalar |_03_*_,_*_03_*^A^*^_1_^〉 and pseudoscalar |_03_*_,_*_03_*^A^*^_2_^〉 state are both labeled equally by (0)_3_ symmetry. Local symmetry effectively goes global in the pure *C*_3_-case where all internal coupling is zero.

Any internal or external parameters may split the *A*_1_-*A*_2_ degeneracy and mix the *C*_3_ states to form elliptical polarization orbits. This is most efficiently calculated using U(2) analysis similar to Equation (137).
